# Distinct region-specific neutralization profiles of contemporary HIV-1 clade C against best-in-class broadly neutralizing antibodies

**DOI:** 10.1128/jvi.00008-25

**Published:** 2025-05-16

**Authors:** Jyoti Sutar, Priyanka Jayal, Ranajoy Mullick, Sangeeta Chaudhary, Prajakta Kamble, Shilpa Bhowmick, Snehal Kaginkar, Varsha Padwal, Pratik Devadiga, Namrata Neman, Dale Kitchin, Haajira Kaldine, Nonhlanhla N. Mkhize, Bongiwe Ndlovu, Kamini Gounder, Sohini Mukherjee, Shweta Shrivas, Neha Sharma, Chaman Prasad, Sonia Tewatia, Nainika Parihar, Naresh Kumar, Nandini Kasarpalkar, Balwant Singh, Shobha Mohapatra, Mohammad Aquil, C. Vishal Kumar, Thongadi Ramesh Dinesha, Aylur Kailasom Srikrishnan, Jayanthi Shastri, Sachee Agrawal, Sushma Gaikwad, Sayantani Mondal, Bhaswati Bandyopadhyay, Subhasish Kamal Guha, Dipesh Kale, Debasis Biswas, Dhanashree Patil, Ramesh S. Paranjape, Satyajit Mukhopadhyay, Hema, Ritika Das, Anand Kondapi, Vikrant Bhor, Suprit Deshpande, Devin Sok, Thumbi Ndung’u, Penny L. Moore, Kailapuri Gangatharan Murugavel, Vainav Patel, Jayanta Bhattacharya

**Affiliations:** 1 Antibody Translational Research Program, Center for Virus Research, Vaccines & Therapeutics, BRIC- Translational Health Science & Technology Institute, NCR Biotech Science Cluster, Faridabad, Haryana, India; 2 IAVI, Gurugram, Haryana, India; 3 IAVI, New York, New York, USA; 4 Molecular and Translational Virology Unit, Center for Virus Research, Vaccines & Therapeutics, BRIC- Translational Health Science & Technology Institute, NCR Biotech Science Cluster, Faridabad, Haryana, India; 5 ICMR- National Institute of Research in Reproductive & Child Health, Mumbai, Mahara shtra, India; 6 Antibody Immunity Research Unit, University of the Witwatersrand, Johannesburg, South Africa; 7 National Institute for Communicable Diseases of the National Health Laboratory Services, Johannesburg, South Africa; 8 HIV Pathogenesis Programme, The Doris Duke Medical Research Institute, University of KwaZulu-Natal, Durban, South Africa; 9 Africa Health Research Institute, Durban, South Africa; 10 Y R Gaitonde Center for AIDS Research & Education, Chennai, Tamil Nadu, India; 11 Topiwala National Medical College and Bai Yamunabai Laxman Nair Charitable Hospital, Mumbai, Maharashtra, India; 12 School of Tropical Medicine, Kolkata, West Bengal, India; 13 Department of Microbiology, All India Institute of Medical Sciences, Bhopal, Madhya Pradesh, India; 14 Dr Prabhakar Kore Basic Science Research Center, KLE Academy of Higher Education and Research, Belagavi, Karnataka, India; 15 Department of Biotechnology & Bioinformatics, School of Life Sciences, University of Hyderabad, Hyderabad, Telangana, India; 16 IAVI-Neutralizing Antibody Center, The Scripps Research, La Jolla, California, USA; 17 Global Health Investment Corporation, New York, New York, USA; 18 Ragon Institute of Massachusetts General Hospital, Massachusetts Institute of Technology and Harvard University, Cambridge, Massachusetts, USA; 19 Division of Infection and Immunity, University College London, London, United Kingdom; 20 Centre for the AIDS Programme of Research in South Africa (CAPRISA), University of KwaZulu Natal, Durban, South Africa; 21 CEPI Central Laboratory Network (CLN), Bioassay Laboratory, BRIC- Translational Health Science & Technology Institute, NCR Biotech Science Cluster, Faridabad, Haryana, India

**Keywords:** HIV-1, bnAb, envelope, neutralizing antibodies, clade C, India, South Africa, contemporary virus, prevention, genetic diversity

## Abstract

While broadly neutralizing antibodies (bnAbs) have been clinically shown to prevent HIV-1 acquisition, their relative effectiveness against regionally relevant HIV-1 forms is not clear. In the present study, we examined the extent of neutralization susceptibility of contemporary HIV-1 Indian clade C at a population level along with a head-to-head comparison with that from South Africa against a panel of clinically relevant best-in-class bnAbs. Env-pseudotyped viruses encoding HIV-1 India clade C *env* were found to be best neutralized by the V3 glycan-directed bnAbs (10-1074 and BG18) and select CD4 binding site (CD4bs)-directed bnAbs (VRC07, N6, and 1-18); however, they demonstrated significant resistance to V1/V2 apex-directed bnAbs. Interestingly, the magnitude of the neutralization sensitivity differed between contemporary India and South Africa clade C. Neutralization resistance to key bnAbs was observed to be associated with differences in residues on Env that form bnAb contact sites, gp120 loop lengths, and potential N-linked glycans. Notably, the second generation CD4bs bnAbs (VRC07, N6, 1-18) showed neutralization of VRC01- and 3BNC117-resistant viruses but with two-to sevenfold reduced potency compared to the VRC01-sensitive counter-parts, likely due to the enrichment of resistance-associated residues observed in loop D. Predictive analysis indicated that the combination of BG18, N6, and PGDM1400 can provide over 95% neutralization coverage of contemporary India clade C at 1 μg/mL (IC80), an observation distinct from that observed with Africa clade C. Our study clearly highlights that both the complementarity of bnAb classes and the regionally relevant HIV-1 forms are important in achieving clinical effectiveness.

HIV, having complex and evolving diversity (1), remains a global health priority with over 39 million people currently infected globally, 2.4 million of which reside in India, making India the third largest HIV epidemic globally (2, 3). The high genetic variability of globally circulating HIV, both between and within an individual, has been a major roadblock in designing an effective preventive intervention despite significant efforts (4). While antiretroviral therapy (ART) has been successful in the treatment of HIV and slowing down the spread of the initial pandemic, rising global resistance to available antiretrovirals necessitates the expansion of available therapeutics and has reinvigorated efforts to design effective vaccines (5). In the absence of an efficacious vaccine against HIV, passively administered broadly neutralizing monoclonal antibodies (bnAbs) along with effective antiretroviral drugs (ARV) could play a significant role in reducing incidence in high-risk groups and key populations (6). While efforts toward developing vaccine immunogens capable of inducing bnAbs are ongoing, the recently conducted phase 2B Antibody-Mediated Prevention (AMP) efficacy trials (HVTN 703/HPTN 084 and HVTN 704/HPTN 085) demonstrated that a passively administered bnAb could prevent infection by bnAb-sensitive viruses (7, 8). The AMP trial data indicated that the biomarker PT_80_, which integrates serum concentration of bnAbs with the *in vitro* measure of 80% inhibition (IC80), could be used to predict the preventive efficacy of bnAbs. A sustained PT_80_ of >200 against 90% of circulating viruses was identified as a minimum benchmark for design and evaluation of effective bnAb regimens (8). The study also highlighted that a combination of bnAbs would be required for optimal coverage of globally circulating HIV-1 subtypes toward capturing those that are resistant to one bnAb class but sensitive to another. HIV-1 clade C, which is the major globally circulating form, also forms the bulk of infections in South Africa and India, although genetic and functional data pertaining to contemporary HIV-1 forms from India are very limited. HIV-1 evolution over time is believed to contribute to changes in *env* sequence, the sole target of bnAbs, that will likely impact the consistencies with the breadth and potency of bnAbs with clinical relevance to effectively tackle currently circulating forms globally (9–11).

Previous studies provided evidence of HIV-1 clade B and non-India C viruses becoming increasingly resistant to select bnAbs over time (9, 11–15). Our previous study, using limited historical (obtained prior to 2014) HIV-1 clade C of Indian origin (10), indicated significant variation in their susceptibility to bnAbs and also indicated that evolving viruses were becoming increasingly resistant to key bnAbs such as CAP256-VRC26.25. In the present study, we examined neutralization profiles of 115 HIV-1 clade C as pseudoviruses encoding full-length *env* (*gp160*) isolated between 2020 and 2023 (contemporary) from nine geographically distinct regions in India, compared with that from South Africa, and examined the best-in-class bnAbs that would provide optimal neutralization coverage of contemporary India clade C viruses. The *env* sequence diversity and neutralization profiles of contemporary India clade C were also compared with those of South African origin. We believe that the neutralization profiles and *in vitro* inhibitory concentrations (IC80) identified in this study could be integrated into the PT_80_ biomarkers to evaluate bnAb regimens with the highest preventive efficacy against the contemporary viruses in India.

## Results

### Phylogenetic profiles of contemporary HIV-1 clade C from different geo-graphical regions of India

**Fig 1:**
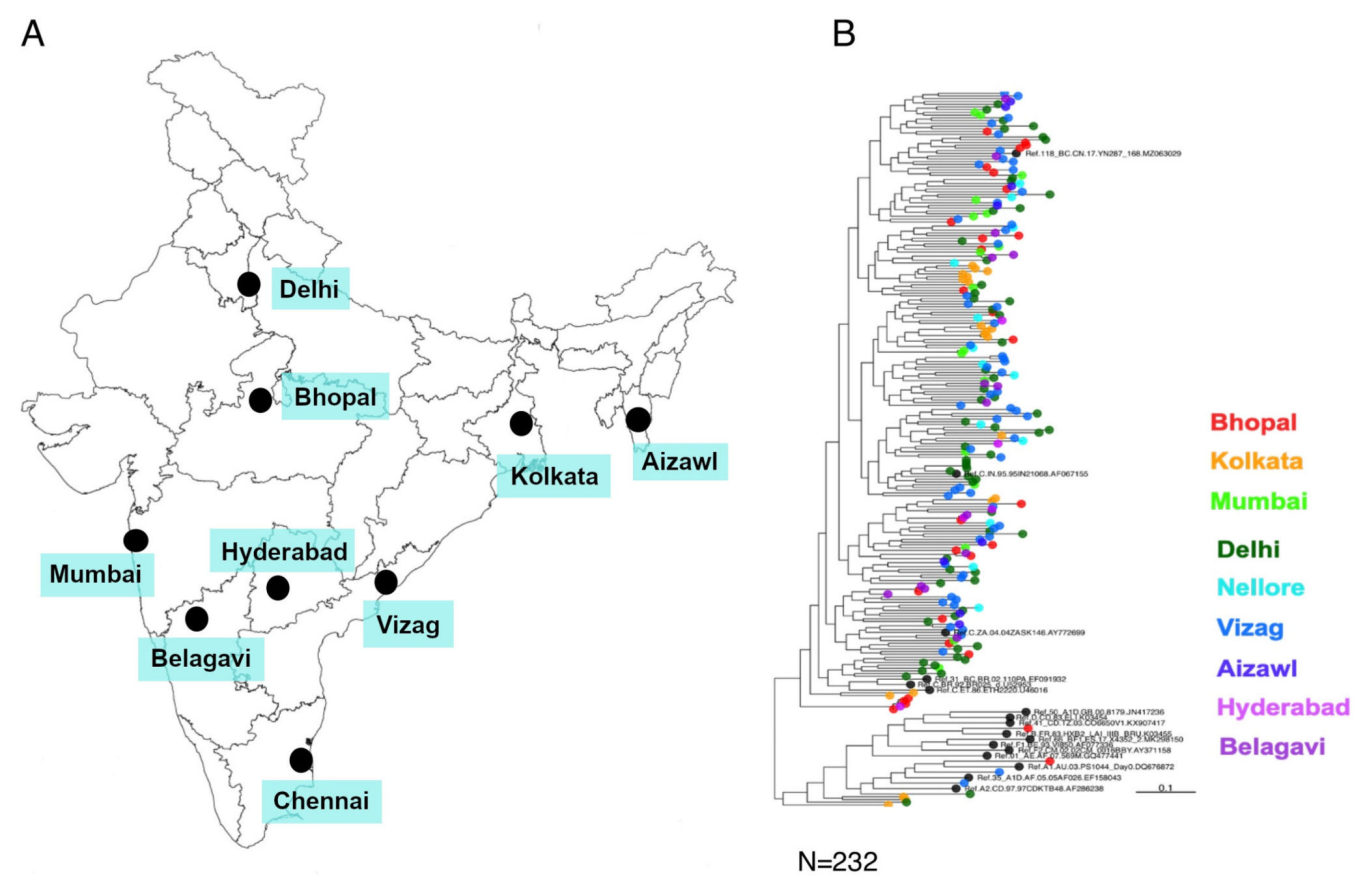
Phylogenetic relatedness of the contemporary HIV-1 India clade C at the population level. (A) Surveillance sites built and samples collected across different geographical sites in India between 2020 and 2023. The map was sourced from the map reported by the Department of Science and Technology, Government of India, at https://surveyofindia.gov.in/pages/outline-maps-of-india. (B) Phylogenetic relatedness of HIV-1 clade C Env proteins representing circulating forms in different geographic regions. Phylogenetic trees were generated for 249 HIV-1 envelope amino acid sequences, which included 232 contemporary (obtained between 2020 and 2023) from India and 17 HIV-1 group M reference sequences. These sequences were aligned using MAFFT, and the alignment was manually curated in BioEdit v.7.2.5. The phylogenetic tree was constructed with IQ-TREE under the HIVb model with estimated γ parameters and number of invariable sites. The robustness of the tree topology was further assessed by SH-aLRT as well as 1,000 ultrafast bootstrap replicates implemented in IQ-TREE.

We first examined the phylogenetic relationship of the *env* gene of the contemporary viruses from different geographical regions. We obtained unique full-length *env* (*gp160*) sequences from 232 individuals from nine geographically distinct sites in India between 2020 and 2023 (Fig. 1A). These include sequences obtained from ART-naïve early seroconverters and from individuals on ART (Table S1). The PCR-amplified products were processed for high-throughput deep sequencing using Oxford Nanopore Technology and Illumina-based NGS platforms to obtain long- and short-read sequences. A consensus *env* sequence, which was the major circulating variant for each individual, was constructed based on multiple alignment of both short- and long-read sequences to ensure inclusion of the accurate dominant *env* sequences for further analysis. Phylogenetic analysis was performed using these contemporary Indian *env* sequences along with 17 HIV-1 group M reference sequences (hiv.lanl.gov), shown in Fig. 1B. No region-specific phylogenetic clustering was observed. Interestingly, we identified five subtype A1, one subtype B, and four A1/C recombinants (Table S1). Furthermore, *pol* gene sequencing of HIV+ RNA obtained from the therapy-naïve individuals showed that 11% of them contain major (>50% variant frequency in deep sequencing data) drug resistance-associated mutations (DRMs) with reverse transcriptase (RT)-associated DRM found to be more prevalent compared to protease and integrase inhibitor-associated DRMs (Table S1). Our observation provides evidence of the establishment of infection by drug-resistant HIV-1.

### Contemporary HIV-1 India clade C demonstrated significant resistance to V1/V2-directed antibodies compared to those that target CD4 binding and V3 supersites

**Fig 2:**
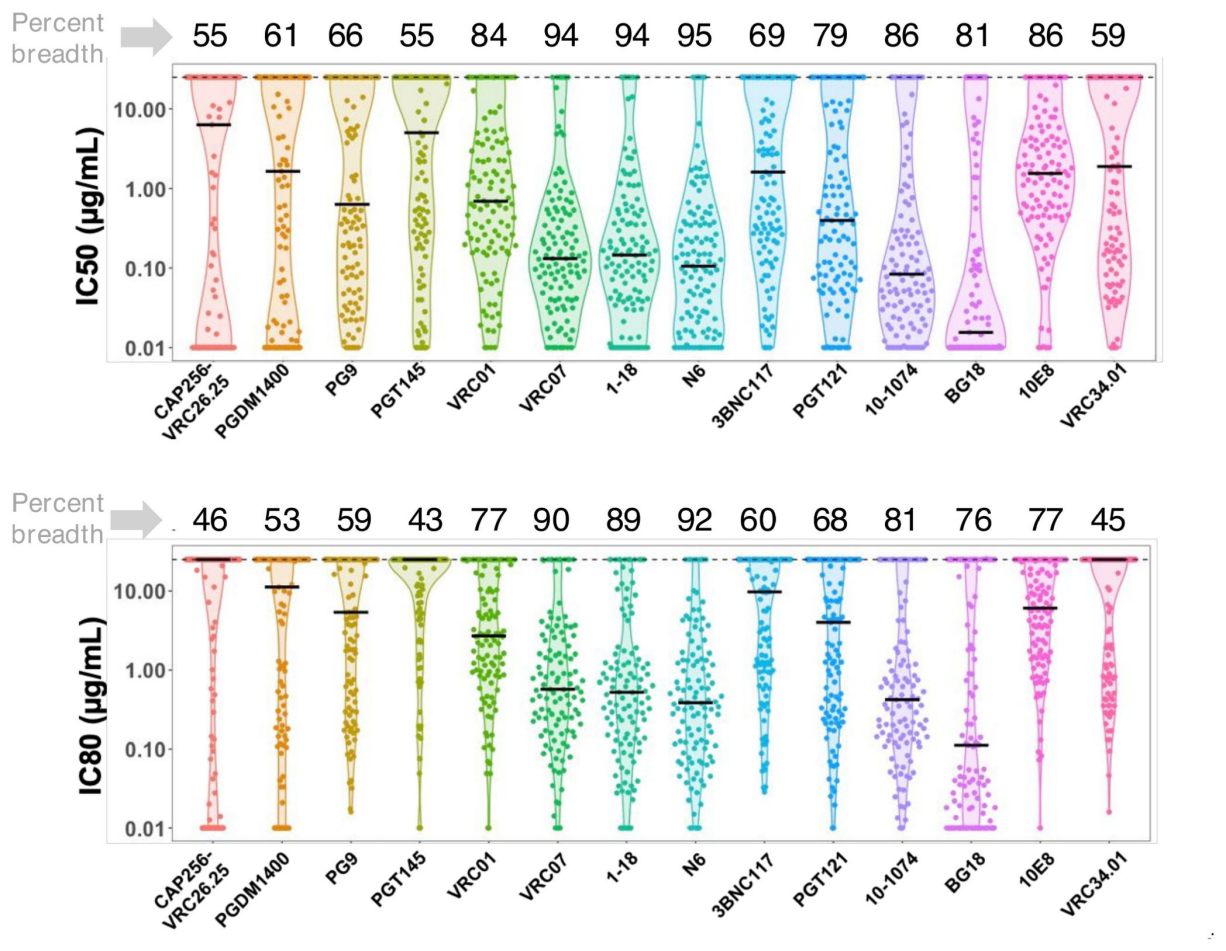
Neutralization profiles of contemporary HIV-1 India clade C to best-in-class existing bnAbs. Pseudoviruses expressing 115 contemporary *envs* obtained from individuals representing nine geographically distant regions in India and comprising distinct risk groups were assessed for their degree of susceptibility to 14 bnAbs as indicated having distinct epitope specificities on viral Env. IC50 and IC80 refer to the IgG concentrations (μg/mL) at which pseudoviruses demonstrated 50% and 80% neutralizations, respectively. Pseudoviruses that were not neutralized up to 25 μg/mL of IgG were considered as resistant viruses. Neutralization assay was carried out at least three times in duplicate, and the average was used to plot the graph. Neutralization breadth of each bnAb expressed as percent neutralization by IgG up to 25 μg/mL is shown on top of each graph (upper and lower panel).

Next, we examined the neutralization profiles of the contemporary viruses against a large panel of bnAbs with distinct specificities. We randomly selected 115 unique sequences representative of all the nine geographically distinct sites in India and prepared pseudoviruses expressing these unique *env* sequences (Table 1), which were further examined against 14 bnAbs of distinct epitope specificities in viral Env. All of these envelopes were found to be CCR5 tropic. As shown in Fig. 2, pseudo-viruses expressing contemporary clade C *envs* showed broad sensitivity to bnAbs targeting CD4 binding site (CD4bs) and V3 glycan supersite, compared to the ones that target V1/V2 apex. Among bnAbs targeting CD4bs-directed bnAbs, N6, 1-18, and VRC07 showed >90% of viruses neutralized with IC50/IC80 values <25 μg/mL, respectively, and over 78% and 65% of the panel viruses neutralized with IC50/IC80 values <1 μg/mL, respectively) (Fig. 2; Table S2). Among the V3 glycan supersite-directed bnAbs, 10-1074 and BG18 were observed to demonstrate maximal neutralization breadth (>80% and >70% of the panel viruses neutralized with IC50 and IC80 values <25 μg/mL by these bnAbs while over 81% and 74% of the panel viruses neutralized with IC50 and IC80 values <1 μg/mL). Among all the bnAbs examined, N6 (demonstrated >94% coverage with IC80 of 0.44 μg/mL) and 10-1074 (demonstrated >80% coverage with IC80 of 0.63 μg/mL) were found to be most broad, while BG18 (IC80 of 0.29 μg/mL) was found to be most potent. While we identified several clade C viruses with class-specific resistance (Fig. S1), V1/V2-directed bnAb class-specific resistant viruses were relatively common (Table S2). A total of 45% and 40% contemporary viruses were found to be resistant to CAP256-VRC26.25 and PGDM1400, respectively. We also identified viruses with resistance to best-in-class bnAbs that target the CD4bs (VRC01, 3BNC117, VRC07, N6, and 1-18) and V3 supersite (PGT121, BG18, and 10-1074). Specifically, we identified *env* sequences isolated from four unique donors, which, when expressed as pseudoviruses (Table S2; Fig. S1), demonstrated complete resistance to all the CD4bs-directed bnAbs tested in our study; VRC01, VRC07, N6, and 1-18. For V3 glycan supersite-directed bnAbs, we identified eight unique donors, *envs* which demonstrated complete resistance to PGT121, 10-1074, and BG18 (Table S2; Fig. S1). Moreover, among these individuals, we identified *env* sequences from six individuals, which, when expressed as pseudovirus, demonstrated very broad resistance to the majority of the class-specific bnAbs tested in this study (Table 2).

**Fig 3:**
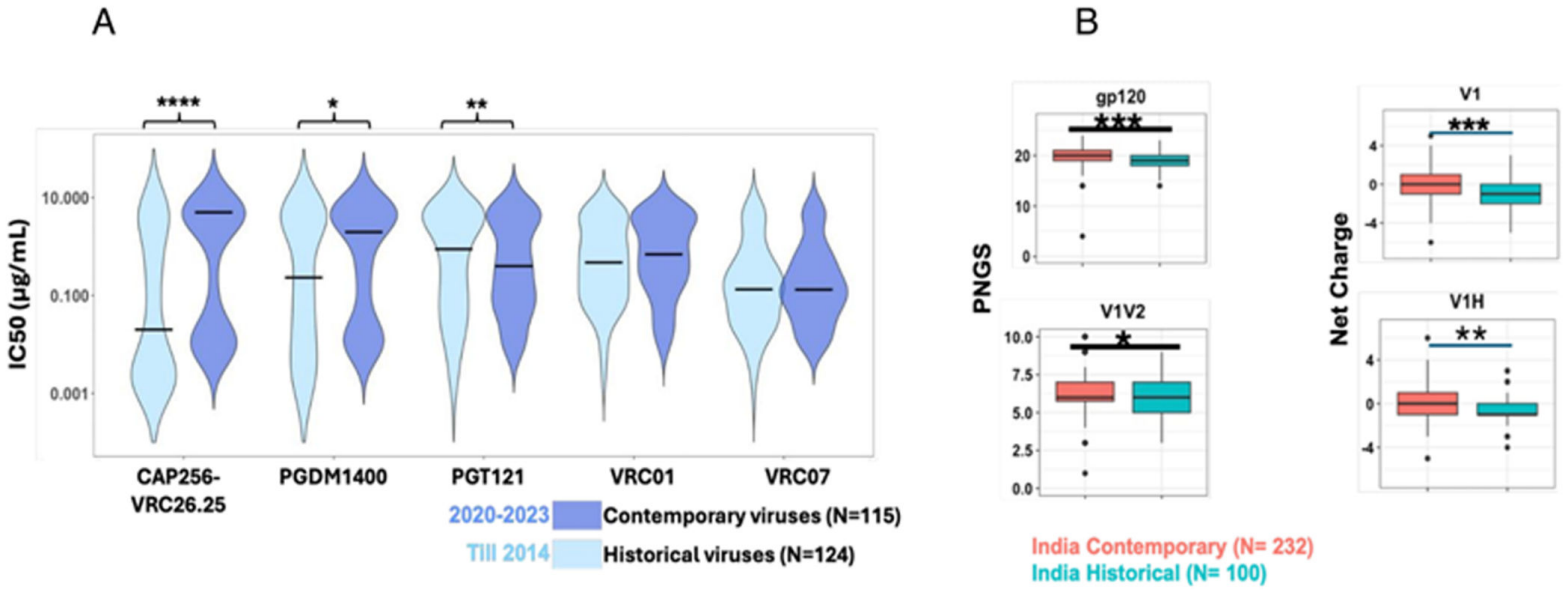
Comparison of neutralization sensitivity to key bnAbs between historic and contemporary India clade C. (A) Degree of neutralization susceptibility of historical (*N* = 124; obtained before 2014) and contemporary viruses (*N* = 115; obtained between 2020-2023) assessed by pseudovirus neutralization assay. IC50 value of 25 μg/mL was considered as the neutralization sensitivity threshold. Statistical analysis to assess significance (*P*-values) of differences in neutralization sensitivity to a given bnAb by pseudoviruses expressing both historical and contemporary *envs* was performed by Mann-Whitney U-test. Neutralization assay was repeated at least three times in duplicate, and the average was used to plot the graph. (B) *gp120* variable loop characteristics of historical and contemporary *env* sequences were assessed using the “variable characteristics tool” hosted at the Los Alamos National Laboratory HIV database (LANL-HIVDB, https://www.hiv.lanl.gov/content/sequence/VAR_REG_CHAR/index.html). Potential N-linked glycosylation sites prediction was performed with the tool N-Glycosite atLANL-HIVDB (https://www.hiv.lanl.gov/content/sequence/GLYCOSITE/glycosite.html). Statistical significance was assessed by the Mann-Whitney U-test. *P*-values between 0.05–0.01, 0.01–0.001, and < 0.001and <0.0001 are depicted as “*,” “**,” “***,” and “****,” respectively.

We next compared the neutralization profile of the contemporary and historic (isolated prior to 2014) HIV-1 India clade C against CAP256-VRC26.25, PGDM1400, VRC01, VRC07, and PGT121. Contemporary viruses were found to become significantly more resistant to CAP256-VRC26.25 (*P* < 0.005, Mann-Whitney U-test) and more sensitive to PGT121 (*P* < 0.01, Mann-Whitney U-test) when compared with historic viruses (collected before 2014) (Fig. 3A). While not reaching statistical significance, a trend in contemporary viruses becoming resistant to PGDM1400 and VRC01 was observed. Interestingly, VRC07 was found to demonstrate comparable neutralization of both historical and contemporary Indian clade C, which is in contrast to that observed with contemporary African clade C viruses (9).

By comparing the *env* sequences of contemporary and historical Indian clade C viruses, we found that, overall, they significantly differ in their PNLG site content in *gp120*, specifically in the V1/V2 domain and in their net charge in the V1 hypervariable region (Fig. 3B). These features may contribute to reduced sensitivity of contemporary viruses to CAP256-VRC26.25 and PGDM1400 but increased sensitivity to PGT121.

### Neutralization profiles of India and South Africa clade C viruses differ against multiple bnAb classes

**Fig 4:**
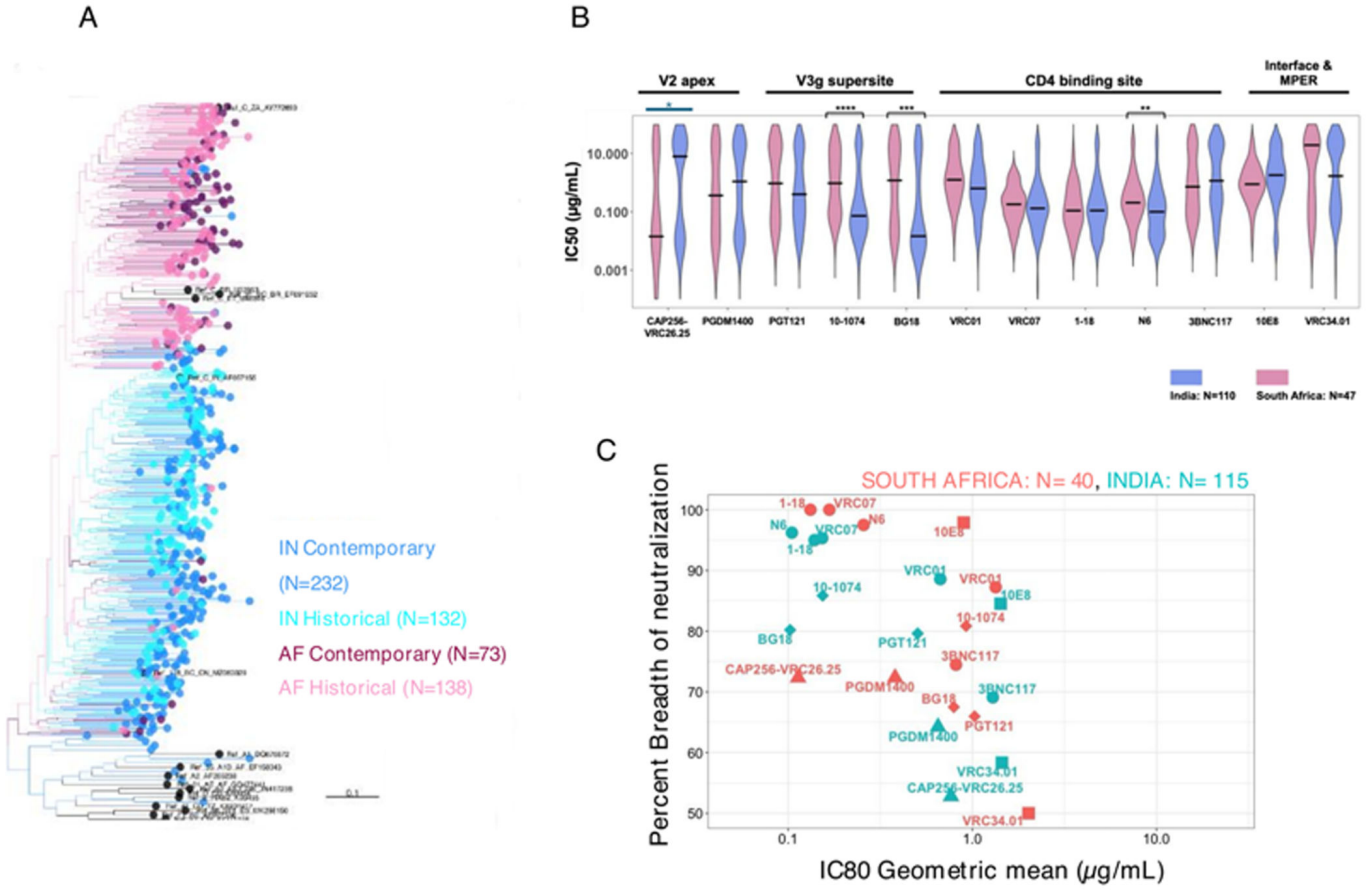
Comparison of phylogenetic and head-to-head neutralization profiles between contemporary India and South African clade C. (A) Phylogenetic relatedness of *env* genes obtained from contemporary HIV-1 clade C of India (*N* = 232) and Africa (*N* = 73) origins as well as historical India (*N* = 132) and Africa (*N* = 138) origins and 17 HIV-1 group M reference sequences. South Africa clade C *envs* comprised those obtained from the FRESH cohort (*N* = 41) and AMP placebo arm (*N* = 32). (B) Comparison of the degree of neutralization susceptibility of pseudoviruses expressing contemporary HIV-1 clade C *envs* of Indian (*N* = 115) and South African (*N* = 40; AMP placebo arm) origins to 12 best-in-class bnAbs with distinct epitope specificities on viral Env. Env expressed as a pseudovirus that showed IC50 value >25 μg/mL against a particular bnAb was considered as resistant. Statistical analysis to assess significance (*P*-values) of differences in neutralization sensitivity to a given bnAb by pseudoviruses expressing *envs* of India and South African origins was assessed by Mann-Whitney U-test. Fisher’s exact test (color coded in blue) was used to identify differences in the overall proportion of sensitive and resistant pseudoviruses. (C) Comparison of the magnitude of neutralization sensitivity of India and South Africa clade C viruses to select clinically relevant bnAbs. The neutralization breadth of each bnAb tested against India and South Africa clade C envelopes is expressed in the *y*-axis as percent neutralization at a given concentration of corresponding antibody (IgG) concentration given in *x*-axis. The values in the *x*-axis are the geometric mean of the IC80 values (μg/mL) calculated for each bnAb. Neutralization assay was carried out in duplicate replicates at least three times, and average values were used to plot the graph.

We next made a head-to-head comparison of contemporary India and South Africa HIV-1 clade C *envs (gp160*) to examine (i) their phylogenetic relatedness and (ii) their sensitivity to bnAbs. For the phylogenetic analysis, we examined 232 and 73 clade C *envs* of Indian and South African origin. Of 73 HIV-1 clade C *env* sequences of African origin, 41 were obtained from individuals enrolled in the FRESH (Females Rising through Education, Support and Health) cohort (16) and the rest (17) (9) were obtained from the placebo arm of the phase 2b HVTN 703/HPTN 081 AMP prevention trial (South Africa 19, Malawi 7, Zambia 4, and 1 each from Mozambique and Botswana) (7). As shown in Fig. 4A, we observed distinct clustering of India and South Africa viruses, consistent with our previous observation (18). This indicates that *env* genes are genetically distinct and continue to evolve independently in the two geographic regions.

We next compared neutralization sensitivity of contemporary Indian (*N* = 115) and South African clade C (*N* = 47, obtained from AMP Placebo group) *envs* against 14 bnAbs described above. We observed significant differences in their neutralization susceptibility to N6 (*P* < 0.05, Mann-Whitney U-test), 10-1074 (*P* < 0.0005, Mann-Whitney U-test), and BG18 (*P* < 0.005, Mann-Whitney U-test) (Fig. 4B), with Indian HIV-1 clade C being significantly more sensitive to these three bnAbs than African viruses. In general, for the CD4bs-directed bnAbs, we observed that except for VRC07 and 1-18, differing sensitivities were observed for VRC01, N6, and 3BNC117 between Indian and South Africa clade C viruses. India clade C viruses demonstrated increased sensitivity to the V3 glycan supersite-directed bnAbs examined. Notably, while both India and African contemporary clade C viruses were found to show poor susceptibility to CAP256-VRC26.25 compared to other bnAbs (Fig. 4B), Indian clade C viruses were found to be more resistant to CAP256-VRC26.25 compared to African viruses as determined by their mean IC50 values. When comparing the proportions of overall sensitive and resistant (IC50 >25 μg/mL) pseudoviruses, a significant difference was observed for CAP256-VRC26.25 (*P* < 0.05, Fisher’s exact test). Overall, contemporary Indian and African HIV-1 clade C vary significantly in their bnAb neutralization profiles, highlighting the divergence that can occur, even within the same clade.

### Diversity in sequence characteristics that differentiate neutralization-sensitive and resistant envelopes

**Fig 5:**
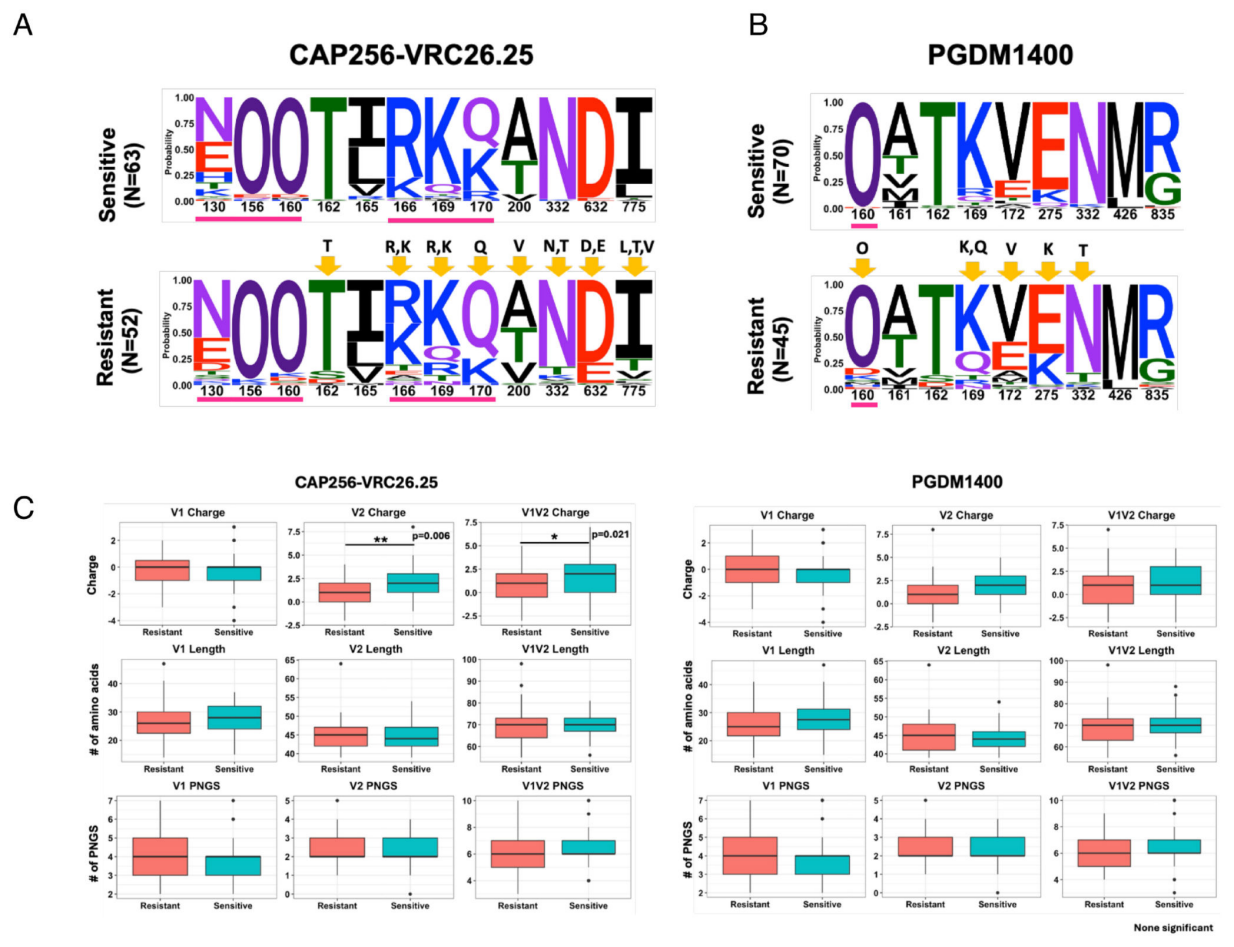
Diversity in *gp120* sequence features and contact sites polymorphism between contemporary India clade C Envs sensitive and resistant to V/1/V2 apex clinically relevant bnAbs. Frequency of contact sites associated with CAP256-VRC26.25 and PGDM1400 sensitivity was compared between CAP256-VRC26.25-sensitive and -resistant pseudoviruses (A) and PGDM1400-sensitive and -resistant viruses (B). The gp160 position (based on HXB2 numbering) of the key amino acids in the sequence logo is shown in the *x*-axis, and their relative abundance expressed as probability is in the *y*-axis. O has been used to differentiate potential N-linked glycosylated asparagine from potentially unglycosylated asparagine (N). Residues underscored in purple line are direct Ab contact sites. Residues showing statistically significant changes in abundance following a Fisher’s exact test are highlighted with yellow arrows. (C) Variable loop length, PNGs, and net charges of sensitive and resistant envelopes.

Next, we examined the amino acids in Env that form bnAb contact sites. We created sequence logos to examine the distribution of amino acids and performed statistical tests to assess the enrichment of resistance-associated signatures. Since the majority of the contemporary viruses were resistant to the V1/V2-directed bnAbs (CAP256-VRC26.25 and PGDM1400), we first examined the distribution of relevant amino acid residues within these epitopes. As shown in Fig. 5A, for CAP256-VRC26.25-resistant viruses, we saw signals at positions 160, 166, 169, 170, 200, 332, 632, and 775. We observed significant increases in the frequency of R169, Q170, T332, and decreased frequency of N160, K169, as identified by Fisher’s exact test in CAP256-VRC26.25-resistant viruses when compared with the CAP256-VRC26.25-sensitive viruses. For PGDM1400, we observed enrichment of resistance-associated residues at positions 160, 169, 172, 275, and 332 (Fig. 5B). Variation in V1/V2 loop length has been shown to modulate sensitivity to V2 apex-directed neutralizing antibodies (19–21). The CAP256-VRC26.25-resistant viruses were also found to have significantly lower net charge in the V2 region (*P* = 0.006, Mann-Whitney U-test) compared to their sensitive counterparts (Fig. 5C). Unlike South African clade C viruses, no significant differences were seen in V1 loop length of CAP256-VRC26.25-sensitive and -resistant India clade C viruses (Fig. 5C; Fig. S2), nor was there a significant difference in V2 loop length and net charge between PGDM1400-sensitive and -resistant India clade C viruses (Fig. 5C; Fig. S2). For V3-directed bnAbs, we examined residues associated with resistance to PGT121, 10-1074, and BG18. We observed higher variation/entropy within such residues in PGT121-resistant viruses followed by 10-1074- and BG18-resistant viruses (Fig. S3). For PGT121-resistant viruses, significant enrichment and reduction of key residues at position 137, 139, 140, 328, 332, and 334 were observed (Fig. S3A). In 10-1074-resistant viruses, there was significant enrichment of A137, E322, K328, Y330, T332, and N334 compared with sensitive viruses (Fig. S3A). While most contemporary viruses were potently neutralized by BG18, the few that showed resistance were significantly enriched for Y330 and N/T330 (Fig. S3A). For PGT121-resistant viruses, we observed low net charge in the V1/V2 hypervariable region compared to the PGT121-sensitive viruses (*P* = 0.04, Mann-Whitney U-test). We also observed significant differences in V4 loop length (*P* = 0.02, Mann-Whitney U-test) and net charge in the V1 loop (*P* = 0.04, Mann-Whitney U-test) in the 10-1074-resistant viruses (Fig. S3B). With respect to BG18-resistant viruses, we observed significant differences in the net charges in the V1 loop (*P* = 0.03, Mann-Whitney U-test), V4 loop length (*P* = 0.01, Mann-Whitney U-test), and PNLG content in V4 loop (*P* = 0.01, Mann-Whitney U-test) when compared with BG18-sensitive viruses (Fig. S3B). Among CD4bs-directed bnAbs examined, contemporary viruses showed least susceptibility to 3BNC117 (26.08% were found to be resistant) followed by VRC01 (23.47%), 1-18 (10.43%), VRC07 (9.56%), and N6 (7.82%) (Fig. 8A). We observed significant enrichment of E279, S280, R282, F318, V371, and E455 and significant reduction in the occurrence of N280, Y318, S365, I371, R456, G459, and G471 in viruses resistant to 3BNC117 (Fig. S4). Overall, in VRC01-resistant viruses, we observed enrichment of aspartic (D) and glutamic acid (E) residues at position 97 in C1 region of the envelope inner domain, polymorphisms at 279 and 281 positions in the loop D and enrichment of glutamic acid (E) and/or leucine (L) at 455 position, tryptophan (W) at 456 position, aspartic acid (D) at asparagine (N) at the positions 455, 456, and 474 positions in the β23/loop-β24/V5 region of the viral Env protein. Similarly, in 3BNC117-resistant viruses, we observed significant polymorphism at positions 279, 280, 282, 318, 371, 455, 459, and 471 on viral Env protein that is associated with modulation of sensitivity to 3BNC117 (https://www.hiv.lanl.gov/components/sequence/HIV/neutralization/main.comp). Interestingly, we observed enrichment of several amino acid residues at positions 279, 280, 281, 355, 365, 456, 459, 463, and 471 in the N6-resistant viruses (Fig. S4) around the CD4bs region. Significant differences observed between the variable region of viruses sensitive and resistant to VRC01, VRC07, and N6 are shown in Fig. S5.

**Fig 6:**
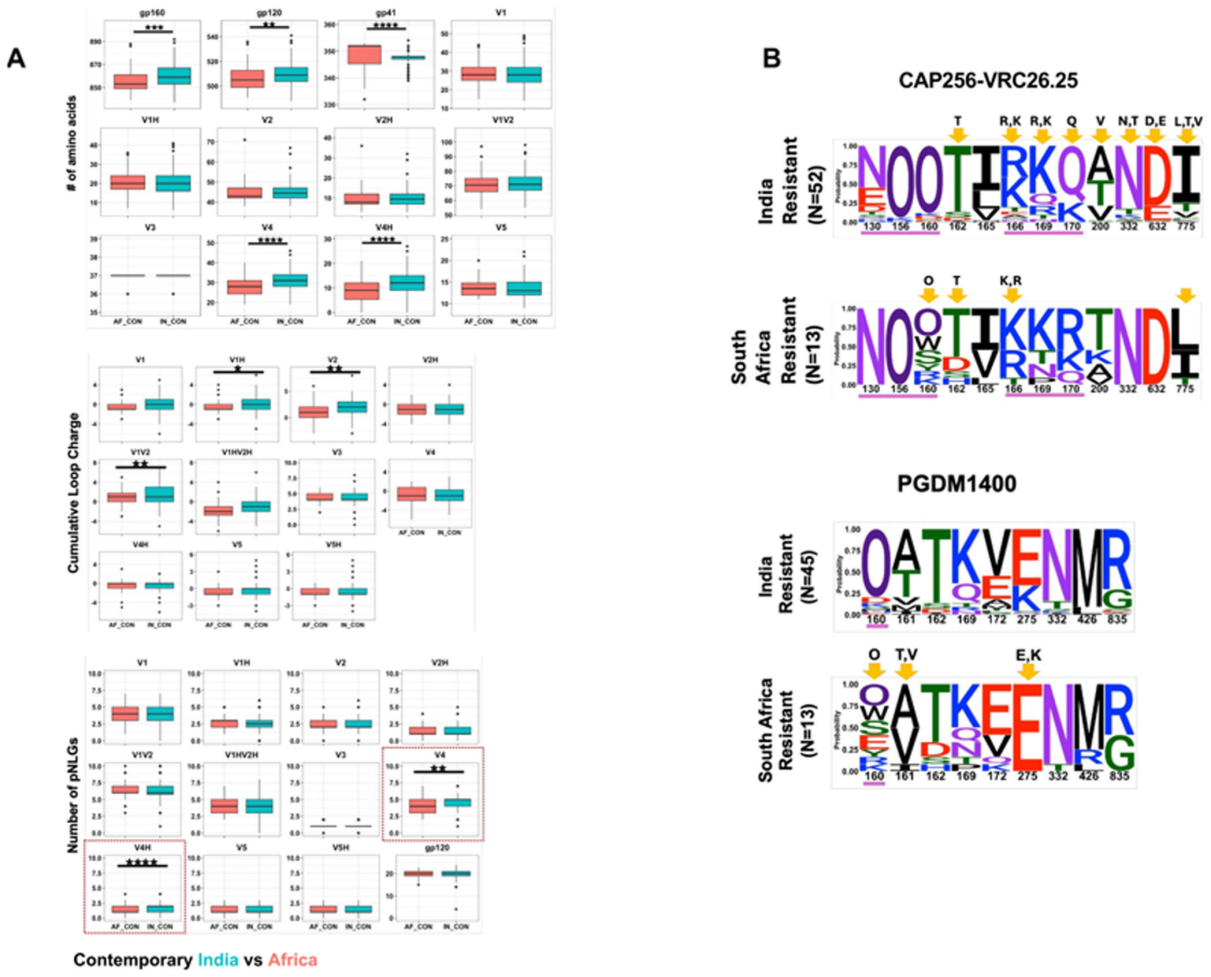
Comparison of *env* sequence features of contemporary India and South Africa clade C viruses. (A) The amino acid sequences of complete *envs* (*gp120 and gp41*) of India and South Africa contemporary HIV-1 clade C were analyzed to compare their average variable loop lengths, PNLGs, and net charges in *gp120* as well as the length of gp41. These are analyzed using the “variable region characteristics” tool available at the Los Alamos HIV database (https://www.hiv.lanl.gov/content/sequence/VAR_REG_CHAR/index.html) and N-Glycosite (https://www.hiv.lanl.gov/content/sequence/GLYCOSITE/glycosite.html). (B) Comparison of keyamino acid residues on India and South Africa clade C *envs* that are linked with CAP256-VRC26.25 and PGDM1400 resistance is shown in sequence logos. The statistically significant enrichment of key residues for viruses sensitive and resistant to CAP256-VRC26.25 and PGDM1400 is shown on the *y*-axis. O has been used to differentiate potential N-linked glycosylated asparagine from potentially unglycosylated asparagine (N). Amino acid residues underscored in purple line are direct Ab contact sites for respective bnAbs. Residues showing statistically significant changes in abundance following a Fisher’s exact test are highlighted with yellow arrows.

When we compared the sequence features of the contemporary HIV-1 clade C *env* of Indian and South African origins, we observed a significant difference in their *gp160* (both *gp120* loop and *gp41*) lengths (Fig. 6A). In particular, we found a significant difference in the V4 loop length between the contemporary viruses from these two geographically distinct regions, with longer loops observed with Indian contemporary viruses. Moreover, contemporary India and Africa clade C *envs* also significantly differed in their V1/V2 net charge and PNLGs in the V4 loop (Fig. 6A). We compared the *env* sequences of India and Africa viruses that showed resistance to V1/V2-directed bnAbs. For CAP256-VRC26.25- and PGDM1400-resistant viruses, we found differences at sites 160, 166, 169, and 170 (Fig. 6B). Interestingly, except for differences in net charge in the V2 loop between PGDM1400-resistant viruses of India and South Africa clade C, no differences in loop lengths and PNGs were observed between CAP256-VRC26.25- and PGDM1400-resistant India and South Africa clade C viruses suggestive of similar mechanisms of resistance across the two regions (Fig. S6). We also observed differences in frequencies of contact residues targeted by CD4bs-(N6) and V3 glycan-directed (PGT121, 10-1074, BG18) bnAbs between India and South Africa clade C viruses (Fig. S7), which may explain the differences in their sensitivity (Fig. 4B). While the above analysis

was carried out using the *env* sequences, which were expressed and tested against the selected bnAbs as pseudoviruses, we analyzed additional contemporary clade C *env* sequences (not used for preparing pseudoviruses) isolated from nine geographically distinct regions of India (as described above) and from South Africa (sourced from FRESH cohort). While both data sets displayed enrichment of resistant signatures for CAP256-VRC26.25 bnAb at positions 165, 166, and 169, statistically significantly different enrichment of resistant signature was observed at residue position 166 (Fig. S8). Analysis of PGDM1400 contact residues indicated a trend of differential abundance at position 160 with significant differences at positions 130 and 161 as well as 211. N332, the target site for PGT121, 10-1074, and BG18 bnAbs, was more conserved in Indian sequences compared to those from South Africa (Fig. S8). Overall, our data indicated that the differential sensitivity of India and Africa clade C contemporary viruses to various bnAb classes is associated with distinct sequence features including those in bnAb contact residues.

### Viruses resistant to V1/V2-directed antibodies remain well neutralized by CD4bs-directed antibodies

**Fig 7:**
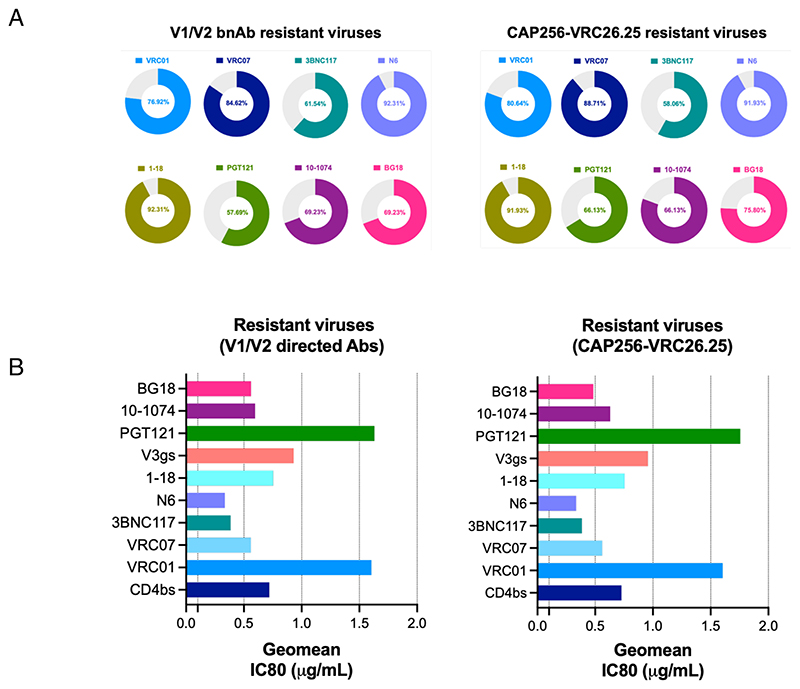
Neutralization of V1/V2 apex bnAb-resistant contemporary Indian clade C viruses by CD4bs- and V3 glycan supersite-directed bnAbs. (A) Sensitivity of pseudoviruses expressing contemporary Indian clade C *envs* which were fully resistant to all V1/V2-directed bnAbs to CD4bs (VRC01, VRC07, 3BNC117, N6, and 1-18) and V3 glycan supersite (PGT121, 10-1074, and BG18). Left panel shows percent neutralization of pseudoviruses that were resistant to all V1/V2-directed bnAbs tested (CAP256-VRC26.25, PGDM1400, PG9) (*N* = 26) by CD4bs- and V3 glycan-directed bnAbs. The right panel shows the same but only to pseudoviruses resistant to CAP256-VRC26.25-resistant envelopes (*N* = 62). Percent neutralization breadth conferred by CD4bs- and V3 glycan-directed bnAbs was calculated by the number of resistant viruses that showed IC80 values <25 μg/mL. (B) Magnitude of neutralization of V1/V2-directed bnAb-resistant pseudoviruses conferred by each of the CD4bs- and V3 glycan-directed bnAbs. The magnitude of virus neutralization equivalent to potency was measured as the lowest geometric mean titer conferred by each bnAb IgG (μg/mL) that demonstrated 80% neutralization of pseudovirus. Neutralization assay was carried out in duplicate replicates at least three times, and average values were used to plot the graph.

We next examined the ability of other bnAbs to neutralize V1/V2-directed bnAb-resistant viruses. As shown in Fig. 7, CAP256-VRC26.25-resistant viruses were found to be best neutralized by CD4bs-directed bnAbs (82.25% breadth) over the V3-directed bnAbs (74.19%). Among the CD4bs-directed bnAbs, N6 and 1-18 demonstrated best breadth (91.93%), and 3BNC117 was found to be least broad among all (58.06%). As for V3-directed bnAbs, CAP256-VRC26.25-resistant viruses were found to be best neutralized by 10-1074 (80.64%), followed by BG18 (75.80%) and PGT121 (66.13%). When we analyzed viruses that demonstrated complete resistance to all the V1/V2 apex-directed bnAbs, we again found that compared to V3-directed bnAbs (65.38% breadth), they are best neutralized by CD4bs-directed bnAbs (81.53%) with both N6 and 1-18 demonstrating maximum breadth (92.30% breadth in both). However, N6 was found to be more potent with IC50 of 0.35 μg/mL over 1-18 with IC50 of 1.01 μg/mL. Our data indicate that while contemporary Indian clade C viruses showed poor susceptibility to V1/V2-directed bnAbs, they remain broadly sensitive to lead CD4bs- and V3-specific bnAbs.

### VRC01- and 3BNC117-resistant viruses are neutralized by second-generation CD4bs bnAbs but with reduced potency

**Fig 8:**
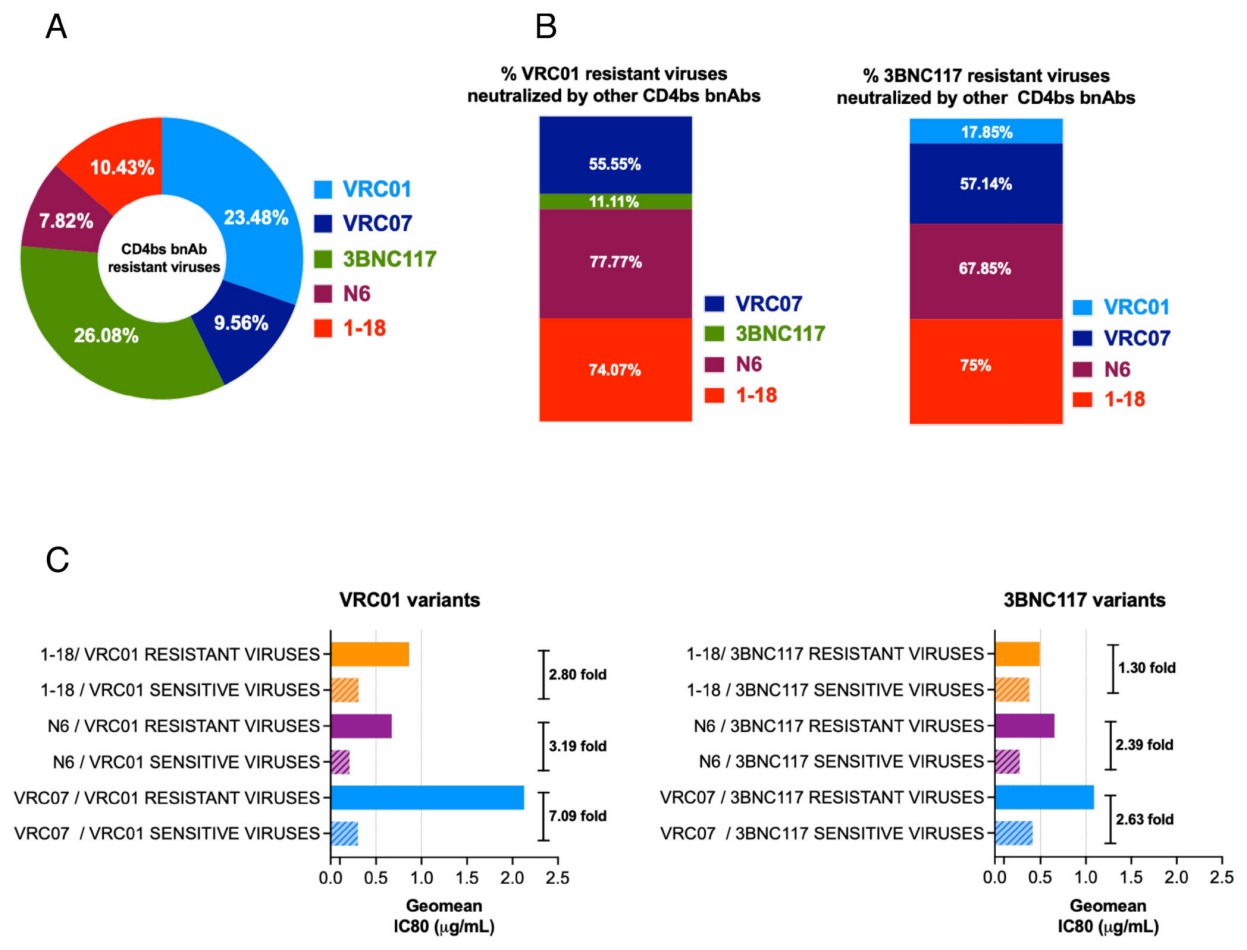
Neutralization efficiency of VRC01- and 3BNC17_resistant contemporary viruses by second-generation CD4bs-directed bnAbs. (A) Proportion of contemporary viruses (*N* = 115) that were found to be resistant to first-(VRC01 and 3BNC117) and second-generation (VRC07, N6, 1-18) CD4bs-directed bnAbs. Pseudoviruses with a neutralization score of IC80 >25 μg/mL to respective bnAbs were considered resistant. (B) Proportion of VRC01- and 3bnc117-resistant contemporary pseudoviruses that demonstrated sensitivity to second-generation CD4bs bnAbs (VRC07, N6, 1-18). Note that both VRC01- and 3BNC117-resistant viruses were least neutralized by 3BNC117 (11.11%) and VRC01 (17.85%) compared to VRC07, N6, and 1-18, indicating that the viruses resistant to both of them lack common key residues that are essential for both VRC01 and 3BNC117 for comprehensive neutralization. All the second-generation CD4bs bnAbs showed better neutralization (over 50%), with 1-18 demonstrating most (>74%) of VRC01- and 3BNC117-resistant viruses. (C) Comparison of the magnitude of neutralization of VRC01- and 3BNC117-sensitive and -resistant viruses by second-generation CD4bs bnAbs. Left panel shows the differences in the magnitudes of neutralization of VRC01-sensitive and -resistant viruses by all three CD4bs bnAbs (VRC07, N6, 1-18) and the right panel shows the same with 3BNC117-sensitive and -resistant viruses. The fold difference in magnitude of neutralization was obtained by calculating the average (GMT) of IC80 (μg/mL) for each paired set. GraphPad Prism was used to plot all the graphs.

While CD4bs-directed antibodies were found to demonstrate best neutralization coverage of the contemporary viruses, we next examined whether resistance to CD4bs bnAbs VRC01 and 3BNC117 conferred decreased sensitivity to second-generation CD4bs bnAbs. As shown in Fig. 8A, among all the CD4bs bnAbs tested, contemporary Indian clade C showed greater resistance to VRC01 and 3BNC117 (23.48% and 26.08%, respectively) compared to those that showed resistance to VRC07 (9.56%), N6 (7.82%), and 1-18 (10.43%). We next examined the extent of neutralization of VRC01- and 3BNC117-resistant contemporary viruses by other CD4bs bnAb classes. We observed that N6 neutralized (77.77%) most of the VRC01-resistant viruses, while 1-18 could neutralize (75%) most of the 3BNC117-resistant contemporary viruses (Fig. 8B). Interestingly, when compared with VRC01- and 3BNC117-sensitive viruses, VRC07, N6, and 1-18 were found to neutralize VRC01- and 3BNC117-resistant viruses with reduced potency by over twofold (Fig. 8C). The reduced potencies could likely be due to the enrichment of resistance-associated amino acid residues observed when we compared the VRC01- and 3BNC117-sensitive and -resistant viruses. Overall, we found that in addition to clinically relevant V1/V2 bnAb-resistant viruses, the second-generation CD4bs bnAbs such as VRC07, N6, and 1-18 are capable of neutralizing viruses that are resistant to first-generation CD4bs bnAbs VRC01 and 3BNC117.

### Combination of BG18, N6, and PGDM1400 is predicted to provide optimal neutralization coverage of HIV-1 India clade C, including difficult-to-neutralize viruses

**Fig 9:**
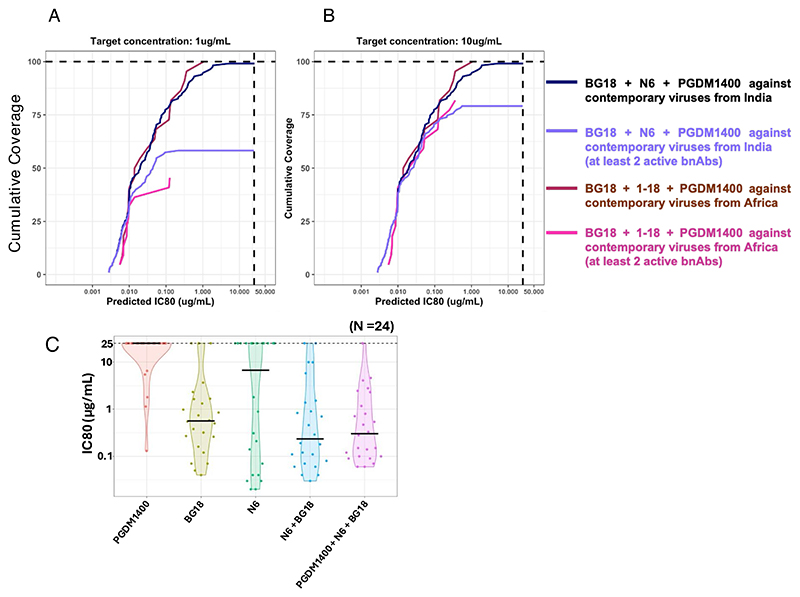
Predictive neutralization coverage of contemporary India clade C viruses by clinically relevant bnAbs. Cumulative neutralization coverage of pseudo viruses carrying contemporary HIV-1 clade C *envs* by bnAb combination was assessed using the CombiNAber tool using the Bliss-Hill statistical model. (https://www.hiv.lanl.gov/content/sequence/COMBINABER/combinaber.html). CombiNAber analysis of 115 contemporary viruses from India against BG18 + N6 + PGDM1400 and 45 contemporary viruses from Africa against BG18 + 1-18 + PGDM1400, as well as the same combinations with at least two active bnAbs, have been plotted for target bnAb concentrations of 1 μg/mL (A) and 10 μg/mL (B), respectively. Predicted IC80 (μg/mL) combinations have been plotted on the *x*-axis, while the cumulative breadth of the viruses has been depicted on the *y*-axis. (C) Pseudoviruses expressing 24 contemporary difficult-to-neutralize *envs* were assessed for their degree of susceptibility to PGDM1400, N6, BG18, a combination of N6 and BG18, and a combination of PGDM1400, N6, and BG18. We used single bnAb at starting concentrations of 25 μg/mL with subsequent fivefold dilutions up to 0.00032 μg/mL, along with two combinations of 12.5 μg/mL each of BG18 and N6, and three combinations of 8.33 μg/mL each of BG18, N6, and PGDM1400 Abs. IC80 refers to the IgG concentrations (μg/mL) at which pseudoviruses demonstrated 80% neutralization, respectively. Pseudoviruses that were not neutralized up to 25 μg/mL of IgG were considered as resistant viruses.

Toward identifying the most optimal combination of bnAbs capable of comprehensively neutralizing contemporary clade C viruses, we included bnAbs that demonstrated neutralization breadth >50% with IC80 of <25 μg/mL. Also, in order to perform a head-to-head comparison, we assessed the extent of neutralization coverage of the contemporary clade C viruses from Africa (*N* = 40) by the same set of bnAbs. The CombiNAber analysis (https://www.hiv.lanl.gov/content/sequence/COMBINABER/combinaber.html) was carried out for both sets of viruses (of India and Africa origins) at the target concentration of 1 μg and 10 μg/mL, respectively, using the Bliss-Hill model. At 10 μg/mL, Indian contemporary viruses were observed to be most effectively neutralized by N6 and BG18 (Fig. 9A). Both of these provided 91% and 72% coverage at the target concentration with potency (IC80) of 0.44 and 0.30 μg/mL, respectively. Also, 1-18 and 10-1074 were the next best two CD4bs- and V3-directed bnAbs with breadth of 83 and 80 and potency (IC80) of 0.59 and 0.63 μg/mL, respectively. With respect to the contemporary clade C viruses from Africa, N6 was observed to be the most effective bnAb with 90% coverage and potency (IC80) of 0.51 μg/mL. PGDM1400 and BG18 were comparably the next most effective bnAbs with neutralization breadth of 57.7% and 56.6% and potency (IC80) of 1.59 and 1.54 μg/mL, respectively. When we assessed three bnAb combination predictions, BG18 + N6 + PGDM1400 appear to provide the best neutralization coverage of Indian contemporary viruses with 99.13% breadth with IC80 predicted to be at 0.03 μg/mL.

However, the coverage drops to 79% when considering at least two active bnAbs. For the HIV-1 clade C from South Africa, the combination of BG18 + PGDM1400 + 1-18 appears to be the best combination with 100% coverage at 0.03 μg/mL IC80. The coverage drops to a mere 81.81% when considering at least two active bnAbs. At 1 μg/mL, Indian contemporary clade C viruses appeared to be most effectively neutralized by BG18 and N6 (Fig. 9B) as above. However, they showed 64 and 66% neutralization coverage at the target concentration with potency (IC80) of 0.30 and 0.44 μg/mL, respectively. Similarly, as with the 10 μg/mL concentration, 1-18 and 10-1074 were the next best bnAbs found with predicted neutralization coverage of 64% and 66% and potency (IC80) of 0.59 and 0.63 μg/mL, respectively. For the contemporary clade C viruses from South Africa, the most effective single mAbs were found to be 1-18 and BG18. These two bnAbs were observed to provide 65 and 43% coverage, respectively, with potency of 0.53 and 1.54 μg/mL, respectively. When we assessed neutralization coverage by three-antibody combination, BG18 + N6 + PGDM1400 was observed to provide 93.91% coverage of Indian contemporary clade C viruses at IC80 of 0.03 μg/mL. This neutralization coverage, however, drops to 58% when at least two active bnAbs were considered. Conversely, we observed BG18 + PGDM1400 + 1-18 combination to provide 95.45% coverage of African contemporary viruses at IC80 of 0.03 μg/mL which drops significantly to 45.45% when considering at least two active bnAbs. Overall, our predictive data indicate that no combination could provide 100% coverage for the clade C viruses from India and African origin at any of the considered target concentrations. The data further indicated that while a combination of V3-, V2 apex-, and CD4bs-directed bnAbs was effective across both regions, the clade C viruses from India and Africa are distinctly sensitive to different bnAbs of clinical relevance.

We further carried out pseudovirus neutralization assays to examine the extent to which the combination of N6, BG18, and PGDM1400 (which showed maximal neutralization coverage by predictive analysis) could neutralize the difficult-to-neutralize viruses (*n* = 24) that we identified in our current study. As shown in Fig. 9B(and Table 3), we found that 23 of 24 (95.83%) of these viruses were neutralized by the combination of these three bnAbs with IC80 ranging from 0.06 to 4.6 μg/mL, whereas the combination of N6 and BG18 could neutralize 22 of 24 (91.66%) of the viruses. Among all the difficult-to-neutralize viruses, TSG21N01N017_C18 *env* was found to be broadly resistant to the majority of the bnAbs tested, including N6 and BG18 (Table 3). Overall, both our predictive and experimental data indicate that the combination of N6, BG18, and PGDM1400 is likely to provide maximal neutralization coverage of the contemporary HIV-1 India clade C, including those that are difficult to neutralize.

## Discussion

While HIV-1 clade C is the major globally circulating form, evolutionary patterns may vary across different geographical regions representing ethnically diversified populations that may contribute to differential susceptibility to class-specific bnAbs. For example, there has been a significant association between HIV evolution at the population level and increased resistance to serum and bnAb-mediated neutralization observed in HIV-1 clade B-infected individuals (11–13). Moreover, intra-clade diversity has been predicted to have better neutralization advantage in geographical regions with lower viral diversity compared to regions with substantial intra-clade diversities (22). Therefore, it is unclear whether the same combination of select bnAbs would stand fit to comprehensively provide neutralization coverage of the globally circulating and evolving HIV at the population level. It is therefore important to understand whether globally evolving HIV at a geographically and ethnically distinct population level can influence antigenic properties. Little information is available for contemporary HIV-1 clade C viruses predominantly circulating across India.

In the present study, we examined how *env* sequence diversity of contemporary HIV-1 Indian clade C (isolated between 2020 and 2023) differentiates them from historical viruses as well as contemporary HIV-1 clade C of South African origin. To encompass contemporary HIV-1 of Indian origin at the population level, we obtained samples as sources of HIV from nine geographically distinct origins representing different risk groups. Although region-specific numbers of viruses were moderate, perhaps accounting for the fact that we saw no region-specific clustering, to the best of our knowledge, this is the first such study of genetic and neutralization profiles of contemporary viruses from geographically distinct regions in India. A larger sample size of region-specific circulating forms would provide more precise phylogenetic details.

Although the Indian contemporary clade C *envs* continue to cluster genetically with historical viruses, we found a significant drift in the degree of their sensitivity to CAP256-VRC26.25 and PGDM1400, the two clinically relevant bnAbs that target the V1/V2 apex region of the viral Env protein. Over 45% and 40% of the contemporary viruses were found to be resistant to CAP256-VRC26.25 and PGDM1400, respectively. Our observation is consistent with our earlier study (10) and that of South African clade C viruses (9). Conversely, the contemporary India viruses showed increased sensitivity to PGT121, which is in contrast to previous observations in South African clade C viruses (9). These differences could be due to increased predicted glycosylation in *gp120*, particularly in V1/V2, as previously described (9, 23, 24), and possibly also due to the differences in net V1 charges as observed in our study. The resistance to CAP256-VRC26.25 and PGDM1400 is also likely due to enrichment of resistance-associated amino acid residues in the key contact sites on viral envelope protein, such as enrichment of K166, Q169, and/or K169 residues in CAP256-VRC26.25-resistant viruses and D160, Q169, and K275 in PGDM1400-resistant viruses.

Mkhize et al. (9) recently also reported a trend in decreasing sensitivity of Africa clade C (obtained from the placebo arm of the AMP trial participants) to VRC01 and VRC07, an observation that was not noted with Indian clade C viruses tested in this study. These observations, along with other *env* sequence features such as loop length, glycosylation, and net charges that differentiated contemporary India and Africa HIV-1 clade C, clearly indicate that they continue to evolve independently and distinctly at the population level.

A notable observation made was that the second-generation CD4bs bnAbs (N6, 1-18, and VRC07) were able to neutralize contemporary Indian clade C viruses with significantly better breadth and potency compared to the first-generation CD4bs bnAbs (VRC01 and 3BNC117). They were also found to neutralize the majority of the contemporary viruses that showed resistance to the V1/V2 apex-directed bnAbs (CAP256-VRC26.25 and PGDM1400) and VRC01 and 3BNC117. Such observations indicate that in comparison to V1/V2-directed bnAbs, the key contact sites and epitopes for N6, 1-18, and VRC07 are evolutionarily preserved. The poor neutralization breadth conferred by VRC01 and 3BNC117 could possibly be because of the substitutions of amino acid residues resulting from selection pressure during the course of natural infection at one or more of their key contact sites that were reported to be essential for their ability to neutralize efficiently (25–27). Interestingly, the second-generation CD4bs bnAbs (N6, 1-18, and VRC07) were found to neutralize the VRC01- and 3BNC117-resistant viruses with over twofold lower potency than what was observed with their corresponding sensitive viruses. This could possibly be due to the following reasons observed with a few VRC07-, N6-, and 1-18-resistant viruses: (i) increased net charge in V1/V2 hypervariable regions (VRC07), differences in PNGS content in V1/V2 region, and increased V1/V2 net charge in V/1V2 (N6) and/or (ii) enrichment of resistance-associated residues.

Combination of best-in-class bnAbs with distinct specificities has been reported to improve the optimal neutralization coverage of the HIV-1 diversity both by prediction and real-world application in experimental trials (28–32). Emergence of HIV-1 clade C variants that showed broad resistance to major clinically relevant bnAbs was an interesting observation to note. Although few were identified in this study, their presence in early infected individuals may imply that such resistant viruses can transmit and establish infection. Moreover, more such broadly resistant viruses are likely to evolve over time at the population level. It is therefore important to identify bnAbs that can be included in the antibody cocktail that can suitably compensate for the inability of the existing best-in-class bnAbs to neutralize such viruses. Identification of viruses that are broadly resistant to existing best-in-class bnAbs also provides an opportunity to isolate a new class of antibodies with new target specificities that are capable of neutralizing evolving viruses that are broadly resistant to the existing bnAbs. Based on individual virus neutralization data, we predicted that BG18 + N6 + PGDM1400 would provide maximal coverage. This was also validated by neutralization assays.

One of the interesting observations made in this study is the identification of mutations in the *pol* gene associated with drug resistance in isolates obtained from over 10% ART-naïve donors (Table S1). This indicates the ability of the establishment of infection by drug-resistant viruses (17, 33), which can potentially minimize the efficacy of antiretroviral therapy post-exposure. Such observation further justifies the importance of using next-generation bnAbs as a prevention strategy. While all the samples studied here were collected between 2019 and 2023, out of the 232 unique contemporary sequences assessed, 139 were estimated to have been infected post 2019. The remaining 93 sequences were obtained from chronically infected individuals and therefore may not be entirely contemporaneous. However, among the pseudoviruses directly compared between India and Africa, 66% from India have been isolated from early disease stage, while all from Africa were isolated from early disease stages. Only 6 of 115 (5.1%) virologically suppressed study participants were initiated with ART prior to 2016. This is a limitation of the present study. We examined the neutralization properties of the cross-sectionally collected HIV+ samples and it will be useful to periodically monitor how HIV evolution over time influences the efficacy of the clinically relevant bnAbs. Moreover, several bnAbs that are under clinical development were isolated a while ago, and emerging reports, including our present study, indicate several of them may have reducing efficacies against the circulating viruses in the populations (9, 14). Therefore, the need for periodic assessment of sequence and neutralization profiles of the regionally relevant contemporary HIV-1 forms against engineered optimized clinically relevant bnAbs is required for prioritizing and development of effective bnAbs as products for prevention.

## Materials and Methods

### Study participants

,A total of 232 study participants were recruited from nine different geographical sites as indicated in Table 1. Clinical parameter data such as CD4 counts, viral load, and antiretroviral therapy status were obtained for each study participant.

### Additional sequences used from other cohorts

HIV-1 India clade C historical sequences (*N* = 132) included in the present analysis were reported through our earlier work (10). These were sampled prior to the year 2014 from treatment-naive early seroconverts and chronically HIV-infected individuals with high plasma viremia (*N* = 126), including six acutely infected individuals (34). African contemporary sequences assessed have been included from the FRESH cohort (*N* = 41) and the placebo arm of the phase 2b HVTN 703/HPTN 081 AMP prevention trial (*N* = 32). All of these sequences have been sampled between the years 2013 and 2020 from acutely infected individuals.

### Plasmids, antibodies, and cells

Plasmids encoding full-length codon-optimized *gp160* of Indian origin synthesized at GenScript Inc. were used for preparing pseudoviruses. Plasmids encoding HIV-1 clade C *env* genes of South African origin from the AMP placebo arm reported earlier (9) were used to prepare pseudoviruses for the neutralization assay. pSG3ΔEnv was obtained from the NIH AIDS Reagent and Reference Program. Plasmids encoding heavy and light chain immunoglobulins of CAP256-VRC26.25 were provided by Prof. Lynn Morris, and ones with VRC01, VRC07, N6, 1-18, PGDM1400, 3BNC117, BG18, 10-1074, 10E8, and VRC34.1 were provided by the IAVI Neutralizing Antibody Center. HEK 293T, TZM-bl, were obtained from the American Type Culture Collection (ATPC) and GHOST-Hi5, GHOST-CXCR4 cells were obtained from the NIH AIDS Reagents & Reference Program, respectively. GHOST-CCR8 cells were kindly provided by Paul Clapham. Expi293 cells were purchased from Thermo Inc.

### Isolation of viral and genomic DNA and cDNA synthesis

Viral RNA was isolated from plasma using the High Pure viral RNA kit (Roche) as per manufacturer’s instruction as described earlier (35). Genomic DNA was isolated from peripheral blood mononuclear cells using QIAmp blood DNA mini kit (Qiagen) as per the manufacturer’s instructions and as described earlier (35). Plasma isolated RNA was primed with EnvR1 oligo (5′-GCACTCAAGGCAAGCTTTATTGAGGCT-3′) proximal to 3’ end of the HIV RNA genome (HXB2: 9605–9632) and Aenvseq4 (5’-CAAGCTTGTGTAATGGCTG AGG-3’) binding downstream of the *pol* gene (HXB2: 6817–6838). Synthesis of cDNA was performed using the Superscript III first strand synthesis kit (Invitrogen) following the protocol provided by the manufacturer.

### Amplification of full-length *gp160* and *pol*

Full-length *env* (*gp160*) genes were PCR amplified from HIV+ plasma samples with slight modification as described previously (35). *Rev-env gp160* cassette was amplified from the cDNA product using La Taq high fidelity DNA polymerase in the 1st round (Takara Bio Inc.) and PrimeSTAR GXL high fidelity DNA polymerase (Takara Bio Inc.) in the second round. The primers used for the 1st round were EnvF1: 5′-AGARGAYAGATGGAA CAAGCCCCAG-3′ (HXB2: 5550–5574) and EnvRP2: 5′-GTGTGTAGTTCTGCCAATCAGGGAA -3′ (HXB2: 9157–9181) while for the second round were Env IF: 5′-CACCGGCTTAGGCATC TCCTATGGCAGGAAGAA-3′ (HXB2: 5950–5982) and EnvIR: 5′-TATCGGTACCAGTCTTGAGAC GCTGCTCCTACTC-3′ (HXB2: 8882–8915). PCR conditions followed for both rounds were initial denaturation of 94°C for 2 min followed by 15 cycles of 94°C for 10 s, 60°C for 30 s, 68°C for 3 min, 20 cycles of 94°C for 10 s, 55°C for 30 s, and 68°C for 3 min with final extension of 68°C for 10 min. The *gp160* amplicons were purified and subsequently subjected to short read (Illumina) and long read (Oxford nanopore) deep sequencing to obtain dominant sequences as described below,. The selected sequences were then subjected to codon optimization, synthesized, and cloned into the pcDNA3.1 expression vector. Few *env* clones (Table 1) were cloned in-house in pcDNA3.1/V5-His-TOPO (Invitrogen Inc.) vector as described before (35). The primers used for the 1st round toward pol amplification were Pro5F: 5′-AGAAATTGCAGGGCCCCTAGGAA-3′ (HXB2: 1996–2018) and PolR1: 5′-GGTACCCCATAATAGACTGTRACCCACAA-3′ (HXB2: 6324–6352) while for the second round were Pro3F: 5′-AGANCAGAGCCAACAGCCCCACCA-3′ (HXB2: 2143–2166) and PolR2: 5′-CTCTCATTGCCACTGTCTTCTGCTC-3′ (HXB2: 6207–6231). PCR conditions followed in both rounds were initial denaturation of 94°C for 2 min followed by 15 cycles of 94°C for 10 s, 65°C for 30 s, 68°C for 3 min, 20 cycles of 94°C for 10 s, 55°C for 30 s, and 68°C for 3 min with final extension of 68°C for 10 min.

### Next-generation deep sequencing and construction of *env* sequences

Env amplicons were sequenced using both long-read Oxford Nanopore (ON) and short-read Illumina platforms. Next-generation sequencing was performed for 5’ fragments using the Illumina platform, while 3’ fragments were sequenced using both Illumina and Oxford Nanopore platforms. The raw data obtained from the nanopore sequencing were converted to Fastq files using Guppy basecaller (v.6.3.7). Raw reads were further filtered for quality and read length using Prowler (Flags: -l 1500 -q 12 -c “LT” -g “F1” -m “S”) (36). The reads were aligned to the HIV-1 subtype C reference sequence (GenBank ID: AF067155.1) (37) using Minimap2 (38, 39) and processed for read sorting and filtration with samtools (40). Reads encompassing the entire gene were extracted from the binary alignment maps using Picard tools (https://broadinstitute.github.io/picard/). Reads were further clustered and corrected using isONclust and isONcorrect,respectively, into quasispecies clusters (41, 42). Reads within the same quasispecies clusters were merged together into consensus sequences for each quasispecies cluster using iVar (43). Quasispecies thus constructed were further corrected for frameshift errors resulting with the help of Illumina reads using Pilon (44).

### Preparation of Env pseudoviruses

Pseudotyped viruses were prepared as described previously (45). Briefly, 293T cells were co-transfected by plasmid DNA encoding *gp160* and pSG3ΔEnv plasmid (having a premature stop codon at the beginning of *env*) into 293T cells in six-well tissue culture plates using FuGENE6 transfection reagent kit (Promega Inc.). Cell culture supernatants containing pseudotyped viruses were harvested at 48 h post-transfection and subsequently stored at −80°C until use. The virus infectivity was measured using TZM-bl reporter cells by addition of pseudoviruses containing DEAE-dextran (25 μg/mL) in 96-well microtiter plates, and the viral titers were determined by measuring the luciferase activity using Britelite luciferase substrate (PerkinElmer Inc.) with a Victor X2 luminometer (PerkinElmer Inc.).

### Coreceptor usage

Coreceptor preference of contemporary envelopes was examined by cell-cell fusion assay as described before (46). Briefly, 293T cells expressing individual *env* were mixed with GHOST-Hi5, GHOST-CXCR4, and GHOST-CCR8 post 24 h of transfection and further incubated for an additional day at 37°C in a CO_2_ incubator. Syncytia forming giant cells were identified by staining with chilled methanol containing 1% methylene blue and 0.25% basic fuchsin. 293T cells expressing 16055-2.3 for GHOST-Hi5 (34), NARI-VB105 (46) for GHOST-CXCR4, and NARI-VB52 for GHOST-CCR8 (47) were used as positive controls.

### Pseudovirus neutralization assay

Neutralization assays were carried out using TZM-bl cells as described before (45). Briefly, Env-pseudotyped viruses were pre-incubated in 96-well tissue culture plates with various concentrations of bnAbs (IgG) for an hour at 37°C in a CO_2_ incubator under humidified conditions. Subsequently, 1 × 10^4^ TZM-bl cells were added to the mixture in the presence of 25 μg/mL DEAE-dextran (Sigma, Inc.). The plates were further incubated for 48 h. The degree of virus neutralization was assessed by measuring reduction in relativelumines cence units in a luminometer (Victor X2; PerkinElmer Inc.). The IC50 and IC80 values were calculated using R using the DRC statistical package (analysis of dose response curves [48]).

### ARV resistance mutation prediction

Illumina FASTQ reads were filtered for quality (>Q30) using Trimmomatic (v.0.39). All the reads were aligned to the HXB2 genome using bwa-mem (v.0.7.17-r1188). BAM files were filtered for quality using samtools. Variant calling was performed for the *pol* gene using the iVar pipeline. Drug resistance mutation prediction was then performed for the variants obtained using the Stanford drug resistance database HIVdB (https://hivdb.stanford.edu/hivdb/by-patterns/). Resistance patterns were recorded only for variants withfrequency greater than 10%.

### Phylogenetic analysis

Phylogenetic trees were generated for 249 HIV-1 envelope amino acid sequences, which included 232 contemporary sequences from India and 17 HIV-1 group M subtype reference sequences, and for 594 HIV-1 envelope amino acid sequences, which included 232 contemporary and 132 historical sequences from India and 74 contemporary and 138 historical sequences from Africa, along with 17 HIV-1 group M subtype reference sequences. These sequence data sets were aligned using MAFFT, and the alignment was manually curated in BioEdit v.7.2.5. The tree was constructed with IQ-TREE under the HIVb model (49, 50) with estimated γ parameters and number of invariable sites. The robustness of the tree topology was further assessed by SH-aLRT as well as 1,000 ultrafast bootstrap replicates implemented in IQ-TREE as described earlier (18).

### Variable region characteristics and prediction of pNLG

Variable region characteristics such as loop length, charge, and number of pNLG sites were assessed for all envelope sequences using the “variable characteristics tool” hosted at the Los Alamos National Laboratory HIV database (LANL-HIVDB, https://www.hiv.lanl.gov/content/sequence/VAR_REG_CHAR/index.html). Potential N-linkedglycosylation sites prediction was performed with the tool N-Glycosite at LANL-HIVDB (https://www.hiv.lanl.gov/content/sequence/GLYCOSITE/glycosite.html).

### bnAb contact site assessment

For bnAbs CAP256-VRC26.25, PGDM1400, PGT145 (V2 apex directed), PGT121, BG18, 10-1074 (V3g supersite directed), VRC01, VRC07, 1-18, N6, 3BNC117 (CD4 binding site), and 10E8 (MPER directed), specific epitope contact positions as well as documented sensitivity/resistance imparting variants at each position were retrieved from CATNAP database (https://www.hiv.lanl.gov/components/sequence/HIV/neutralization/main.comp). Each of the sequences was then assessed for the presence ofsensitive/resistant/undefined mutations at each of these positions using custom bash scripts. In sequence logos, O has been used to differentiate potential N-linked glycosylated asparagine from potentially unglycosylated asparagine (N).

### CombiNAber analysis

Optimal combination prediction was performed with the CombiNAber tool at LABL-HIVDB (https://www.hiv.lanl.gov/content/sequence/COMBINABER/combinaber.html). CombiNAber predictions were made with the IC50 and IC80 neutralization data using the Bliss-Hill model at target concentrations of 10 μg/mL and 1 μg/mL for three distinct specificity bnAb combinations as well as active coverage by at least two bnAbs.

### Statistical analyses and data presentation

Phylogenetic trees were annotated using the “ggtree” package in R. Sequence logos were constructed with the “ggseqlogo” package in R. All plots were prepared using the R package ggplot2. Statistical comparison of variable region characteristics with the Mann-Whitney U-test. Fisher’s test for abundance of bnAb resistance-associated residues was performed through R statistical computing software (v.3.4.0) and R studio v.1.0.143. Statistical analysis for neutralization breadth and potency was done using GraphPad Prism version 10 for Windows, GraphPad Software.

## Supplementary Material

Fig. S1

Fig. S2

Fig. S3

Fig. S4

Fig. S5

Fig. S6

Fig. S7

Fig. S8

Table S1

Table S2

## Figures and Tables

**Table 1:** Source and other properties of contemporary HIV-1 clade C functional clones

	*env (gp160)*	Subtype	Region	Source	Early seroconverters/therapy status	Plasma viral load (copies/mL)	Sample collection year	ART initiation date
1	TSG-EHI6	A1	Nellore	Plasma	Early seroconverters	32,761	2019	NA
2	TSG-EHI9	C	Nellore	Plasma	Early seroconverters	104,713	2019	NA
3	TSG-EHI28	C	Nellore	Plasma	Early seroconverters	140,000	2020	NA
4	TSG-EHI32	C	Nellore	Plasma	Early seroconverters	505,000	2020	NA
5	TSG-EHI38	C	Nellore	Plasma	Early seroconverters	105,000	2021	NA
6	TSG-EHI39	C	Nellore	Plasma	Early seroconverters	196,000	2021	NA
7	TSG-EHI41	C	Nellore	Plasma	Early seroconverters	304,297	2021	NA
8	TSG-EHI42	C	Nellore	Plasma	Early seroconverters	262,078	2021	NA
9	TSG-EHI44	C	Nellore	Plasma	Early seroconverters	16,897	2021	NA
10	TSG-EHI45	C	Nellore	Plasma	Early seroconverters	140,651	2021	NA
11	TSG-EHIPre8	C	Nellore	Plasma	Early seroconverters	414,557	2021	NA
12	TSG-EHI13D6	C	Nellore	Plasma	Early seroconverters	287,618	2020	NA
13	TSG-EHI17B14	C	Nellore	Plasma	Early seroconverters	25,625	2020	NA
14	TSG-EHI8	C	Nellore	Plasma	Early seroconverters	77,078	2019	NA
15	TSG-EHI14	C	Nellore	Plasma	Early seroconverters	642,486	2020	NA
16	TSG-EHI40-C18	C	Nellore	Plasma	Early seroconverters	698,000	2021	NA
17	TSG-EHI11	C	Nellore	Plasma	Early seroconverters	37,513	2020	NA
18	TSG-EHI21	C	Nellore	Plasma	Early seroconverters	82,640	2020	NA
19	TSG-EHI26	A1	Nellore	Plasma	Early seroconverters	31,100	2020	NA
20	TSG-EHI30	C	Nellore	Plasma	Early seroconverters	54,700	2020	NA
21	TSG-EHI18	C	Nellore	Plasma	Early seroconverters	297,757	2020	NA
22	TSG-EHI22	C	Nellore	Plasma	Early seroconverters	9,140	2020	NA
23	TSG-EHI29	C	Nellore	Plasma	Early seroconverters	216,000	2020	NA
24	TSG-EHI35	C	Nellore	Plasma	Early seroconverters	13,526	2020	NA
25	TSG-EHI37	C	Nellore	Plasma	Early seroconverters	35,200	2021	NA
26	TSG-EHI7	C	Nellore	Plasma	Early seroconverters	134,388	2019	NA
27	TSG-EHI12	C	Nellore	Plasma	Early seroconverters	80,381	2020	NA
28	TSG-EHI20	C	Nellore	Plasma	Early seroconverters	156,323	2020	NA
29	TSG-EHI23	C	Nellore	Plasma	Early seroconverters	43,688	2020	NA
30	TSG-EHI27	C	Nellore	Plasma	Early seroconverters	1,500,000	2020	NA
31	TSG-EHI33	C	Nellore	Plasma	Early seroconverters	164,122	2020	NA
32	TSG-EHI55	C	Nellore	Plasma	Early seroconverters	23,200	2022	NA
33	TSG-EHI60	C	Nellore	Plasma	Early seroconverters	22,800	2022	NA
34	TSG-EHI61	C	Nellore	Plasma	Early seroconverters	32,100	2022	NA
35	TSG-EHI62	C	Nellore	Plasma	Early seroconverters	126,000	2022	NA
36	TSG-EHI63	C	Nellore	Plasma	Early seroconverters	6800	2022	NA
37	TSG-EHI25	C	Nellore	Plasma	Early seroconverters	99,400	2020	NA
38	TSG-EHI50	C	Nellore	Plasma	Early seroconverters	401	2021	NA
39	TSG-EHIPre15	C	Nellore	Plasma	Early seroconverters	77,494	2021	NA
40	TSG-EHI51	C	Nellore	Plasma	Early seroconverters	590,637	2021	NA
41	TSG-EHI16	C	Nellore	Plasma	Early seroconverters	66,207	2020	NA
42	TSG-EHI36	C	Nellore	Plasma	Early seroconverters	197,800	2020	NA
43	TSG-EHI34	C	Nellore	Plasma	Early seroconverters	22,800	2020	NA
44	TSG-EHI53	C	Nellore	Plasma	Early seroconverters	7,170	2022	NA
45	TSG-EHI57	C	Nellore	Plasma	Early seroconverters	29,300	2022	NA
46	TSG-EHI58	C	Nellore	Plasma	Early seroconverters	39,400	2022	NA
47	TSG-EHI59	C	Nellore	Plasma	Early seroconverters	31,900	2022	NA
48	TSG21Y02A0012	C	Delhi	Plasma	Receiving therapy	<20	2021	UA
49	TSG21Y02E0018-D18	C	Delhi	Plasma	Therapy naive	4,057	2021	NA
50	TSG21Y02E0024-D24	C	Delhi	Plasma	Therapy naive	7,770	2021	NA
51	TSG22Y02E0035-DE35	C	Delhi	Plasma	Therapy naive	<50	2022	NA
52	TSG22Y02E0037-DE37	C	Delhi	PBMC	Therapy naive	<50	2022	NA
53	TSG21Y02E0011-D11	C	Delhi	Plasma	Therapy naive	<50	2021	NA
54	TSG21Y02E0014-D14	C	Delhi	Plasma	Therapy naive	98,670	2021	NA
55	TSG21S01A0008_STMART07	C	Kolkata	Plasma	Receiving therapy	UA	2021	UA
56	TSG21S01A0013-STMART12	C	Kolkata	Plasma	Receiving therapy	29,296	2021	UA
57	TSG21S01A0003-STMART02	C	Kolkata	Plasma	Receiving therapy	UA	2021	06/2011
58	TSG21S01A0014-STM13ART	C	Kolkata	Plasma	Receiving therapy	699	2021	06/2008
59	TSG21S01E0017-STM16E	C	Kolkata	Plasma	Therapy naive	UA	2021	NA
60	TSG21S01E0001-STM01E	C	Kolkata	Plasma	Therapy naive	UA	2021	NA
61	TSG21S01A0002-STMART01	C	Kolkata	PBMC	Receiving therapy	UA	2021	12/2014
62	TSG21S01E0033-C33	C	Kolkata	PBMC	Therapy naive	UA	2021	NA
63	TSG21S01E0018-STM17E	C	Kolkata	Plasma	Therapy naive	UA	2021	NA
64	TSG21S01A0004-C4	C	Kolkata	Plasma	Receiving therapy	UA	2021	UA
65	TSG21S01A0005-C5	C	Kolkata	Plasma	Receiving therapy	UA	2021	02/2009
66	TSG21N01N017	C	Mumbai	Plasma	Therapy naive	1,927	2021	NA
67	TSG21N01N011-C10	C	Mumbai	Plasma	Therapy naive	269,097	2021	NA
68	TSG21N01N029	C	Mumbai	Plasma	Therapy naive	184,797	2021	NA
69	TSG21N01N018	C	Mumbai	Plasma	Therapy naive	1,331,610	2021	NA
70	TSG21N01S028	C	Mumbai	Plasma	Receiving therapy	<50	2021	01/2017
71	TSG21N01N010	C	Mumbai	Plasma	Therapy naive	29,560	2021	NA
72	TSG21N01N014	C	Mumbai	Plasma	Therapy naive	131,054	2021	NA
73	TSG21N01N023	C	Mumbai	Plasma	Therapy naive	112	2021	NA
74	TSG21N01N031	C	Mumbai	Plasma	Therapy naive	356,394	2021	NA
75	TSG21N01F003	C	Mumbai	Plasma	Receiving therapy	283,195	2021	UA
76	TSG21N01N001CM	C	Mumbai	PBMC	Therapy naive	1,839	2021	NA
77	TSG21N01N013	C	Mumbai	PLASMA	Therapy naive	<50	2021	NA
78	TSG21N01N015	C	Mumbai	Plasma	Therapy naive	1,444,422	2021	NA
79	TSG21N01N025	A1C	Mumbai	Plasma	Therapy naive	6,558	2021	NA
80	TSG21N01N026	C	Mumbai	Plasma	Therapy naive	108,614	2021	NA
81	TSG21N01S007	C	Mumbai	Plasma	Receiving therapy	62	2021	2006
82	TSG21N01S027	C	Mumbai	Plasma	Receiving therapy	1,475	2021	06/2009
83	TSG21N01S030	C	Mumbai	Plasma	Receiving therapy	<50	2021	09/2004
84	TSG21N01S100	C	Mumbai	Plasma	Receiving therapy	UA	2021	09/2010
85	TSG21N01S055	C	Mumbai	Plasma	Receiving therapy	<50	2021	06/2005
86	TSG21N01S062	C	Mumbai	Plasma	Receiving therapy	<50	2021	UA
87	TSG22Y03E0010-B10	C	Bhopal	Plasma	Therapy naive	309,000	2022	NA
88	TSG22Y03A0015-B15	A1	Bhopal	PBMC	Receiving therapy	<50	2022	07/2014
89	TSG22Y03A0022-B22	C	Bhopal	Plasma	Receiving therapy	36,100	2022	04/2019
90	TSG22Y03A0031-B31	C	Bhopal	Plasma	Receiving therapy	87,800	2022	11/2017
91	TSG22Y03A0032-B32	C	Bhopal	Plasma	Receiving therapy	614,000	2022	10/2019
92	TSG22Y03E0019-B19	C	Bhopal	Plasma	Therapy naive	54,700	2022	NA
93	TSG22Y03E0023-B23	C	Bhopal	PBMC	Therapy naive	39,600	2022	NA
94	TSG23Y07A0012-A12	C	Aizawl	PBMC	Receiving therapy	<50	2023	10/2018
95	TSG23Y07A0013-A13	C	Aizawl	PBMC	Receiving therapy	<50	2023	04/2020
96	TSG23Y07A0015-A15	C	Aizawl	Plasma	Receiving therapy	76,500	2023	02/23
97	TSG23Y06A012-V12	C	Vizag	PBMC	Receiving therapy	<50	2023	04/2021
98	TSG23Y06A015-V15	C	Vizag	PBMC	Receiving therapy	<50	2023	06/2022
99	TSG23Y06E001-V1	C	Vizag	Plasma	Therapy naive	<50	2023	NA
100	TSG23Y06A003-V3	C	Vizag	PBMC	Receiving therapy	<50	2023	02/2023
101	TSG23Y06A004-V4	C	Vizag	PBMC	Receiving therapy	<50	2023	02/2023
102	TSG22Y04A0011-H11	C	Hyderabad	PBMC	Receiving therapy	<73	2022	06/2019
103	TSG22Y04A0015-H15	C	Hyderabad	PBMC	Receiving therapy	<73	2022	01/2014
104	TSG22Y04A0016-H16	C	Hyderabad	PBMC	Receiving therapy	1,915	2022	09/2015
105	TSG22Y04A0020-H20	C	Hyderabad	PBMC	Receiving therapy	<73	2022	07/2017
106	TSG22Y04A0002-H2	C	Hyderabad	PBMC	Receiving therapy	<36	2022	05/2018
107	TSG22Y04A0003-H3	C	Hyderabad	PBMC	Receiving therapy	<36	2022	10/2017
108	TSG22Y04A0004-H4	C	Hyderabad	PBMC	Receiving therapy	<73	2022	11/2011
109	TSG22Y04A0005-H5	C	Hyderabad	PBMC	Receiving therapy	1,993	2022	10/2011
110	TSG22Y05A0018-BL18	A1	Belagavi	PBMC	Receiving therapy	39	2022	07/2017
111	TSG22Y05A0021-BL21	C	Belagavi	Plasma	Receiving therapy	25,271	2022	08/2020
112	TSG22Y05A0002-BL2	C	Belagavi	PBMC	Receiving therapy	<50	2022	01/2021
113	TSG22Y05E0033-BL33	C	Belagavi	PBMC	Therapy naive	2,981	2022	NA
114	TSG22Y05E0043-BL43	C	Belagavi	PBMC	Therapy naive	39	2022	NA
115	TSG22Y05A0006-BL6	C	Belagavi	PBMC	Receiving therapy	<50	2022	01/2016

a PBMC, peripheral blood mononuclear cell.

b UA, data unavailable; NA, not applicable.

**Table 2:** Neutralization profiles of pseudoviruses bearing contemporary *envs* broadly resistant to bnAbs with distinct specificities^a^

PSVID	V1/V2 apex directed				CD4bs directed						V3g directed			MPER 10E8	Fusion peptide VRC34.01
	CAP256-VRC26.25	PGDM1400	PG9		VRC01	VRC07	3BNC117	N6	1-18		PGT121	10-1074	BG18		
TSG21N01N017_c18	>25	>25	>25		>25	>25	>25	>25	>25		>25	2.23	>25	17.0	>25
TSG22Y05A0018-BL18	>25	5.44	>25		>25	>25	>25	>25	>25		>25	>25	>25	6.45	>25
TSG-EHI27	>25	>25	>25		>25	>25	>25	0.85	0.75		>25	>25	0.88	2.36	0.67
TSGNO31G	>25	>25	>25		0.84	0.78	>25	0.12	0.04		>25	>25	>25	5.60	>25
TSG-EHI17	>25	>25	0.87		>25	>25	>25	1.67	0.14		0.42	0.04	0.20	>25	>25
TSG22Y05A0006-BL6	>25	>25	2.73		>25	>25	>25	>25	>25		0.02	<0.01	1.83	1.13	>25

a Values indicate IC80 (μg/mL) as determined by TZM-bl pseudovirus neutralization assay. CD4bs refers to CD4 binding site-directed bnAbs; V3g refers to V3 glycan supersite-directed bnAbs; MPER refers to membrane proximal external region of gp41; PSV refers to pseudoviruses.

**Table 3:** Neutralization profiles of the difficult-to-neutralize viruses by two (N6 and BG18) and three (N6, BG18, and PGDM1400) combinations of bnAbs^a^

Pseudoviruses	IC80 values of bnAbs (μg/mL)											
	CAP256-VRC26.25	PG DM 1400	VRC01	VRC07	1-18	N6	3BNC117	PGT121	10-1074	BG18	BG18 + N6	BG18 +N6+PGDM1400
TSG21N01N017_C18	>25	>25	>25	>25	>25	>25	>25	>25	2.23	>25	>25	>25
TSG22Y05A0018-BL18	>25	5.44	>25	>25	>25	>25	>25	>25	>25	>25	>25	2.78
TSG-EHI20	>25	>25	0.39	0.11	0.49	0.07	0.07	>25	>25	>25	1.38	2.45
TSG-EHI27	>25	>25	>25	>25	0.76	0.85	>25	>25	>25	0.89	0.46	0.47
TSG-EHI60	>25	>25	0.11	0.16	0.12	0.07	0.38	>25	>25	>25	0.11	0.33
TSG-EHI36	14.99	0.13	1.37	0.44	>25	0.55	>25	>25	>25	>25	0.19	0.09
TSG21N01N023	>25	>25	0.46	0.12	0.03	0.27	1.25	>25	>25	>25	0.83	1.17
TSG-EHI17B14	>25	>25	>25	>25	0.14	1.67	>25	0.42	0.04	0.21	0.29	0.71
TSG-EHI44	>25	>25	>25	>25	0.02	0.05	>25	12.39	0.23	0.02	0.03	0.1
TSG-EHI37	>25	>25	1	0.25	0.33	0.26	7.86	>25	0.4	0.03	0.11	0.15
TSG-EHI7	>25	>25	10.33	0.56	0.8	2.32	>25	15.97	0.61	1.8	0.7	0.8
TSG-EHI55	>25	>25	1.14	0.31	0.09	0.98	0.23	3.3	1.12	0.31	0.08	0.12
TSG-EHI50	>25	>25	9.75	3.68	1.95	1.32	0.94	>25	>25	>25	9.96	4.07
TSG-EHI34	>25	>25	2.05	0.44	0.12	0.5	1.37	0.14	0.03	0.02	0.04	0.06
TSG-EHI53	0.01	1.8	0.94	0.53	0.26	0.32	1.12	>25	>25	>25	0.9	0.15
TSG21SOIA0004-C4	>25	>25	4.5	0.28	0.9	0.38	2.01	0.35	0.83	0.03	0.06	0.06
TSG21N01N028	>25	>25	0.38	0.08	0.32	0.04	9.51	>25	1.28	0.07	0.06	0.07
TSG21N01N031	>25	>25	0.84	0.78	0.04	0.12	>25	>25	>25	>25	0.18	0.54
TSG21N01F003	>25	>25	3.25	0.73	0.73	0.58	1.26	>25	>25	>25	1.52	2.3
TSG21N01S007	>25	>25	5.28	0.95	12.73	3.66	>25	8.12	1.73	0.04	0.07	0.14
TSG21N01S055	0.01	>25	17	4.46	10.67	1.64	9.76	>25	>25	>25	9.86	0.1
TSG22Y03E0023-B23	0.49	1.14	>25	>25	>25	>25	>25	0.5	0.43	0.04	0.04	0.09
TSG23Y07A0012-Al 2	0.14	6.5	24.06	1.5	1.07	0.74	2.36	>25	>25	>25	5.77	4.6
TSG22Y05E0033-BL33	>25	>25	1.15	0.17	0.35	0.16	>25	0.27	0.14	0.14	0.12	0.28

a Values represent IgG concentrations (μg/mL) of the indicated bnAbs tested that conferred 80% neutralization of the panel pseudoviruses in TZM-bl cells. Note that two (BG18 + N6) and three (BG18 + N6 + PGDM1400) bnAb combinations tested were prepared by mixing them in 1:1 ratio starting from 25 μg/mL of total IgG with subsequent five-fold dilutions up to 0.00032 μg/mL. For two (BG18 + N6) and three (BG18 + N6 + PGDM1400) bnAb combinations, 12.5 μg/mL and 8.33 μg/mL of each of the single bnAbs were mixed as the starting concentration (25 μg/mL).

## Data Availability

Novel *env* nucleotide sequences obtained from Indian donors have been submitted to GenBank (OZ241504-OZ241670, OZ241671-OZ241735). *env* nucleotide sequences from FRESH cohort have been submitted to GenBank (PQ874248-PQ874674).

## Box 1 Importance

While the development of vaccines to prevent HIV infection remains a global priority, their potential effectiveness is limited by the extraordinarily diversified circulating forms of HIV-1. The prospect of best-in-class broadly neutralizing antibodies (bnAbs) as a potential prevention option has been demonstrated in several studies, including the phase 2b Antibody-Mediated Prevention trials; however, to be broadly applicable, bnAbs will need to overcome the substantial variability of HIV *env* circulating globally, beyond the regions where efficacy trials are conducted. The present study highlights that the region-specific contemporary HIV-1 clade C viruses not only vary in their degree of susceptibility to the best-in-class clinically relevant bnAbs, but also are evolving at a population level to become increasingly resistant to the best-in-class bnAbs. Overall, the outcome of this study highlights the need for periodic assessment of sequence and neutralization profiles of the circulating regionally relevant HIV-1 forms toward prioritizing the bnAb combination suitable for effective intervention.

## References

[1] Nair M, Gettins L, Fuller M, Kirtley S, Hemelaar J (2024). Global and regional genetic diversity of HIV-1 in 2010-21: systematic review and analysis of prevalence. Lancet Microbe.

[2] UNAIDS (2023). UNAIDS Global AIDS Update.

[3] Carter A, Zhang M, Tram KH, Walters MK, Jahagirdar D, Brewer ED, Novotney A, Lasher D, Mpolya EA, Vongpradith A (2024). Global, regional, and national burden of HIV/AIDS, 1990-2021, and forecasts to 2050, for 204 countries and territories: the Global Burden of Disease Study 2021. Lancet HIV.

[4] Korber B, Gaschen B, Yusim K, Thakallapally R, Kesmir C, Detours V (2001). Evolutionary and immunological implications of contemporary HIV-1 variation. Br Med Bull.

[5] Bertagnolio S, Hermans L, Jordan MR, Avila-Rios S, Iwuji C, Derache A, Delaporte E, Wensing A, Aves T, Borhan ASM, Leenus A (2021). Clinical impact of pretreat ment human immunodeficiency virus drug resistance in people initiating nonnucleoside reverse transcriptase inhibitor-containing antiretroviral therapy: a systematic review and meta-analysis. J Infect Dis.

[6] Haynes BF, Burton DR, Mascola JR (2019). Multiple roles for HIV broadly neutralizing antibodies. Sci Transl Med.

[7] Corey L, Gilbert PB, Juraska M, Montefiori DC, Morris L, Karuna ST, Edupuganti S, Mgodi NM, deCamp AC, Rudnicki E (2021). Two randomized trials of neutralizing antibodies to prevent HIV-1 acquisition. N Engl J Med.

[8] Gilbert PB, Huang Y, deCamp AC, Karuna S, Zhang Y, Magaret CA, Giorgi EE, Korber B, Edlefsen PT, Rossenkhan R (2022). Neutralization titer biomarker for antibody-mediated prevention of HIV-1 acquisition. Nat Med.

[9] Mkhize NN, Yssel AEJ, Kaldine H, van Dorsten RT, Woodward Davis AS, Beaume N, Matten D, Lambson B, Modise T, Kgagudi P (2023). Neutralization profiles of HIV-1 viruses from the VRC01 Antibody Mediated Prevention (AMP) trials. PLoS Pathog.

[10] Mullick R, Sutar J, Hingankar N, Deshpande S, Thakar M, Sahay S, Ringe RP, Mukhopadhyay S, Patil A, Bichare S, Murugavel KG (2021). Neutralization diversity of HIV-1 Indian subtype C envelopes obtained from cross sectional and followed up individuals against broadly neutralizing monoclonal antibodies having distinct gp120 specificities. Retrovirology (Auckl).

[11] Bouvin-Pley M, Morgand M, Moreau A, Jestin P, Simonnet C, Tran L, Goujard C, Meyer L, Barin F, Braibant M (2013). Evidence for a continuous drift of the HIV-1 species towards higher resistance to neutralizing antibodies over the course of the epidemic. PLoS Pathog.

[12] Bouvin-Pley M, Morgand M, Meyer L, Goujard C, Moreau A, Mouquet H, Nussenzweig M, Pace C, Ho D, Bjorkman PJ, Baty D (2014). Drift of the HIV-1 envelope glycoprotein gp120 toward increased neutralization resistance over the course of the epidemic: a comprehensive study using the most potent and broadly neutralizing monoclonal antibodies. J Virol.

[13] Bunnik EM, Euler Z, Welkers MRA, Boeser-Nunnink BDM, Grijsen ML, Prins JM, Schuitemaker H (2010). Adaptation of HIV-1 envelope gp120 to humoral immunity at a population level. Nat Med.

[14] Rademeyer C, Korber B, Seaman MS, Giorgi EE, Thebus R, Robles A, Sheward DJ, Wagh K, Garrity J, Carey BR (2016). Features of recently transmitted HIV-1 clade C viruses that impact antibody recognition: implications for active and passive immunization. PLoS Pathog.

[15] Rademeyer C, Moore PL, Taylor N, Martin DP, Choge IA, Gray ES, Sheppard HW, Gray C, Morris L, Williamson C (2007). Genetic characteristics of HIV-1 subtype C envelopes inducing cross-neutralizing antibodies. Virology (Auckl).

[16] Ndung’u T, Dong KL, Kwon DS, Walker BD (2018). A FRESH approach: combining basic science and social good. Sci Immunol.

[17] Pham QD, Wilson DP, Law MG, Kelleher AD, Zhang L (2014). Global burden of transmitted HIV drug resistance and HIV-exposure categories: a systematic review and meta-analysis. AIDS.

[18] Sutar J, Deshpande S, Mullick R, Hingankar N, Patel V, Bhattacharya J (2021). Geospatial HIV-1 subtype C gp120 sequence diversity and its predicted impact on broadly neutralizing antibody sensitivity. PLoS One.

[19] Ringe R, Phogat S, Bhattacharya J (2012). Subtle alteration of residues including N-linked glycans in V2 loop modulate HIV-1 neutralization by PG9 and PG16 monoclonal antibodies. Virology (Auckl).

[20] de Taeye SW, Go EP, Sliepen K, de la Peña AT, Badal K, Medina-Ramírez M, Lee W-H, Desaire H, Wilson IA, Moore JP, Ward AB (2019). Stabilization of the V2 loop improves the presentation of V2 loop-associated broadly neutralizing antibody epitopes on HIV-1 envelope trimers. J Biol Chem.

[21] Doria-Rose NA, Georgiev I, O’Dell S, Chuang GY, Staupe RP, McLellan JS, Gorman J, Pancera M, Bonsignori M, Haynes BF, Burton DR (2012). A short segment of the HIV-1 gp120 V1/V2 region is a major determinant of resistance to V1/V2 neutralizing antibodies. J Virol.

[22] Hraber P, Rademeyer C, Williamson C, Seaman MS, Gottardo R, Tang H, Greene K, Gao H, LaBranche C, Mascola JR, Morris L (2017). Panels of HIV-1 subtype C Env reference strains for standardized neutralization assessments. J Virol.

[23] Doria-Rose NA, Bhiman JN, Roark RS, Schramm CA, Gorman J, Chuang G-Y, Pancera M, Cale EM, Ernandes MJ, Louder MK (2016). New member of the V1V2-directed CAP256-VRC26 lineage that shows increased breadth and exceptional potency. J Virol.

[24] Gorman J, Chuang G-Y, Lai YT, Shen C-H, Boyington JC, Druz A, Geng H, Louder MK, McKee K, Rawi R, Verardi R (2020). Structure of super-potent antibody CAP256-VRC26.25 in complex with HIV-1 envelope reveals a combined mode of trimer-apex recognition. Cell Rep.

[25] Zhou P, Wang H, Fang M, Li Y, Wang H, Shi S, Li Z, Wu J, Han X, Shi X, Shang H (2019). Broadly resistant HIV-1 against CD4-binding site neutralizing antibodies. PLoS Pathog.

[26] Horwitz JA, Halper-Stromberg A, Mouquet H, Gitlin AD, Tretiakova A, Eisenreich TR, Malbec M, Gravemann S, Billerbeck E, Dorner M, Büning H (2013). HIV-1 suppression and durable control by combining single broadly neutralizing antibodies and antiretroviral drugs in humanized mice. Proc Natl Acad Sci USA.

[27] Julg B, Pegu A, Abbink P, Liu J, Brinkman A, Molloy K, Mojta S, Chandrashekar A, Callow K, Wang K, Chen X (2017). Virological control by the CD4-binding site antibody N6 in simian-human immunodeficiency virus-infected rhesus monkeys. J Virol.

[28] Wagh K, Seaman MS (2023). Divide and conquer: broadly neutralizing antibody combinations for improved HIV-1 viral coverage. Curr Opin HIV AIDS.

[29] Gruell H, Schommers P (2023). Advancing bnAb combinations for HIV prevention. Lancet HIV.

[30] Julg B, Walker-Sperling VEK, Wagh K, Aid M, Stephenson KE, Zash R, Liu J, Nkolola JP, Hoyt A, Castro M (2024). Safety and antiviral effect of a triple combination of HIV-1 broadly neutralizing antibodies: a phase 1/2a trial. Nat Med.

[31] Julg B, Stephenson KE, Wagh K, Tan SC, Zash R, Walsh S, Ansel J, Kanjilal D, Nkolola J, Walker-Sperling VEK (2022). Safety and antiviral activity of triple combination broadly neutralizing monoclonal antibody therapy against HIV-1: a phase 1 clinical trial. Nat Med.

[32] Wagh K, Bhattacharya T, Williamson C, Robles A, Bayne M, Garrity J, Rist M, Rademeyer C, Yoon H, Lapedes A, Gao H (2016). Optimal combinations of broadly neutralizing antibodies for prevention and treatment of HIV-1 clade C infection. PLoS Pathog.

[33] Guo C, Wu Y, Zhang Y, Liu X, Li A, Gao M, Zhang T, Wu H, Chen G, Huang X (2021). Transmitted drug resistance in antiretroviral therapy-naive persons with acute/early/primary HIV infection: a systematic review and meta-analysis. Front Pharmacol.

[34] Kulkarni SS, Lapedes A, Tang H, Gnanakaran S, Daniels MG, Zhang M, Bhattacharya T, Li M, Polonis VR, McCutchan FE, Morris L (2009). Highly complex neutralization determinants on a monophyletic lineage of newly transmitted subtype C HIV-1 Env clones from India. Virology (Auckl).

[35] Ringe R, Thakar M, Bhattacharya J (2010). Variations in autologous neutralization and CD4 dependence of b12 resistant HIV-1 clade C env clones obtained at different time points from antiretroviral naïve Indian patients with recent infection. Retrovirology (Auckl).

[36] Lee S, Nguyen LT, Hayes BJ, Ross EM (2021). Prowler: a novel trimming algorithm for Oxford Nanopore sequence data. Bioinformatics.

[37] Lole KS, Bollinger RC, Paranjape RS, Gadkari D, Kulkarni SS, Novak NG, Ingersoll R, Sheppard HW, Ray SC (1999). Full-length human immunodeficiency virus type 1 genomes from subtype C-infected seroconverters in India, with evidence of intersubtype recombination. J Virol.

[38] Li H (2018). Minimap2: pairwise alignment for nucleotide sequences. Bioinformatics.

[39] Li H (2021). New strategies to improve minimap2 alignment accuracy. Bioinformatics.

[40] Danecek P, Bonfield JK, Liddle J, Marshall J, Ohan V, Pollard MO, Whitwham A, Keane T, McCarthy SA, Davies RM, Li H (2021). Twelve years of SAMtools and BCFtools. Gigascience.

[41] Sahlin K, Medvedev P (2020). De novo clustering of long-read transcriptome data using a greedy, quality value-based algorithm. J Comput Biol.

[42] Sahlin K, Medvedev P (2021). Error correction enables use of Oxford Nanopore technology for reference-free transcriptome analysis. Nat Commun.

[43] Grubaugh ND, Gangavarapu K, Quick J, Matteson NL, De Jesus JG, Main BJ, Tan AL, Paul LM, Brackney DE, Grewal S, Gurfield N (2019). An amplicon-based sequencing framework for accurately measuring intrahost virus diversity using PrimalSeq and iVar. Genome Biol.

[44] Walker BJ, Abeel T, Shea T, Priest M, Abouelliel A, Sakthikumar S, Cuomo CA, Zeng Q, Wortman J, Young SK, Earl AM (2014). Pilon: an integrated tool for comprehensive microbial variant detection and genome assembly improvement. PLoS One.

[45] Patil S, Kumar R, Deshpande S, Samal S, Shrivastava T, Boliar S, Bansal M, Chaudhary NK, Srikrishnan AK, Murugavel KG, Solomon S (2016). Conformational epitope-specific broadly neutralizing plasma antibodies obtained from an HIV-1 clade C-infected elite neutralizer mediate autologous virus escape through mutations in the V1 loop. J Virol.

[46] Gharu L, Ringe R, Pandey S, Paranjape R, Bhattacharya J (2009). HIV-1 clade C env clones obtained from an Indian patient exhibiting expanded coreceptor tropism are presented with naturally occurring unusual amino acid substitutions in V3 loop. Virus Res.

[47] Gharu L, Ringe R, Satyakumar A, Patil A, Bhattacharya J (2011). Short communication: evidence of HIV type 1 clade C env clones containing low V3 loop charge obtained from an AIDS patient in India that uses CXCR6 and CCR8 for entry in addition to CCR5. AIDS Res Hum Retroviruses.

[48] Ritz C, Baty F, Streibig JC, Gerhard D (2015). Dose-response analysis using R. PLoS One.

[49] Trifinopoulos J, Nguyen L-T, von Haeseler A, Minh BQ (2016). W-IQ-TREE: a fast online phylogenetic tool for maximum likelihood analysis. Nucleic Acids Res.

[50] Nguyen L-T, Schmidt HA, von Haeseler A, Minh BQ (2015). IQ-TREE: a fast and effective stochastic algorithm for estimating maximum-likelihood phylogenies. Mol Biol Evol.

