# Supplementary material for: Distinct region-specific neutralization profiles of contemporary HIV-1 clade C against best-in-class broadly neutralizing antibodies
Source: J Virol. 2025 May 16;99(6):e00008-25. doi: 10.1128/jvi.00008-25 (PMC7617755; doi:10.1128/jvi.00008-25)
Supplement: Fig. S1 — Hierarchical clustering using heatmap depicting the magnitude of neutralization sensitivity of contemporary HIV-1 India clade C viruses against 14 bnAbs with distinct epitope specificities. [file jvi.00008-25-s0001.pdf]

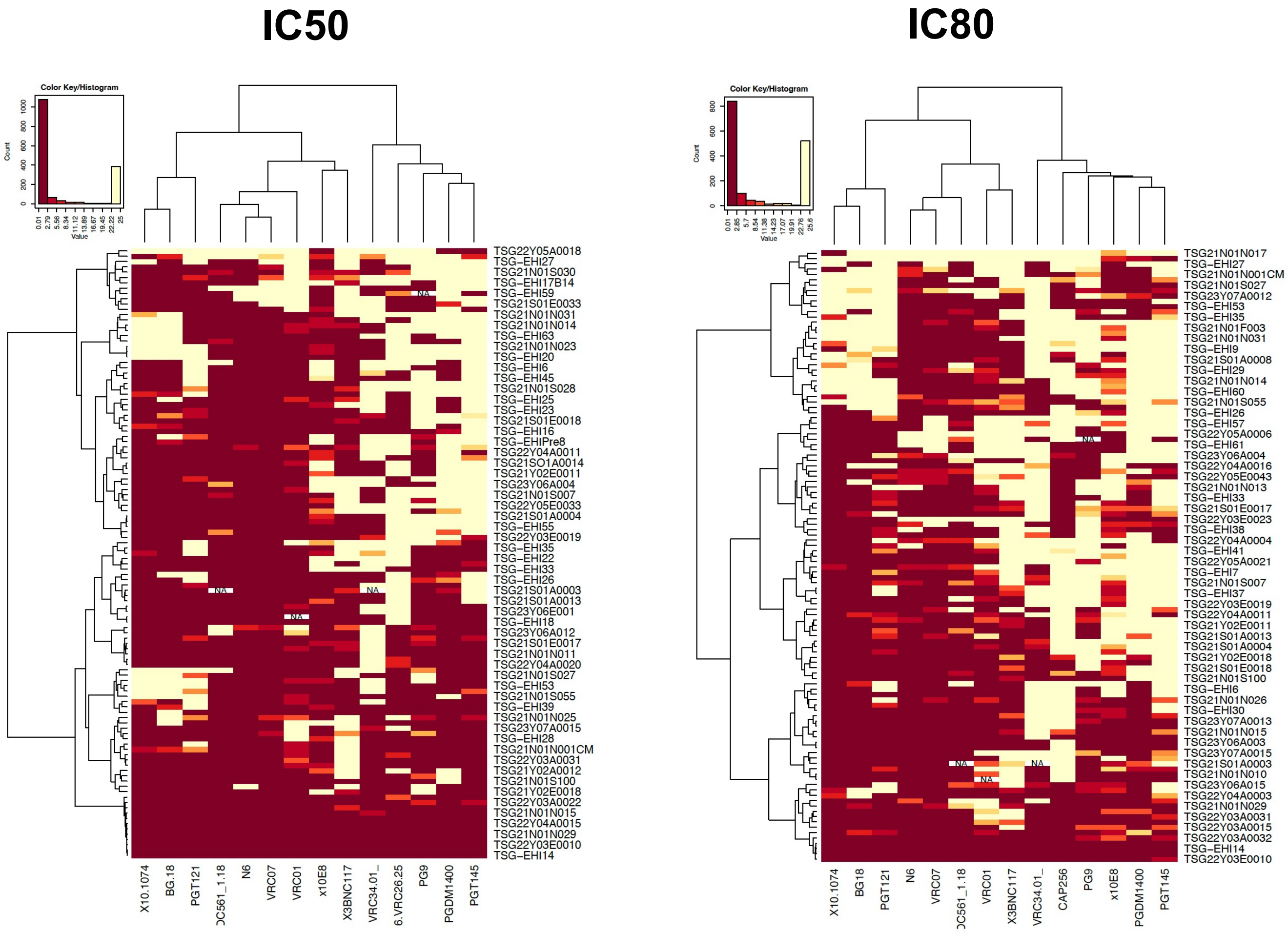

Fig.S1. Hierarchal clustering using heatmap depicting the magnitude (https://www.hiv.lanl.gov/content/sequence/HEATMAP/heatmap.html)of neutralization sensitivity of contemporary HIV-1 India clade C viruses against 14 bnAbs with distinct epitope specificities. Heatmap was prepared using IC50 and IC80 values (ug/mL).
