# Supplementary material for: Distinct region-specific neutralization profiles of contemporary HIV-1 clade C against best-in-class broadly neutralizing antibodies
Source: J Virol. 2025 May 16;99(6):e00008-25. doi: 10.1128/jvi.00008-25 (PMC7617755; doi:10.1128/jvi.00008-25)
Supplement: Fig. S2 — Comparison of Env variable loop, PNGS, and net charges between South Africa clade C viruses that showed sensitivity and resistance to CAP256-VRC26.25 and PGDM1400. [file jvi.00008-25-s0002.pdf]

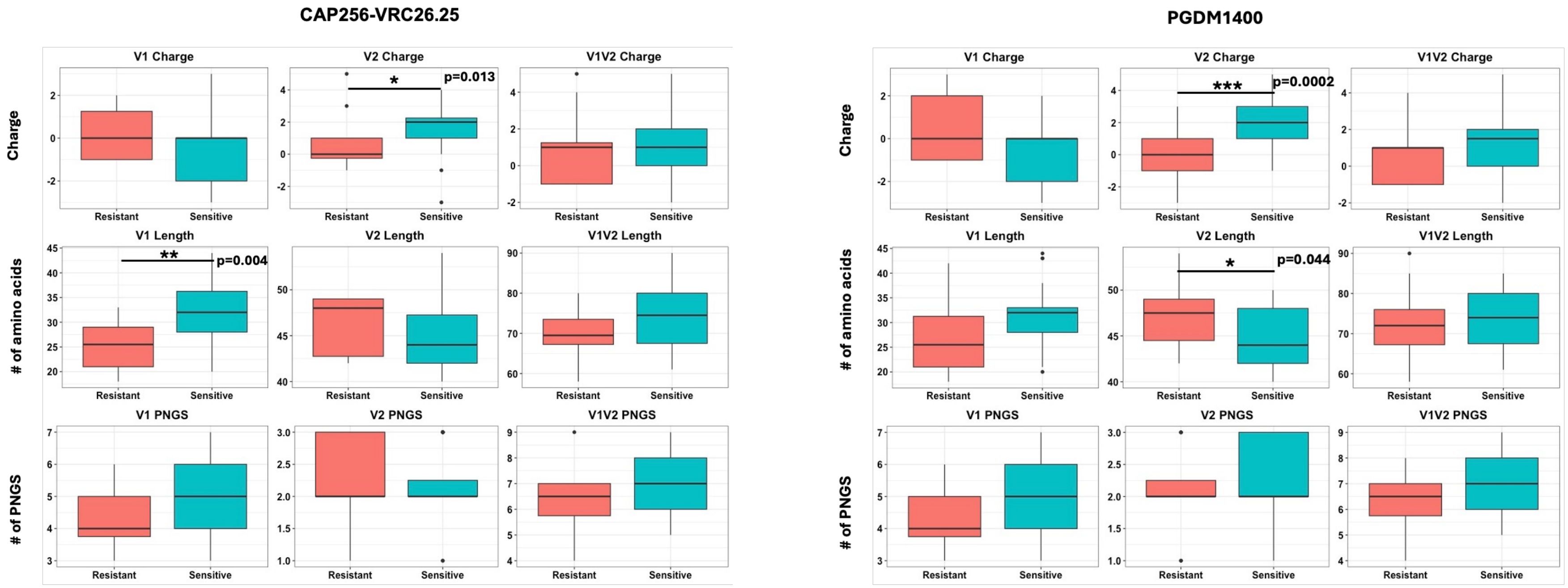

SA sensitive vs SA resistant

Fig. S2. Comparison of Env variable loop, PNGS and net charges between South Africa clade C viruses that showed sensitivity and resistance to CAP256-VRC26.25 and PGDM1400.
