# Supplementary material for: Distinct region-specific neutralization profiles of contemporary HIV-1 clade C against best-in-class broadly neutralizing antibodies
Source: J Virol. 2025 May 16;99(6):e00008-25. doi: 10.1128/jvi.00008-25 (PMC7617755; doi:10.1128/jvi.00008-25)
Supplement: Fig. S3 — Sequence features of India clade C viruses encoding contemporary envs sensitive and resistant to V3 glycan supersite-directed bnAbs. [file jvi.00008-25-s0003.pdf]

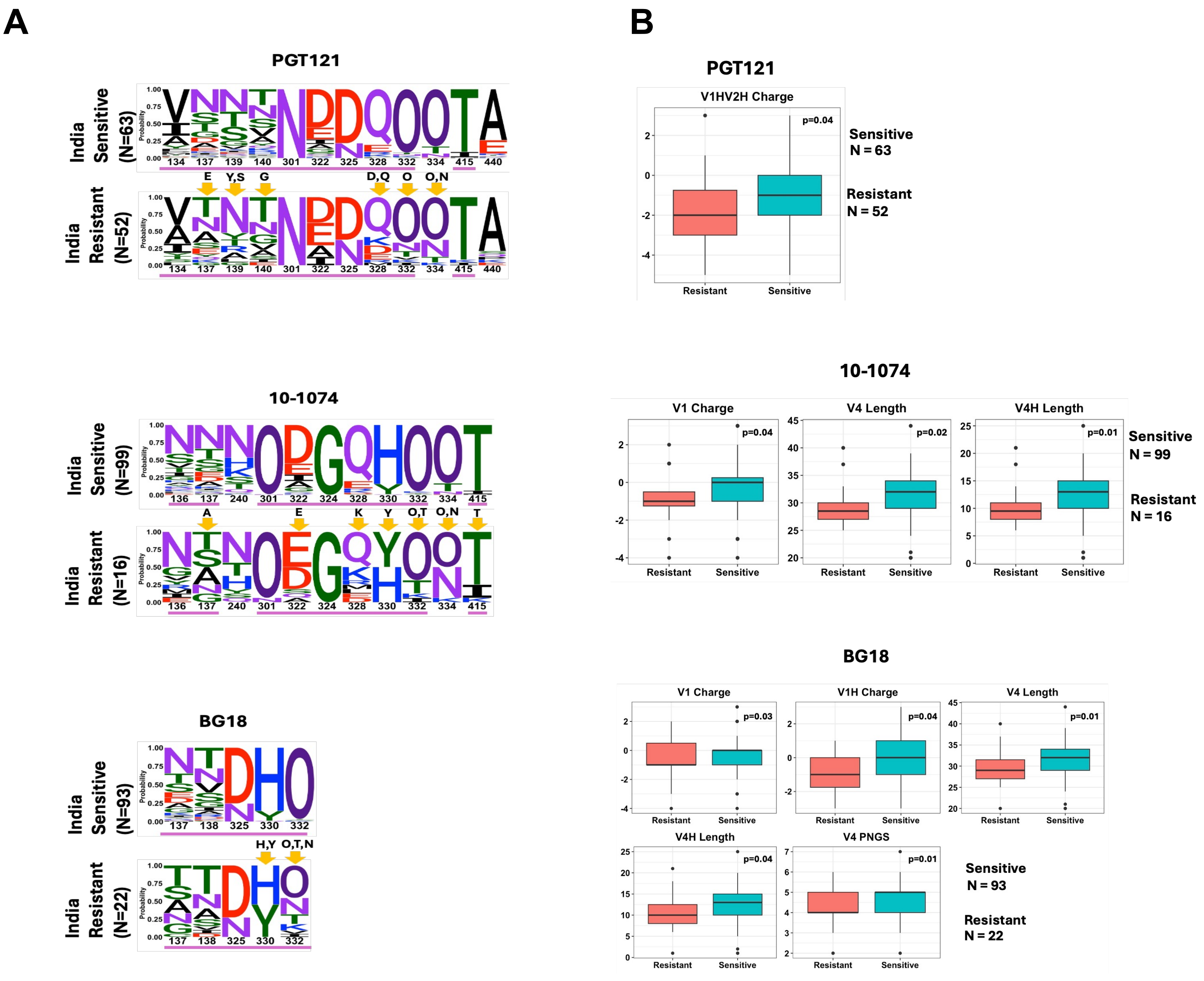

Fig. S3. Sequence features of India clade C viruses encoding contemporary envs sensitive and resistant to V3 glycan supersite directed bnAbs. A. Sequence logos of PGT121, 10-1074 and BG18 sensitive and resistant viruses. B. Variable loop, PNGs and net charge characteristics between sensitive and resistant viruses.
