# Supplementary material for: Distinct region-specific neutralization profiles of contemporary HIV-1 clade C against best-in-class broadly neutralizing antibodies
Source: J Virol. 2025 May 16;99(6):e00008-25. doi: 10.1128/jvi.00008-25 (PMC7617755; doi:10.1128/jvi.00008-25)
Supplement: Fig. S4 — Sequence logo of India clade C viruses encoding contemporary envs sensitive and resistant to the CD4bs directed bnAbs. [file jvi.00008-25-s0004.pdf]

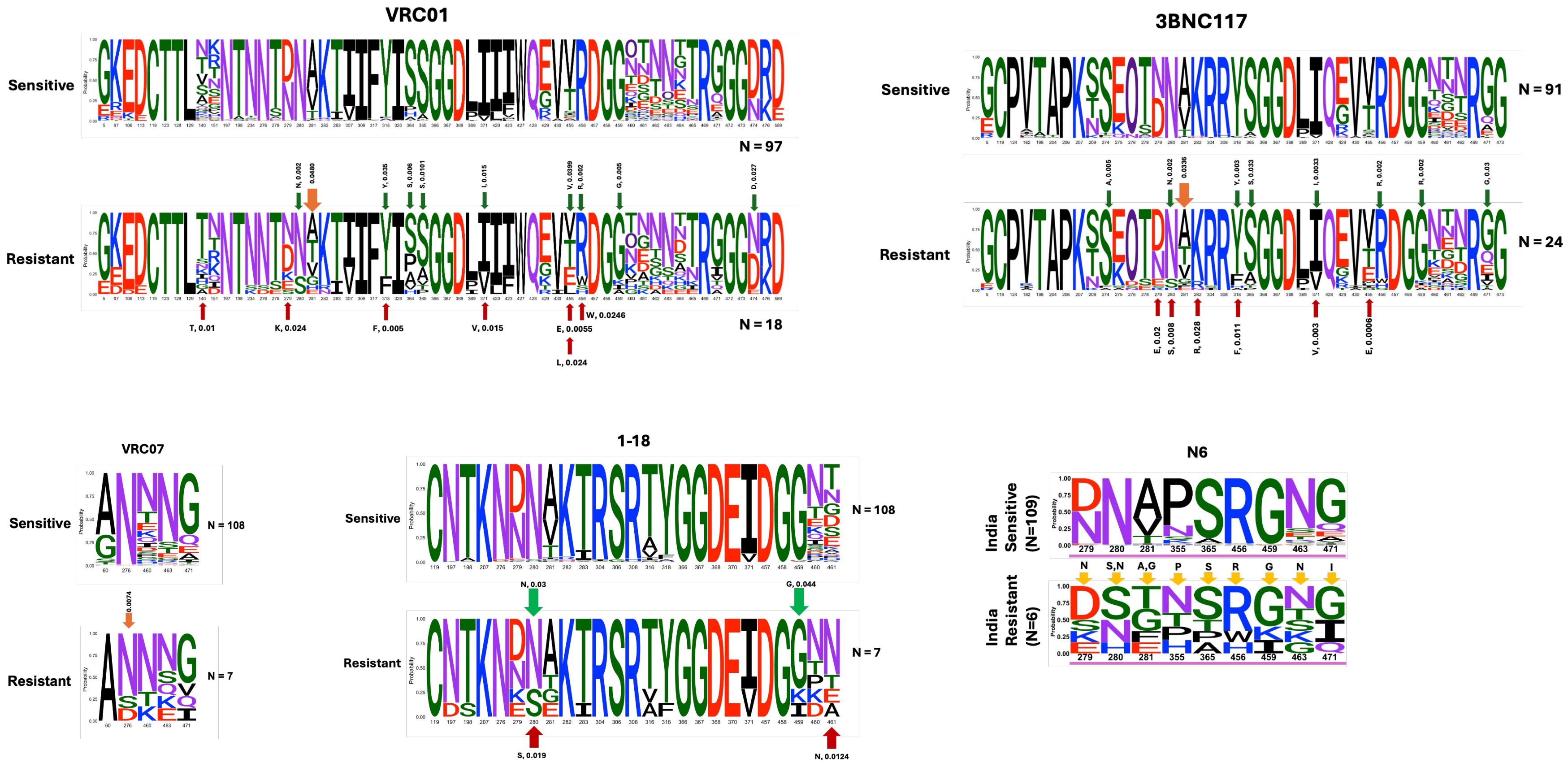

Fig. S4. Sequence logo of India clade C viruses encoding contemporary envs sensitive and resistant to the CD4bs directed bnAbs.
