# Supplementary material for: Distinct region-specific neutralization profiles of contemporary HIV-1 clade C against best-in-class broadly neutralizing antibodies
Source: J Virol. 2025 May 16;99(6):e00008-25. doi: 10.1128/jvi.00008-25 (PMC7617755; doi:10.1128/jvi.00008-25)
Supplement: Fig. S6 — Comparison of variable loop length, PNLGs, and net charge between Indian and South Africa clade C viruses resistant to CAP256-VRC26.25 and PGDM1400. [file jvi.00008-25-s0006.pdf]

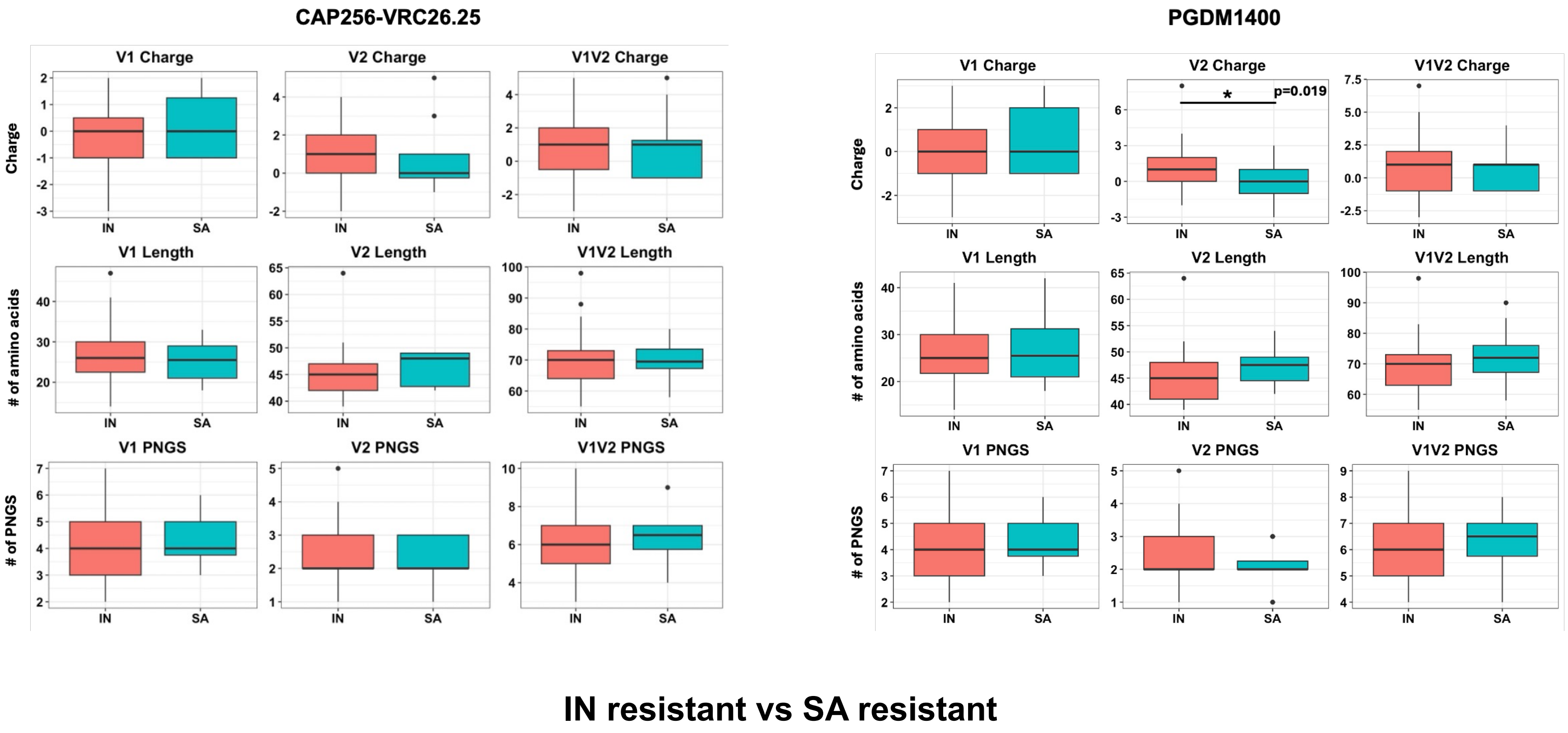

Fig. S6. Comparison of variable loop length, PNLGs and net charge between Indian and South Africa clade C viruses resistant to CAP256-VRC26.25 and PGDM1400.
