# Supplementary material for: Distinct region-specific neutralization profiles of contemporary HIV-1 clade C against best-in-class broadly neutralizing antibodies
Source: J Virol. 2025 May 16;99(6):e00008-25. doi: 10.1128/jvi.00008-25 (PMC7617755; doi:10.1128/jvi.00008-25)
Supplement: Fig. S7 — Comparison of frequency of key contact residues on envs that when expressed as pseudoviruses of India and South Africa origins showed resistance to V3 glycan-directed bnAbs (PGT121, 10-1074, BG18) and CD4bs directed bnAb (N6). [file jvi.00008-25-s0007.pdf]

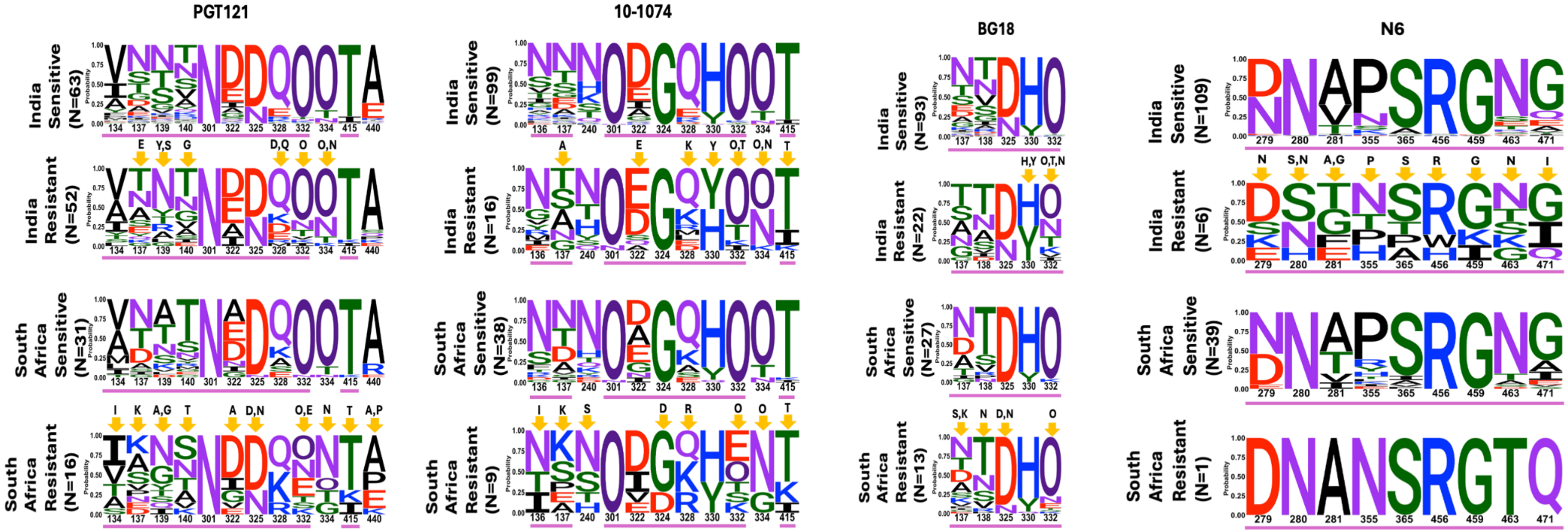

Fig. S7. Comparison of frequency of key contact residues on envs that when expressed as pseudoviruses of India (N=115) and South Africa (N=40) origins showed resistance to key V3 glycan directed (PGT121, 10-1074, BG18) and CD4bs (N6) directed bnAbs.
