# Supplementary material for: Distinct region-specific neutralization profiles of contemporary HIV-1 clade C against best-in-class broadly neutralizing antibodies
Source: J Virol. 2025 May 16;99(6):e00008-25. doi: 10.1128/jvi.00008-25 (PMC7617755; doi:10.1128/jvi.00008-25)
Supplement: Fig. S8 — Abundance of CAP256-VRC26.25/PGDM1400 sensitivity/resistance associated residues in sequences from India and South Africa that have not been tested through in vitro neutralization assays. [file jvi.00008-25-s0008.pdf]

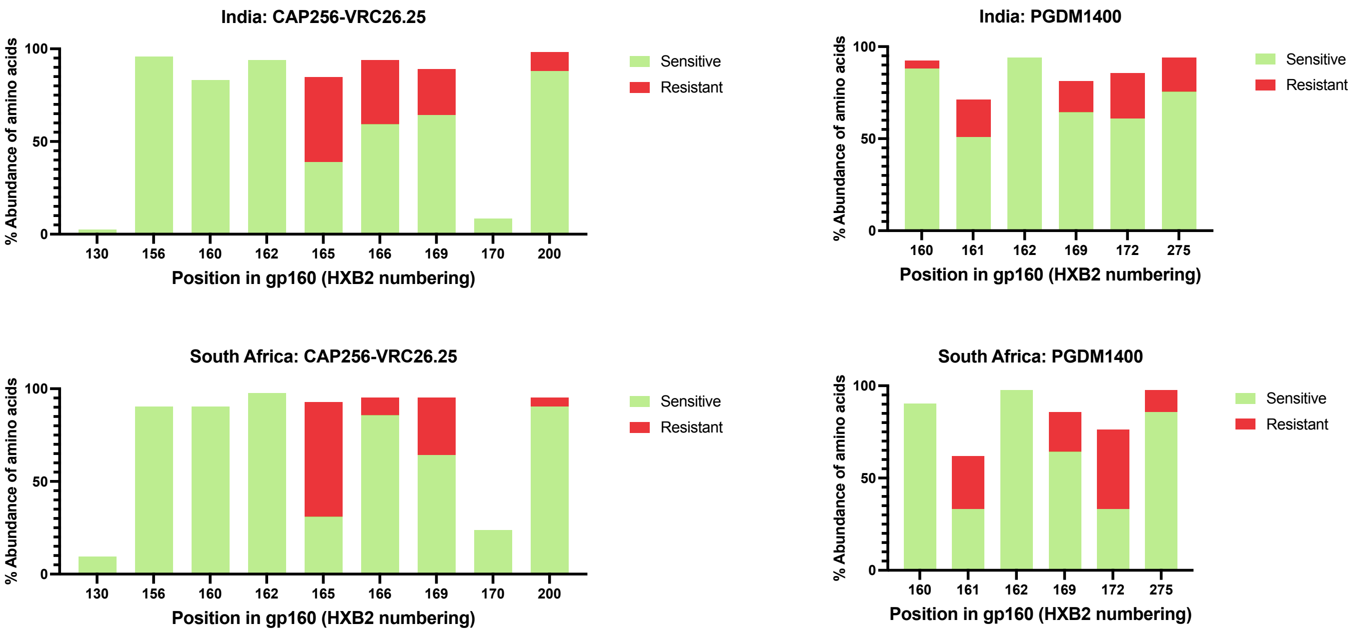

<table>
<tr><td>gp160_pos</td><td>130</td><td>156</td><td>160</td><td>162</td><td>165</td><td>166</td><td>169</td><td>170</td><td>200</td></tr>
<tr><td>Sensitive</td><td>K</td><td>NLG</td><td>NLG</td><td>T</td><td>L</td><td>R</td><td>R|K</td><td>R</td><td>T|A</td></tr>
<tr><td>Resistant</td><td>und</td><td>und</td><td>und</td><td>und</td><td>I</td><td>G|I|Q|S|T|K</td><td>E|V|T|Q</td><td>und</td><td>V</td></tr>
</table>

<table>
<tr><td>gp160_pos</td><td>160</td><td>161</td><td>162</td><td>169</td><td>172</td><td>275</td></tr>
<tr><td>Sensitive</td><td>NLG</td><td>A|M</td><td>T</td><td>K|R</td><td>V</td><td>E</td></tr>
<tr><td>Resistant</td><td>K|T|D</td><td>I|V</td><td>I|N</td><td>E|Q|V</td><td>E|D</td><td>K</td></tr>
</table>

Fig. S8. Abundance of CAP256-VRC26.25/PGDM1400 sensitivity/resistance associated residues in sequences from India (N=118) and South Africa (N=41) that have not been tested through in vitro neutralization assays. X axes define amino acid positions in gp160 whereas Y axes define percent abundance of residues. The tables indicate the residues associated with sensitivity or resistance. Und: undefined.
