# Supplementary material for: Distinct region-specific neutralization profiles of contemporary HIV-1 clade C against best-in-class broadly neutralizing antibodies
Source: J Virol. 2025 May 16;99(6):e00008-25. doi: 10.1128/jvi.00008-25 (PMC7617755; doi:10.1128/jvi.00008-25)
Supplement: Table S1 — Demographic details of nine different geographical regions in India, risk groups, and ART status of HIV+ donors. [file jvi.00008-25-s0009.docx]

| **Viruses** | **Site** | **Age** | **Sex** | **ART status** | **Viral load** | **CD4** | **Date of enrolment** | **Date of ART initiation** | **Mode of Transmission** | **PR DRMs** | **RT DRMs** | **IN DRMs** |
| --- | --- | --- | --- | --- | --- | --- | --- | --- | --- | --- | --- | --- |
| TSG22Y03A0001-B1 | Bhopal | 43 | M | Receiving ART | Undetectable | 1183 | 2022 | 2016 | MSM | **M46I, D30N** | **M184I, E138K** | **E138K** |
| TSG22Y03E0010-B10 | Bhopal | 22 | M | ART Naïve | 309000 | 372 | 2022 | NA | MSM | None | None | None |
| TSG22Y03A0015-B15 | Bhopal | 32 | M | Receiving ART | Undetectable | 492 | 2022 | 2014 | MSM | **M46I, D30N** | **M184I, E138K** | **E138K, R263K** |
| TSG22Y03A0018-B18 | Bhopal | 43 | M | Receiving ART | Undetectable | 246 | 2022 | 2011 | MSM | None | **M184I** | None |
| TSG22Y03E0019-B19 | Bhopal | 28 | M | ART Naïve | 54700 | 491 | 2022 | NA | MSM | **Q58E** | None | None |
| TSG22Y03A0002-B2 | Bhopal | 32 | M | Receiving ART | Undetectable | 236 | 2022 | 2017 | MSM | **M46I, D30N** | **Y115F, M184I, E138K, D67N** | **E138K** |
| TSG22Y03A0022-B22 | Bhopal | 39 | M | Receiving ART | 36100 | 349 | 2022 | 2019 | MSM | None | **K103N** | None |
| TSG22Y03E0023-B23 | Bhopal | 34 | M | ART Naïve | 39600 | 331 | 2022 | NA | MSM | **M46I, D30N** | M184I, E138K | None |
| TSG22Y03A0024-B24 | Bhopal | 36 | M | Receiving ART | 117000 | 167 | 2022 | 2017 | MSM | None | **K103N** | None |
| TSG22Y03A0028-B28 | Bhopal | 31 | M | Receiving ART | 51100 | 408 | 2022 | 2017 | MSM | M46I, D30N | None | None |
| TSG22Y03A0003-B3 | Bhopal | 26 | M | Receiving ART | 22 | 718 | 2022 | 2018 | MSM | V32I | **M184I** | **R263K** |
| TSG22Y03E0030-B30 | Bhopal | 30 | M | ART Naïve | 24500 | 788 | 2022 | NA | MSM | None | None | None |
| TSG22Y03A0031-B31 | Bhopal | 31 | M | Receiving ART | 87800 | 294 | 2022 | 2017 | MSM | None | K65E, K103N | None |
| TSG22Y03A0032-B32 | Bhopal | 31 | M | Receiving ART | 614000 | 330 | 2022 | 2019 | MSM | None | None | None |
| TSG22Y03A0036-B36 | Bhopal | 30 | M | Receiving ART | 420000 | 401 | 2022 | 2018 | MSM | ND | ND | ND |
| TSG22Y03A0037-B37 | Bhopal | 28 | M | Receiving ART | 59500 | 309 | 2022 | 2019 | MSM | None | M184I | None |
| TSG22Y03E0039-B39 | Bhopal | 31 | M | ART Naïve | 66800 | 307 | 2022 | NA | MSM | None | None | None |
| TSG22Y03A0004-B4 | Bhopal | 39 | M | Receiving ART | Undetectable | 743 | 2022 | 2018 | MSM | None | **M184I** | **R263K** |
| TSG22Y03A0005-B5 | Bhopal | 36 | M | Receiving ART | Undetectable | 320 | 2022 | 2021 | MSM | None | None | None |
| TSG22Y03A0006-B6 | Bhopal | 25 | M | Receiving ART | Undetectable | 910 | 2022 | 2018 | MSM | ND | ND | ND |
| TSG21S01A0011-C11 | Kolkata | 52 | M | Receiving ART | 7087 | ND | 2021 | 2007 | HETEROSEXUAL | None | None | None |
| TSG21S01A0015-C15 | Kolkata | 41 | M | Receiving ART | ND | ND | 2021 | 2017 | HETEROSEXUAL | ND | ND | ND |
| TSG21S01A0025-C25 | Kolkata | 38 | F | Receiving ART | ND | ND | 2021 | 2019 | HETEROSEXUAL | M46I | **M184I** | E138K, **R263K** |
| TSG21S01A0026-C26 | Kolkata | 36 | M | Receiving ART | ND | ND | 2021 | 2010 | HETEROSEXUAL | **M46I, D30N** | **E138A, M184I** | None |
| TSG21S01E0033-C33 | Kolkata | 28 | M | ART Naïve | ND | ND | 2021 | NA | HETEROSEXUAL | None | None | None |
| TSG21S01A0035-C35 | Kolkata | 48 | M | Receiving ART | ND | ND | 2021 | 2013 | HETEROSEXUAL | **M46I, D30N** | **M184I**, E138K | None |
| TSG21S01E0036-C36 | Kolkata | 59 | M | ART Naïve | ND | ND | 2021 | NA | HETEROSEXUAL | None | **G190A, Y181C, M41L, Y181V, M184V, T215Y** | None |
| TSG21S01A0037-C37 | Kolkata |  |  | Receiving ART | ND | ND | 2021 | 2019 | ND | None | None | None |
| TSG21S01A0004-C4 | Kolkata | 45 | M | ART Naïve | ND | ND | 2021 | NA | HETEROSEXUAL | None | None | None |
| TSG21S01A0006-C6 | Kolkata | 50 | M | Receiving ART | ND | ND | 2021 | 2008 | HETEROSEXUAL | None | None | None |
| TSG21Y02A0010-D10 | Delhi | 33 | M | Receiving ART | Undetectable | ND | 2021 | ND | PWID | ND | ND | ND |
| TSG21Y02E0011-D11 | Delhi | 25 | M | ART Naïve | Undetectable | 407 | 2021 | NA | MSM | ND | ND | ND |
| TSG21Y02E0014-D14 | Delhi | 29 | M | ART Naïve | 98670.8 | 256.0 | 2021 | NA | MSM | None | None | None |
| TSG21Y02E0018-D18 | Delhi | 27 | M | ART Naïve | 4057 | 618 | 2021 | NA | MSM | None | None | None |
| TSG21Y02E0019-D19 | Delhi | 32 | M | ART Naïve | Undetectable | 648.0 | 2021.0 | NA | MSM | None | None | None |
| TSG21Y02E0024-D24 | Delhi | 30 | M | ART Naïve | 7770.0 | 337.0 | 2021.0 | NA | MSM | None | **K103N** | None |
| TSG22Y02A0057-D57 | Delhi | ND | ND | Receiving ART | 2545 | 504 | 2022 | ND | ND | None | None | None |
| TSG21Y02A0006-D6 | Delhi | 42 | M | Receiving ART | Undetectable | 4441.0 | 2021.0 | ND | MSM | ND | ND | ND |
| TSG22Y02A0033-DA33 | Delhi | 28 | M | Receiving ART | 12092 | 440 | 2022 | ND | MSM | None | None | None |
| TSG22Y02E0035-DE35 | Delhi | 24 | M | ART Naïve | Undetectable | 445 | 2022 | NA | MSM | None | **K65R** | None |
| TSG22Y02E0036-DE36 | Delhi | 24 | M | ART Naïve | Undetectable | 531 | 2022 | NA | MSM | None | **E138A** | None |
| TSG22Y02E0037-DE37 | Delhi | 25 | M | ART Naïve | 139463 | 518 | 2022 | NA | MSM | M46I, D30N | **M184I** | None |
| TSG22Y02E0039-DE39 | Delhi | 27 | M | ART Naïve | 12016 | 640 | 2022 | NA | MSM | **M46I, D30N** | **E138A, G190E, M184I, D67N** | **E138K** |
| TSG22Y01A0019-NA19 | Nellore | ND | ND | Receiving ART | ND | ND | 2022 | ND | ND | None | None | R263K |
| TSG21Y01A0002-NA2 | Nellore | 34 | F | Receiving ART | Undetectable | 730 | 2021 | ND | HETEROSEXUAL | None | None | None |
| TSG21Y01A0003-NA3 | Nellore | 45 | M | Receiving ART | Undetectable | 353 | 2021 | ND | MSM | None | None | None |
| TSG21Y01A0007-NA7 | Nellore | 42 | M | Receiving ART | Undetectable | 1152 | 2021 | ND | MSM | None | None | None |
| TSG21Y01E0001-NE1 | Nellore | 26 | F | ART Naïve | 81,300 | 279 | 2022 | NA | HETEROSEXUAL | None | None | **R263K** |
| TSG22Y01E0010-NE10 | Nellore | 34 | M | ART Naïve | 57,900 | 68 | 2022 | NA | MSM | None | None | None |
| TSG21Y01E0002-NE2 | Nellore | 44 | F | ART Naïve | 144,438 | 240 | 2021 | NA | HETEROSEXUAL | None | M184I | None |
| TSG21Y01E0003-NE3 | Nellore | 33 | F | ART Naïve | 1938 | 425 | 2021 | NA | HETEROSEXUAL | None | **M184I, E138K** | None |
| TSG21Y01E0004-NE4 | Nellore | 28 | M | ART Naïve | 19,817 | 116 | 2021 | NA | MSM | None | None | None |
| TSG22Y01E0008-NE8 | Nellore | 34 | F | ART Naïve | ND | 457 | 2022 | NA | HETEROSEXUAL | None | M184I, K65R | None |
| TSG-EHI11 | Nellore | 24 | M | ART Naïve | 37,513 | 453 | 2020 | NA | MSM | None | None | None |
| TSG-EHI12 | Nellore | 22 | M | ART Naïve | 80,381 | 505 | 2020 | NA | MSM | F53L | M184I | None |
| TSG-EHI16 | Nellore | 19 | M | ART Naïve | 66,207 | 421 | 2020 | NA | MSM | ND | ND | ND |
| TSG-EHI18 | Nellore | 25 | M | ART Naïve | 297,757 | 333 | 2020 | NA | MSM | None | F77L, H221Y | None |
| TSG-EHI20 | Nellore | 31 | M | ART Naïve | 156,323 | 341 | 2020 | NA | MSM | None | None | None |
| TSG-EHI21 | Nellore | 24 | M | ART Naïve | 82,640 | 308 | 2020 | NA | MSM | None | M184I | None |
| TSG-EHI22 | Nellore | 43 | M | ART Naïve | 9,140 | 671 | 2020 | NA | HETEROSEXUAL | I50V | M184I | None |
| TSG-EHI23 | Nellore | 27 | M | ART Naïve | 43,688 | 474 | 2020 | NA | MSM | None | M184I | None |
| TSG-EHI26 | Nellore | 31 | M | ART Naïve | 31,100 | 318 | 2020 | NA | HETEROSEXUAL | None | **K103N** | None |
| TSG-EHI27 | Nellore | 28 | F | ART Naïve | 1,500,000 | 238 | 2020 | NA | HETEROSEXUAL | ND | **L33F** | ND |
| TSG-EHI29 | Nellore | 41 | M | ART Naïve | 216,000 | 337 | 2020 | NA | HETEROSEXUAL | ND | M184I | ND |
| TSG-EHI30 | Nellore | 29 | F | ART Naïve | 54,700 | 350 | 2020 | NA | HETEROSEXUAL | None | None | None |
| TSG-EHI33 | Nellore | 32 | M | ART Naïve | 164,122 | 479 | 2020 | NA | HETEROSEXUAL | ND | M184V | ND |
| TSG-EHI35 | Nellore | 24 | M | ART Naïve | 13,526 | 375 | 2020 | NA | MSM | ND | **Y115F** | ND |
| TSG-EHI37 | Nellore | 38 | F | ART Naïve | 35,200 | 726 | 2020 | NA | HETEROSEXUAL | ND | M184I | ND |
| TSG-EHI6 | Nellore | 27 | M | ART Naïve | 32,761 | 592 | 2019 | NA | MSM | None | None | None |
| TSG-EHI7 | Nellore | 22 | M | ART Naïve | 134,388 | 318 | 2019 | NA | MSM | ND | M184I | ND |
| TSG-EHI9 | Nellore | 39 | M | ART Naïve | 104,713 | 439 | 2019 | NA | MSM | ND | M184I | ND |
| TSG-EHI28 | Nellore | 27 | M | ART Naïve | 1,40,000 | 301 | 2020 | NA | MSM | ND | M184I | ND |
| TSG-EHI32 | Nellore | 33 | M | ART Naïve | 505,000 | 147 | 2020 | NA | MSM | ND | M184I | ND |
| TSG-EHI38 | Nellore | 25 | M | ART Naïve | 105,000 | 349 | 2021 | NA | MSM | ND | NA | ND |
| TSG-EHI39 | Nellore | 46 | F | ART Naïve | 196,000 | 308 | 2021 | NA | HETEROSEXUAL | ND | M184I | ND |
| TSG-EHI41 | Nellore | 43 | M | ART Naïve | 304,297 | 89 | 2021 | NA | HETEROSEXUAL | ND | K219Q | ND |
| TSG-EHI42 | Nellore | 38 | M | ART Naïve | 262,078 | 123 | 2021 | NA | HETEROSEXUAL | ND | **K103N** | ND |
| TSG-EHI44 | Nellore | 33 | M | ART Naïve | 16,897 | 416 | 2021 | NA | HETEROSEXUAL | ND | M184I | ND |
| TSG-EHI45 | Nellore | 25 | M | ART Naïve | 140,651 | 525 | 2021 | NA | MSM | ND | M184I | ND |
| TSG-EHI47 | Nellore | 36 | M | ART Naïve | 19,817 | 647 | 2021 | NA | HETEROSEXUAL | ND | M184I | ND |
| TSG-EHI49 | Nellore | 33 | M | ART Naïve | 63,377 | 541 | 2021 | NA | MSM | None | None | None |
| TSG-EHI51 | Nellore | 48 | M | ART Naïve | 590,637 | 110 | 2021 | NA | HETEROSEXUAL | ND | M184I | ND |
| TSG-EHIPre8 | Nellore | ND | ND | ART Naïve | 414,557 |  | 2021 | NA | ND | ND | ND | ND |
| TSG-EHI17B14 | Nellore | 39 | M | ART Naïve | 25,625 | 268 | 2020 | NA | HETEROSEXUAL | None | M184I | None |
| TSG-EHI13D6 | Nellore | 28 | M | ART Naïve | 287,618 | 553 | 2020 | NA | HETEROSEXUAL | None | None | None |
| TSG-EHIPre15 | Nellore | ND | ND | ART Naïve | ND | ND | 2021 | NA | ND | ND | ND | ND |
| TSG-EHI8 | Nellore | 19 | M | ART Naïve | 77,078 | 154 | 2019 | NA | MSM | ND | Y181C | ND |
| TSG21N01N020 | Mumbai | 60 | M | ART Naïve | 37,98,480 | 415 | 2021 | NA | HETEROSEXUAL | ND | ND | ND |
| TSG21N01N021 | Mumbai | 55 | F | ART Naïve | Undetectable | 584 | 2021 | NA | HETEROSEXUAL | ND | ND | ND |
| TSG21N01N022 | Mumbai | 60 | F | ART Naïve | 138133 | 74 | 2021 | NA | HETEROSEXUAL | ND | ND | ND |
| TSG21N01S073CM | Mumbai | 53 | F | Receiving ART | Undetectable | 897 | 2021 | ND | HETEROSEXUAL | ND | ND | ND |
| TSG21N01S098 | Mumbai | 46 | F | Receiving ART | 17 | 538 | 2021 | ND | HETEROSEXUAL | ND | ND | ND |
| TSG21N01S102 | Mumbai | 41 | M | Receiving ART | 4472 | 1632 | 2021 | ND | HETEROSEXUAL | ND | ND | ND |
| TSG21N01S006 | Mumbai | 49 | M | Receiving ART | Undetectable | 759 | 2021 | ND | HETEROSEXUAL | ND | ND | ND |
| TSG21N01S007 | Mumbai | 51 | M | Receiving ART | 62 | 458 | 2021 | 2006 | HETEROSEXUAL | ND | ND | ND |
| TSG21N01S027 | Mumbai | 48 | M | Receiving ART | 1475 | 674 | 2021 | 2009 | HETEROSEXUAL | ND | ND | ND |
| TSG21N01S028 | Mumbai | 48 | M | Receiving ART | Undetectable | 1262 | 2021 | 2017 | HETEROSEXUAL | ND | ND | ND |
| TSG21S01A0013-STMART12 | Kolkata | 29 | M | ART Naïve | 29296 | ND | 2021 | NA | HETEROSEXUAL | ND | ND | ND |
| TSG21S01E0001-STM01E | Kolkata | 47 | M | ART Naïve | ND | ND | 2021 | NA | HETEROSEXUAL | ND | ND | ND |
| TSG21S01A0014-STM13ART | Kolkata | 31 | M | Receiving ART | 699 | ND | 2021 | 2008 | HETEROSEXUAL | ND | ND | ND |
| TSG21S01E0017-STM16E | Kolkata | 50 | F | ART Naïve | ND | ND | 2021 | NA | HETEROSEXUAL | ND | ND | ND |
| TSG21S01E0018-STM17E | Kolkata | 52 | M | ART Naïve | ND | ND | 2021 | NA | HETEROSEXUAL | ND | ND | ND |
| TSG21S01A0002-STMART01 | Kolkata | 20 | M | Receiving ART | ND | ND | 2021 | 2014 | HETEROSEXUAL | ND | ND | ND |
| TSG21S01A0003-STMART02 | Kolkata | 36 | F | Receiving ART | ND | ND | 2021 | 2011 | HETEROSEXUAL | ND | ND | ND |
| TSG21N01F003 | Mumbai | 37 | M | Receiving ART | 283195 | 9 | 2021 | ND | HETEROSEXUAL | ND | ND | ND |
| TSG21N01N013 | Mumbai | 45 | F | ART naïve | Undetectable | 172 | 2021 | NA | HETEROSEXUAL | ND | ND | ND |
| TSG21N01N014 | Mumbai | 53 | M | ART naïve | 131054 | 40 | 2021 | NA | HETEROSEXUAL | ND | ND | ND |
| TSG21N01N015 | Mumbai | 23 | M | ART naïve | 1444422 | 456 | 2021 | NA | HETEROSEXUAL | ND | ND | ND |
| TSG21N01N017 | Mumbai | 52 | F | ART naïve | 1927 | 1172 | 2021 | NA | HETEROSEXUAL | ND | ND | ND |
| TSG21N01N018 | Mumbai | 54 | M | ART naïve | 13,31,610 | 211 | 2021 | NA | HETEROSEXUAL | ND | ND | ND |
| TSG21N01N023 | Mumbai | 53 | M | ART naïve | 112 | 134 | 2021 | NA | HETEROSEXUAL | ND | ND | ND |
| TSG21N01N025 | Mumbai | 60 | M | ART naïve | 6558 | NA | 2021 | NA | HETEROSEXUAL | ND | ND | ND |
| TSG21N01N026 | Mumbai | 36 | M | ART naïve | 108614 | 125 | 2021 | NA | HETEROSEXUAL | ND | ND | ND |
| TSG21N01N028 | Mumbai | 52 | F | ART naïve | 380 | 355 | 2021 | NA | HETEROSEXUAL | ND | ND | ND |
| TSG21N01N031 | Mumbai | 31 | M | ART naïve | 356394 | 778 | 2021 | NA | HETEROSEXUAL | ND | ND | ND |
| TSG21N01N010 | Mumbai | 29 | M | ART naïve | 29506 | 213 | 2021 | NA | HETEROSEXUAL | ND | ND | ND |
| TSG21N01N001 | Mumbai | 50 | F | ART naïve | 1839 | 952 | 2021 | NA | HETEROSEXUAL | ND | ND | ND |
| TSG_IU-0016 | Mumbai | 41 | M | ART naïve | 328950 | 201 | 2021 | NA | HETEROSEXUAL | None | None | None |
| TSG_IU-0023 | Mumbai | 50 | F | ART naïve | 678124 | 63 | 2021 | NA | HETEROSEXUAL | None | None | None |
| TSG_IU-0024 | Mumbai | 44 | F | ART naïve | 163566 | 146 | 2021 | NA | HETEROSEXUAL | None | M184I | None |
| TSG_IU-0068 | Mumbai | 50 | F | ART naïve | 257156 | 90 | 2021 | NA | HETEROSEXUAL | None | None | None |
| TSG_IU-0036 | Mumbai | 59 | M | ART naïve | 63530 | 486 | 2021 | NA | HETEROSEXUAL | None | None | None |
| TSG_IU-0041 | Mumbai | 53 | M | ART naïve | 329635 | 277 | 2021 | NA | HETEROSEXUAL | None | M184I | None |
| TSG_IU-0044 | Mumbai | 24 | M | ART naïve | 26651 | 615 | 2021 | NA | HETEROSEXUAL | None | None | None |
| TSG_IU-0051 | Mumbai | 52 | F | ART naïve | 301915 | 166 | 2021 | NA | HETEROSEXUAL | None | **H221Y** | None |
| TSG_IU-0047 | Mumbai | 27 | M | ART naïve | 391914 | 617 | 2021 | NA | HETEROSEXUAL | ND | ND | ND |
| TSG_IU-0050 | Mumbai | 31 | M | ART naïve | 621950 | 72 | 2021 | NA | HETEROSEXUAL | ND | ND | ND |
| TSG_IU-0054 | Mumbai | 30 | M | ART naïve | 21035 | 599 | 2021 | NA | HETEROSEXUAL | None | **K103N** | R263K |
| TSG21N01S108 | Mumbai | 51 | M | Receiving ART | Undetectable | ND | 2021 | ND | HETEROSEXUAL | None | None | None |
| TSG21N01N036 | Mumbai | 33 | M | ART naïve | 92926 | 662 | 2021 | NA | HETEROSEXUAL | None | None | None |
| TSG21N01N039 | Mumbai | 37 | F | ART naïve | ND | 441 | 2021 | NA | HETEROSEXUAL | None | None | None |
| TSG21N01N040 | Mumbai | 45 | F | ART naïve | 4269 | 399 | 2021 | NA | HETEROSEXUAL | None | E138G, K65R | None |
| TSG21N01N041 | Mumbai | 40 | M | ART naïve | 61151 | ND | 2021 | NA | HETEROSEXUAL | None | None | None |
| TSG21N01N042 | Mumbai | 44 | M | ART naïve | ND | ND | 2021 | NA | HETEROSEXUAL | N83D | None | E138K |
| TSG21N01S105 | Mumbai | 27 | F | Receiving ART | Undetectable | 853 | 2021 | ND | HETEROSEXUAL | None | None | None |
| TSG21N01S092 | Mumbai | 35 | M | Receiving ART | Undetectable | 814 | 2021 | ND | HETEROSEXUAL | None | **M184I** | None |
| TSG21N01S088 | Mumbai | ND | M | Receiving ART | 129 | 818 | 2021 | ND | HETEROSEXUAL | M46I, D30N, **I85V** | **M184I** | None |
| TSG21N01N043 | Mumbai |  | M | ART naïve | Undetectable | ND | 2021 | NA | HETEROSEXUAL | None | None | None |
| TSG21N01N045 | Mumbai | 46 | M | ART naïve | 9367 | ND | 2021 | NA | HETEROSEXUAL | None | None | None |
| TSG21N01S038 | Mumbai | 43 | M | Receiving ART | Undetectable | 680 | 2021 | ND | HETEROSEXUAL | ND | ND | ND |
| TSG21N01F001 | Mumbai | 32 | M | Receiving ART | Undetectable | 25 | 2021 | ND | HETEROSEXUAL | ND | ND | ND |
| TSG21N01S104 | Mumbai | 48 | M | Receiving ART | Undetectable | 214 | 2021 | ND | HETEROSEXUAL | ND | ND | ND |
| TSG_IU-0057 | Mumbai | 50 | M | ART naïve | 2331565 | 127 | 2021 | NA | HETEROSEXUAL | None | None | None |
| TSG_IU-0037 | Mumbai | 43 | F | ART naïve | 562529 | 409 | 2021 | NA | HETEROSEXUAL | None | None | None |
| TSG_IU-0049 | Mumbai | 37 | M | ART naïve | 159237 | 605 | 2021 | NA | HETEROSEXUAL | None | None | None |
| TSG_IU-0026 | Mumbai | 34 | M | ART naïve | 97122 | 168 | 2021 | NA | HETEROSEXUAL | **M46I** | **E138G** | None |
| TSG_IU-0034 | Mumbai | 50 | F | ART naïve | 372086 | 123 | 2021 | NA | HETEROSEXUAL | None | None | None |
| TSG_IU-0019 | Mumbai | 40 | M | ART naïve | 217655 | 343 | 2021 | NA | HETEROSEXUAL | **I47V** | None | None |
| TSG_IU-0045 | Mumbai | 52 | F | ART naïve | 75238 | 468 | 2021 | NA | HETEROSEXUAL | None | None | None |
| TSG_IU-0052 | Mumbai | 41 | M | ART naïve | 700909 | 52 | 2021 | NA | HETEROSEXUAL | ND | ND | ND |
| TSG21N01N002 | Mumbai | 46 | M | ART naïve | 15511 | 108 | 2021 | NA | HETEROSEXUAL | None | None | None |
| TSG21N01S030 | Mumbai | 40-45 | F | Receiving ART | Undetectable | 1436 | 2021 | 2004 | HETEROSEXUAL | ND | ND | ND |
| TSG21N01S095 | Mumbai | 32 | F | Receiving ART | 31 | 1008 | 2021 | ND | HETEROSEXUAL | ND | ND | ND |
| TSG21N01S100 | Mumbai | 21 | F | Receiving ART | ND | 1103 | 2021 | 2010 | HETEROSEXUAL | ND | ND | ND |
| TSG21N01S055 | Mumbai | 47 | M | Receiving ART | Undetectable | 810 | 2021 | 2005 | HETEROSEXUAL | ND | ND | ND |
| TSG21N01S062 | Mumbai | 33 | F | Receiving ART | Undetectable | 715 | 2021 | ND | HETEROSEXUAL | ND | ND | ND |
| TSG21N01N003 | Mumbai | 29 | M | ART naïve | 427864 | 266 | 2021 | NA | HETEROSEXUAL | None | None | None |
| TSG23Y07A0012-A12_Aizawl | Aizawl | ND | ND | Receiving ART | Undetectable | 472 | 2023 | 2018 | MSM | **M46I** | **M184I** | ND |
| TSG23Y07A0013-A13_Aizawl | Aizawl | ND | ND | Receiving ART | Undetectable | 105 | 2023 | 2020 | MSM | ND | F77L | ND |
| TSG23Y07A0015-A15_Aizawl | Aizawl | ND | ND | ART naïve | 76500 | 325 | 2023 | NA | MSM | ND | ND | E92G |
| TSG23Y07E0015-A3_Aizawl | Aizawl | ND | ND | Receiving ART | 42 | 577 | 2023 | 2021 | MSM | I54T | ND | ND |
| TSG22Y07A0005-A5_Aizawl | Aizawl | ND | ND | Receiving ART | <20 | 1104 | 2022 | 2020 | MSM | ND | D67E | ND |
| TSG22Y05A0013-BL13 | Belagavi | 38 | M | Receiving ART | 39 | 354 | 2022 | 2020 | MSM | ND | E138A | ND |
| TSG22Y05A0014-BL14 | Belagavi | 45 | M | Receiving ART | 39 | 404 | 2022 | 2018 | MSM | ND | ND | ND |
| TSG22Y05A0017-BL17 | Belagavi | 36 | M | Receiving ART | 16820 | 329 | 2022 | 2016 | MSM | ND | F77L | ND |
| TSG22Y05A0018-BL18 | Belagavi | 35 | M | Receiving ART | 39 | 385 | 2022 | 2017 | MSM | ND | ND | E92G |
| TSG22Y05A0001-BL1 | Belagavi | 41 | M | Receiving ART | Undetectable | 419 | 2022 | 2018 | MSM | ND | M184I | ND |
| TSG22Y05E0020-BL20 | Belagavi | 27 | M | ART Naïve | 87134 | 45 | 2022 | NA | MSM | None | None | ND |
| TSG22Y05A0021-BL21 | Belagavi | 24 | M | Receiving ART | 25271 | 487 | 2022 | 2020 | MSM | ND | M230I | ND |
| TSG22Y05A0024-BL24 | Belagavi | 30 | M | Receiving ART | 35036 | 468 | 2022 | 2012 | MSM | ND | M46I | ND |
| TSG22Y05A0026-BL26 | Belagavi | 45 | M | Receiving ART | 24649 | 390 | 2022 | 2018 | MSM | ND | M184I | ND |
| TSG22Y05A0028-BL28 | Belagavi | 45 | M | Receiving ART | 39 | 570 | 2022 | 2017 | MSM | ND | M230I | ND |
| TSG22Y05A0029-BL29 | Belagavi | 36 | M | Receiving ART | 5695 | 399 | 2022 | 2018 | MSM | ND | M230I | ND |
| TSG22Y05A0002-BL2 | Belagavi | 28 | M | Receiving ART | Undetectable | 625 | 2022 | 2021 | MSM | ND | ND | **R263K** |
| TSG22Y05E0030-BL30 | Belagavi | 33 | M | ART Naïve | 39454 | 339 | 2022 | NA | MSM | **Q58E** | ND | ND |
| TSG22Y05E0033-BL33 | Belagavi | 29 | M | ART Naïve | 2981 | 500 | 2022 | NA | MSM | ND | M230I | ND |
| TSG22Y05A0034-BL34 | Belagavi | 19 | M | Receiving ART | 203692 | 106 | 2022 | 2022 | MSM | ND | **E138A** | ND |
| TSG22Y05E0036-BL36 | Belagavi | 44 | M | ART Naïve | 4051827 | 34 | 2022 | NA | MSM | ND | M230I | ND |
| TSG22Y05A0037-BL37 | Belagavi | 43 | M | Receiving ART | 164219 | 362 | 2022 | 2018 | MSM | ND | ND | ND |
| TSG22Y05E0038-BL38 | Belagavi | 45 | M | ART Naïve | 560516 | 200 | 2022 | NA | MSM | None | None | None |
| TSG22Y05E0039-BL39 | Belagavi | 24 | M | ART Naïve | 4553 | 324 | 2022 | NA | MSM | I54V | NA | ND |
| TSG22Y05A0003-BL3 | Belagavi | 36 | M | Receiving ART | Undetectable | 261 | 2022 | 2010 | MSM | ND | **M184V** | ND |
| TSG22Y05E0041-BL41 | Belagavi | 23 | M | ART Naïve | 859639 | 632 | 2022 | NA | MSM | ND | **K103N** | ND |
| TSG22Y05E0043-BL43 | Belagavi | 42 | M | ART Naïve | 39 | 142 | 2022 | NA | MSM | T74P | ND | ND |
| TSG22Y05E0045-BL45 | Belagavi | 45 | M | ART Naïve | 377546 | 99 | 2022 | NA | MSM | None | None | None |
| TSG22Y05E0046-BL46 | Belagavi | 38 | F | ART Naïve | 34223 | 135 | 2022 | NA | HETEROSEXUAL | ND | D67E | ND |
| TSG22Y05E0047-BL47 | Belagavi | 35 | M | ART Naïve | 193853 | 90 | 2022 | NA | MSM | ND | ND | ND |
| TSG22Y05E0048-BL48 | Belagavi | 38 | M | ART Naïve | 219784 | 287 | 2022 | NA | MSM | ND | M230I | ND |
| TSG22Y05A0004-BL4 | Belagavi | 45 | M | Receiving ART | Undetectable | 556 | 2022 | 2010 | MSM | ND | M184I | ND |
| TSG22Y05A0006-BL6 | Belagavi | 28 | M | Receiving ART | Undetectable | 206 | 2022 | 2016 | MSM | ND | V106A | ND |
| TSG22Y05A0007-BL7 | Belagavi | 34 | M | Receiving ART | 2235 | 569 | 2022 | 2018 | MSM | ND | P225H | ND |
| TSG22Y04A0011-H11 | Hyderabad | 35 | M | Receiving ART | Undetectable | 411 | 2022 | 2019 | MSM | ND | M230I | ND |
| TSG22Y04A0013-H13 | Hyderabad | 40 | M | Receiving ART | Undetectable | 1228 | 2022 | 2018 | MSM | ND | K70R | ND |
| TSG22Y04A0015-H15 | Hyderabad | 36 | M | Receiving ART | Undetectable | 1107 | 2022 | 2014 | MSM | ND | L100I | ND |
| TSG22Y04A0016-H16 | Hyderabad | 34 | M | Receiving ART | 1915 | 614 | 2022 | 2015 | MSM | ND | M230I | ND |
| TSG22Y04A0001-H1 | Hyderabad | 28 | M | Receiving ART | Undetectable | 838 | 2022 | 2019 | MSM | None | None | None |
| TSG22Y04A0020-H20 | Hyderabad | 28 | M | Receiving ART | Undetectable | 560 | 2022 | 2017 | MSM | ND | Y188H | ND |
| TSG22Y04A0002-H2 | Hyderabad | 31 | M | Receiving ART | Undetectable | 619 | 2022 | 2018 | MSM | None | None | None |
| TSG22Y04E0031-H31 | Hyderabad | 24 | M | ART Naïve | 21038 | 405 | 2022 | NA | MSM | None | None | None |
| TSG22Y04A0003-H3 | Hyderabad | 40 | M | Receiving ART | Undetectable | 936 | 2022 | 2017 | MSM | ND | D67N | ND |
| TSG22Y04A0004-H4 | Hyderabad | 44 | M | Receiving ART | Undetectable | 505 | 2022 | 2011 | MSM | ND | M184I | ND |
| TSG22Y04A0005-H5 | Hyderabad | 41 | F | Receiving ART | 1993 | 463 | 2022 | 2011 | HETEROSEXUAL | ND | G190S | ND |
| TSG22Y04A0006-H6 | Hyderabad | 27 | M | Receiving ART | Undetectable | 839 | 2022 | 2019 | MSM | ND | ND | ND |
| TSG22Y04A0007-H7 | Hyderabad | 31 | M | Receiving ART | 17631 | 1104 | 2022 | 2019 | MSM | None | None | None |
| TSG22Y04A0009-H9 | Hyderabad | 42 | M | Receiving ART | Undetectable | 1355 | 2022 | 2015 | MSM | ND | E138G | NA |
| TSG23Y06A012-V12 | Vizag | 44 | M | Receiving ART | Undetectable | 640 | 2022 | 2021 | MSM | None | None | None |
| TSG23Y06A013-V13 | Vizag | ND | ND | Receiving ART | ND | ND | 2023 | ND | MSM | ND | M230I | ND |
| TSG23Y06E014-V14 | Vizag | 29 | M | ART Naïve | 15000 | 434 | 2023 | NA | MSM | ND | M230I | ND |
| TSG23Y06A015-V15 | Vizag | 26 | M | Receiving ART | Undetectable | 223 | 2023 | 2022 | MSM | ND | K70R | ND |
| TSG23Y06E001-V1 | Vizag | 42 | M | ART Naïve | Undetectable | 406 | 2023 | NA | MSM | ND | D67E | ND |
| TSG23Y06A003-V3 | Vizag | 42 | M | Receiving ART | Undetectable | 459 | 2023 | 2023 | MSM | ND | M230I | ND |
| TSG23Y06A004-V4 | Vizag | 23 | M | Receiving ART | Undetectable | 470 | 2023 | 2023 | MSM | ND | G190S | ND |
| TSG23Y06E005-V5 | Vizag | 39 | M | ART Naïve | 14000 | 346 | 2023 | NA | MSM | ND | ND | ND |
| TSG-EHIPre14 | Nellore | ND | ND | ART Naïve | 81,714 | ND | 2018 | NA | ND | ND | M184I | ND |
| TSG-EHI25 | Nellore | 44 | M | ART Naïve | 99,400 | 226 | 2020 | NA | HETEROSEXUAL | None | M184I | None |
| TSG-EHI34 | Nellore | ND | ND | ART Naïve | 22,800 | ND | 2020 | NA | ND | ND | M184I | ND |
| TSG-EHI36 | Nellore | ND | ND | ART Naïve | 1,97,800 | ND | 2020 | NA | ND | ND | M184I | ND |
| TSG-EHI50 | Nellore | ND | ND | ART Naïve | ND | 444 | 2021 | NA | MSM | ND | M184I | ND |
| TSG-EHI53 | Nellore | ND | ND | ART Naïve | 7170 | 324 | 2022 | NA | MSM | ND | M184I | ND |
| TSG-EHI55 | Nellore | ND | ND | ART Naïve | 23200 | 355 | 2022 | NA | MSM | ND | None | ND |
| TSG-EHI57 | Nellore | ND | ND | ART Naïve | 29300 | 503 | 2022 | NA | MSM | None | None | None |
| TSG-EHI58 | Nellore | ND | ND | ART Naïve | 39400 | 915 | 2022 | NA | MSM | ND | M184I | ND |
| TSG-EHI59 | Nellore | ND | ND | ART Naïve | 31900 | 356 | 2022 | NA | MSM | None | None | None |
| TSG-EHI60 | Nellore | ND | ND | ART Naïve | 22800 | 448 | 2022 | NA | MSM | None | None | None |
| TSG-EHI61 | Nellore | ND | ND | ART Naïve | 32100 | 389 | 2022 | NA | MSM | None | None | None |
| TSG-EHI62 | Nellore | ND | ND | ART Naïve | 126000 | 514 | 2022 | NA | MSM | ND | M184I | ND |
| TSG-EHI63 | Nellore | ND | ND | ART Naïve | 6800 | 330 | 2022 | NA | MSM | None | None | None |
| TSG-EHI14 | Nellore | 35 | M | ART Naïve | 642,486 | 49 | 2020 | NA | ND | None | M184I, G190S | None |
| TSG-EHI40-C18 | Nellore | 41 | M | ART Naïve | 698,000 | 400 | 2021 | NA | ND | ND | M184I | ND |
| TSG21N01N011-C10 | Mumbai | 30 | M | ART naïve | 269097 | 690 | 2021 | NA | HETEROSEXUAL | ND | ND | ND |
| TSG21N01N029 | Mumbai | ND | M | ART naïve | 184797 | 295 | 2021 | NA | HETEROSEXUAL | ND | ND | ND |
| TSG21Y02A0012 | Delhi | 25 | M | Receiving ART | 60062.9 | 153.0 | 2021 | ND | MSM | ND | ND | ND |
| TSG21S01A0005-C5 | Kolkata | 33 | M | Receiving ART | ND | ND | 2021 | 2009 | HETEROSEXUAL | ND | ND | ND |

**Key:**

| NA | : Not Applicable |
| --- | --- |
| ND | : No data |
| Bold DRMs | : Frequency >10% |
