# Supplementary material for: Distinct region-specific neutralization profiles of contemporary HIV-1 clade C against best-in-class broadly neutralizing antibodies
Source: J Virol. 2025 May 16;99(6):e00008-25. doi: 10.1128/jvi.00008-25 (PMC7617755; doi:10.1128/jvi.00008-25)
Supplement: Table S2 — Neutralization breadth and potency (IC50) of pseudoviruses expressing contemporary India clade C envs. [file jvi.00008-25-s0010.pdf]

Table S2. Neutralization breadth and potency (IC50 and IC80) of pseudoviruses expressing contemporary India clade C *envs*.

IC50 values (μg/mL)

Viruses	CAP256-VRC26.25	PGDM1400	PG9	PGT145	VRC01	VRC07	1-18	N6	3BNC117	PGT121	10-1074	BG-18	10E8	VRC34.01
TSG-EHI6	>25	0.04	0.05	>25	0.26	0.22	<0.01	0.02	0.02	1.25	0.13	0.09	>25	>25
TSG-EHI9	>25	>25	4.68	>25	0.03	0.02	<0.01	0.02	<0.01	9.37	0.03	>25	>25	>25
TSG-EHI28	1.03	2.30	0.32	0.56	>25	0.34	0.01	0.01	>25	2.53	0.03	<0.01	5.82	0.63
TSG-EHI32	>25	>25	0.05	>25	0.07	<0.01	0.13	0.02	0.02	1.40	<0.01	<0.01	>25	0.52
TSG-EHI38	<0.01	0.02	>25	0.01	0.61	0.03	<0.01	>25	>25	0.62	0.02	<0.01	1.18	0.50
TSG-EHI39	<0.01	<0.01	0.34	2.14	1.01	0.08	0.12	0.36	1.61	0.77	1.13	7.74	3.96	0.10
TSG-EHI41	0.31	>25	>25	>25	1.61	0.18	0.37	0.35	>25	6.38	0.07	<0.01	>25	>25
TSG-EHI42	<0.01	<0.01	7.36	3.74	<0.01	<0.01	<0.01	0.04	0.11	6.77	0.20	0.11	1.11	>25
TSG-EHI44	>25	>25	>25	>25	>25	9.24	<0.01	0.01	11.75	5.83	0.05	<0.01	7.81	>25
TSG-EHI45	<0.01	2.26	>25	>25	2.17	0.24	0.18	1.40	2.66	0.23	1.43	1.95	19.82	0.11
TSG-EHIPre8	>25	>25	0.03	>25	0.08	0.15	1.12	0.01	0.02	0.29	0.40	4.15	>25	>25
TSG-EHI13D6	>25	0.10	>25	>25	0.76	0.03	>25	0.03	0.02	>25	0.02	0.56	>25	>25
TSG-EHI17B14	>25	>25	0.28	>25	>25	>25	0.04	0.23	3.09	0.12	<0.01	0.08	>25	>25
TSG-EHI8	>25	0.18	0.10	0.20	0.21	<0.01	1.16	<0.01	>25	>25	4.83	0.10	4.47	14.35
TSG-EHI14	<0.01	<0.01	0.19	0.01	0.16	0.01	<0.01	<0.01	0.23	<0.01	<0.01	<0.01	0.06	0.17
TSG-EHI40-C18	<0.01	0.04	0.02	0.06	0.64	0.10	<0.01	0.11	0.18	0.01	0.04	0.16	0.40	<0.01
TSG-EHI11	>25	0.19	0.51	2.80	0.36	0.15	0.15	0.10	0.11	0.36	0.05	<0.01	>25	>25
TSG-EHI21	>25	0.02	0.03	0.02	8.08	0.06	0.05	0.02	0.62	0.10	0.02	<0.01	0.64	0.23
TSG-EHI26	>25	12.42	5.66	>25	0.33	0.08	0.05	<0.01	<0.01	>25	0.05	0.13	0.26	0.12
TSG-EHI30	>25	0.46	1.25	>25	0.02	<0.01	0.17	0.07	0.07	0.11	0.05	<0.01	1.35	1.72
TSG-EHI18	>25	0.27	0.01	<0.01	0.15	0.05	0.01	0.05	0.01	0.06	0.02	<0.01	0.23	0.13
TSG-EHI22	>25	0.02	0.16	1.26	1.51	0.10	0.03	0.34	>25	0.07	<0.01	<0.01	>25	>25
TSG-EHI29	>25	1.06	0.19	>25	0.79	0.67	3.15	0.15	0.26	0.03	>25	>25	1.62	1.86
TSG-EHI35	>25	0.01	0.08	0.83	0.05	<0.01	<0.01	<0.01	>25	>25	2.41	1.37	0.80	>25
TSG-EHI37	>25	>25	>25	>25	0.43	0.13	0.12	0.11	0.32	>25	0.09	<0.01	0.56	>25
TSG-EHI7	>25	>25	>25	>25	3.91	0.08	0.14	0.67	>25	0.61	0.15	0.17	>25	0.27
TSG-EHI12	>25	0.07	0.37	>25	0.07	0.03	0.01	0.03	0.02	3.35	0.02	<0.01	1.18	>25
TSG-EHI20	>25	>25	>25	>25	0.09	0.03	0.30	0.02	0.02	>25	>25	>25	1.81	1.19
TSG-EHI23	<0.01	0.01	>25	>25	0.24	0.04	0.59	0.09	1.87	2.83	0.09	<0.01	1.54	>25
TSG-EHI27	>25	>25	>25	>25	>25	>25	0.35	0.19	>25	>25	>25	0.15	0.66	0.14
TSG-EHI33	<0.01	<0.01	0.14	2.61	<0.01	<0.01	0.12	<0.01	>25	0.23	0.05	0.11	>25	>25
TSG-EHI55	>25	>25	>25	>25	0.19	0.09	0.03	0.12	0.04	2.29	0.45	0.03	1.65	0.04
TSG-EHI60	>25	>25	>25	>25	0.03	0.06	0.04	0.02	0.10	>25	>25	>25	3.38	0.08
TSG-EHI61	0.36	>25	0.08	>25	>25	>25	0.53	>25	>25	<0.01	<0.01	<0.01	0.49	>25
TSG-EHI62	>25	3.24	<0.01	>25	1.11	0.14	0.04	<0.01	0.70	>25	0.28	>25	<0.01	<0.01
TSG-EHI63	>25	>25	<0.01	>25	1.61	0.47	0.17	0.02	0.27	0.47	>25	>25	1.89	>25
TSG-EHI25	0.01	1.38	>25	>25	7.58	0.71	0.73	0.39	7.03	0.05	0.76	>25	1.28	>25
TSG-EHI50	>25	>25	>25	>25	4.20	1.53	0.48	0.38	0.08	0.05	>25	>25	<0.01	1.98
TSG-EHIPre15	0.07	>25	0.31	0.54	>25	1.20	1.11	2.12	>25	0.12	0.18	<0.01	4.15	>25
TSG-EHI51	0.03	>25	>25	20.59	0.16	0.02	0.09	0.06	0.31	3.23	0.93	13.39	0.62	2.81
TSG-EHI16	0.15	4.28	0.19	>25	0.03	<0.01	0.10	<0.01	<0.01	0.06	0.01	<0.01	2.59	0.04
TSG-EHI36	1.58	<0.01	12.71	2.19	0.02	0.13	>25	0.12	>25	>25	>25	>25	0.71	>25
TSG-EHI34	>25	>25	>25	>25	0.51	0.11	0.07	0.05	0.34	0.08	<0.01	<0.01	4.30	0.19
TSG-EHI53	<0.01	<0.01	0.05	<0.01	0.39	0.17	0.03	0.05	0.21	>25	>25	>25	0.25	>25
TSG-EHI57	1.47	>25	>25	>25	>25	4.69	0.80	0.50	>25	0.01	0.02	<0.01	2.01	1.66

Table S2. Neutralization breadth and potency (IC50 and IC80) of pseudoviruses expressing contemporary India clade C *envs.*

Viruses	CAP256-VRC26.25	PGDM1400	PG9	PGT145	VRC01	VRC07	1-18	N6	3BNC117	PGT121	10-1074	BG-18	10E8	VRC34.01
TSG-EHI58	0.01	>25	>25	>25	0.63	0.11	0.09	0.15	0.22	>25	6.23	4.87	0.14	>25
TSG-EHI59	12.04	>25	0.14	0.02	>25	>25	2.39	>25	>25	0.03	0.02	<0.01	0.59	0.01
TSG21Y02A0012	>25	>25	0.21	0.74	0.28	0.04	<0.01	0.08	>25	0.02	0.01	<0.01	9.35	0.06
TSG21Y02E0018-D18	0.41	1.97	>25	0.33	0.38	0.02	0.54	<0.01	4.89	0.04	0.30	<0.01	>25	0.06
TSG21Y02E0024-D24	<0.01	<0.01	<0.01	9.18	0.05	0.13	0.15	0.01	0.13	11.58	>25	>25	0.45	>25
TSG22Y02E0035-DE35	<0.01	1.10	14.03	0.80	>25	5.13	0.54	1.81	13.03	0.36	0.03	0.02	2.63	0.58
TSG22Y02E0037-DE37	>25	>25	4.27	>25	0.60	0.23	0.08	<0.01	>25	0.12	0.03	<0.01	6.16	0.32
TSG21Y02E0011-D11	>25	>25	0.44	>25	0.23	0.33	0.64	0.21	2.74	0.10	0.08	0.02	9.82	0.05
TSG21Y02E0014-D14	<0.0	<0.01	0.02	<0.01	0.04	0.01	0.04	0.02	0.31	0.14	0.08	<0.01	0.47	0.15
TSG21S01A0008_STMART07	0.11	0.61	>25	>25	1.50	0.15	0.03	0.37	0.49	0.20	6.73	3.73	1.97	0.87
TSG21S01A0013-STMART12	>25	1.64	0.23	>25	2.24	0.56	0.40	1.40	0.30	0.07	0.05	0.05	10.38	0.04
TSG21S01A0003-STMART02	>25	4.48	0.02	>25	2.29	0.09	0.76	1.12	7.47	0.25	0.11	<0.01	1.11	
TSG21SO1A0014-STM13ART	>25	>25	1.40	>25	3.75	0.52	0.11	0.94	1.17	1.57	1.06	0.26	>25	0.20
TSG21S01E0017-STM16E	<0.01	0.58	4.65	0.23	2.25	0.75	0.08	0.29	3.01	0.47	0.12	<0.01	4.03	>25
TSG21S01E0001-STM01E	<0.01	<0.01	>25	>25	1.64	0.50	<0.01	0.09	>25	1.06	0.03	<0.01	3.52	>25
TSG21S01A0002-STMART01	>25	>25	3.72	17.09	9.01	0.53	0.36	3.43	3.81	1.06	0.03	<0.01	0.34	>25
TSG21S01E0018-STM17E	<0.01	>25	>25	>25	1.26	0.27	0.04	0.22	2.98	0.48	0.14	0.01	1.28	0.01
TSG21S01A0004-C4	>25	>25	>25	>25	1.20	0.10	0.24	0.09	0.27	0.05	0.25	<0.01	6.04	0.07
TSG21S01A0005-C5	<0.01	<0.01	0.04	0.04	2.27	0.20	0.23	0.13	2.73	0.01	<0.01	<0.01	0.02	0.48
TSG21S01E0033-C33	<0.01	8.02	<0.01	>25	>25	>25	<0.01	0.01	>25	0.11	0.01	<0.01	0.44	>25
TSG21N01N017_C18	>25	>25	>25	7.17	>25	18.38	>25	>25	>25	8.17	0.24	>25	7.83	11.66
TSG21N01N011-C10	<0.01	0.07	<0.01	0.å40	0.22	0.03	0.04	0.19	0.06	0.40	0.07	<0.01	0.07	>25
TSG21N01N029	<0.01	0.04	0.10	<0.01	0.18	0.27	1.50	0.10	1.15	0.41	0.05	0.77	0.19	0.24
TSG21N01N018	>25	10.58	>25	>25	0.56	0.20	13.41	0.21	0.49	0.05	0.01	<0.01	0.11	2.47
TSG21N01S028	>25	>25	>25	>25	0.16	0.02	0.15	0.03	8.27	11.13	0.06	<0.01	1.63	>25
TSG21N01N010	7.98	<0.01	0.03	0.28	5.55	0.72	0.49	0.58	>25	<0.01	<0.01	<0.01	0.40	<0.01
TSG21N01N014	>25	>25	>25	>25	3.23	0.88	0.22	0.50	4.57	1.07	>25	>25	5.22	0.16
TSG21N01N023	>25	>25	>25	>25	0.29	0.04	<0.01	0.09	0.27	>25	>25	>25	4.01	0.19
TSG21N01N031	>25	>25	>25	>25	0.26	0.04	0.01	0.04	>25	0.07	15.14	>25	1.46	>25
TSG21N01F003	0.16	>25	>25	>25	0.70	0.24	0.17	0.23	0.36	1.32	>25	>25	3.45	>25
TSG21N01N001CM	<0.01	<0.01	0.05	0.27	5.39	0.09	0.20	0.02	>25	12.62	3.31	6.27	0.06	<0.01
TSG21N01N013	<0.01	0.30	>25	>25	1.36	0.16	0.21	0.37	0.72	>25	0.08	<0.01	>25	18.01
TSG21N01N015	0.04	<0.01	0.73	2.30	0.20	0.02	0.04	<0.01	1.39	<0.01	<0.01	<0.01	0.82	3.22
TSG21N01N025	>25	<0.01	5.20	4.58	10.62	6.02	0.04	1.70	>25	12.26	0.09	>25	<0.01	0.03
TSG21N01N026	>25	<0.01	<0.01	1.58	2.82	0.48	0.09	0.24	0.76	0.23	0.16	0.03	<0.01	>25
TSG21N01S007	>25	>25	>25	>25	1.07	0.20	4.25	1.42	>25	0.81	0.62	0.01	2.78	0.90
TSG21N01S027	<0.01	0.02	>25	0.03	0.06	<0.01	0.07	0.09	>25	9.54	>25	>25	0.44	1.21
TSG21N01S030	10.96	>25	>25	>25	>25	0.94	2.89	0.48	5.29	0.25	0.68	0.04	3.11	0.12
TSG21N01S100	<0.01	>25	0.01	<0.01	0.93	0.16	0.35	0.03	0.22	0.05	0.05	0.02	0.43	0.17
TSG21N01S055	<0.01	>25	0.38	0.25	4.19	1.08	1.64	0.59	1.50	>25	>25	>25	0.23	0.04
TSG21N01S062	<0.01	>25	10.59	0.10	0.18	0.07	0.16	0.13	>25	1.03	0.23	0.02	1.05	1.92
TSG22Y03E0010-B10	<0.01	<0.01	0.09	0.18	0.02	0.01	0.05	0.02	0.08	<0.01	0.02	<0.01	0.84	0.08
TSG22Y03A0015-B15	<0.01	<0.01	0.54	0.41	0.03	0.06	<0.01	<0.01	6.75	<0.01	0.01	<0.01	2.48	0.04
TSG22Y03A0022-B22	<0.01	<0.01	6.07	3.28	0.02	0.04	0.08	<0.01	0.57	<0.01	<0.01	<0.01	0.89	0.06
TSG22Y03A0031-B31	<0.01	<0.01	0.11	0.35	9.12	0.07	<0.01	<0.01	>25	0.05	0.03	<0.01	0.60	0.04
TSG22Y03A0032-B32	<0.01	0.02	0.53	0.29	0.02	0.03	0.05	0.01	0.12	0.22	0.10	0.02	1.92	0.16

Table S2. Neutralization breadth and potency (IC50 and IC80) of pseudoviruses expressing contemporary India clade C *envs.*

Viruses	CAP256-VRC26.25	PGDM1400	PG9	PGT145	VRC01	VRC07	1-18	N6	3BNC117	PGT121	10-1074	BG-18	10E8	VRC34.01
TSG22Y03E0019-B19	>25	>25	>25	5.02	0.46	0.09	0.17	<0.01	0.31	<0.01	<0.01	<0.01	0.60	5.73
TSG22Y03E0023-B23	0.05	0.25	5.45	0.02	>25	4.82	>25	6.51	9.57	0.08	0.09	0.01	<0.01	>25
TSG23Y07A0012-A12	<0.01	1.98	>25	0.51	5.16	0.41	0.23	0.15	0.74	>25	>25	>25	3.81	>25
TSG23Y07A0013-A13	7.80	0.01	0.54	0.02	0.16	0.08	0.10	0.05	0.36	0.09	0.02	<0.01	0.58	>25
TSG23Y07A0015-A15	>25	<0.01	1.46	0.14	>25	0.02	0.20	<0.01	>25	0.15	0.10	<0.01	0.55	4.34
TSG23Y06A012-V12	<0.01	0.02	0.01	0.36	16.91	0.11	>25	1.77	0.05	0.22	0.04	0.04	0.01	>25
TSG23Y06A015-V15	<0.01	<0.01	0.08	0.47	3.55	0.57	1.09	0.42	>25	0.23	0.05	<0.01	0.10	0.06
TSG23Y06E001-V1	>25	0.31	0.36	0.68	0.17	0.11	0.11	0.06	2.63	0.05	0.03	<0.01	1.81	>25
TSG23Y06A003-V3	9.95	<0.01	0.02	0.04	0.62	0.28	0.09	0.15	0.40	0.08	0.02	<0.01	0.18	0.04
TSG23Y06A004-V4	<0.01	>25	0.04	>25	0.68	0.40	14.25	0.27	>25	<0.01	0.03	<0.01	>25	>25
TSG22Y04A0011-H11	>25	>25	0.75	0.18	0.54	0.14	0.11	0.16	0.57	0.82	0.29	1.36	5.89	>25
TSG22Y04A0015-H15	0.02	0.33	0.09	0.06	1.24	0.54	0.36	0.34	0.43	0.82	0.29	0.09	1.50	0.06
TSG22Y04A0016-H16	0.02	<0.01	0.04	<0.01	>25	0.04	0.04	0.02	>25	0.37	0.20	>25	>25	0.32
TSG22Y04A0020-H20	6.32	<0.01	0.03	<0.01	<0.01	<0.01	<0.01	<0.01	0.03	<0.01	<0.01	<0.01	0.44	>25
TSG22Y04A0002-H2	<0.01	0.02	0.41	0.22	2.95	0.32	1.53	0.15	0.16	>25	8.59	2.74	0.13	>25
TSG22Y04A0003-H3	<0.01	<0.01	<0.01	1.34	<0.01	<0.01	0.02	<0.01	<0.01	0.04	1.98	>25	0.02	0.03
TSG22Y04A0004-H4	>25	10.23	>25	0.52	>25	1.78	1.68	1.19	>25	0.04	0.06	0.06	14.57	>25
TSG22Y04A0005-H5	<0.01	0.40	0.18	0.08	3.76	0.89	0.18	0.47	24.12	0.08	0.06	<0.01	3.30	0.16
TSG22Y05A0018-BL18	>25	1.27	>25	0.07	>25	>25	>25	>25	>25	>25	>25	>25	1.99	>25
TSG22Y05A0021-BL21	>25	15.24	0.81	>25	0.26	0.12	0.06	0.12	>25	0.42	0.05	<0.01	13.02	>25
TSG22Y05A0002-BL2	<0.01	>25	0.02	11.67	0.15	0.09	2.44	0.07	0.15	0.51	0.20	<0.01	7.85	>25
TSG22Y05E0033-BL33	>25	>25	>25	>25	0.34	0.06	0.16	0.05	>25	0.05	0.04	0.02	1.52	>25
TSG22Y05E0043-BL43	<0.01	<0.01	0.03	<0.01	4.97	1.60	0.45	1.18	3.18	0.56	0.10	<0.01	2.13	>25
TSG22Y05A0006-BL6	2.54	>25	0.42	>25	>25	>25	>25	>25	>25	<0.01	<0.01	<0.01	0.46	<0.01

The values indicate neutralization titers (μg/mL) that conferred 50% pseudovirus neutralization in TZM-bl cells.

Table S2. Neutralization breadth and potency (IC50 and IC80) of pseudoviruses expressing contemporary India clade C *envs.*

IC80 values (μg/mL)

Viruses	CAP256-VRC26.25	PGDM1400	PG9	PGT145	VRC01	VRC07	1-18	N6	3BNC117	PGT121	10-1074	BG-18	10E8	VRC34.01
TSG-EHI6	>25	0.17	0.16	>25	0.97	0.67	0.05	0.10	0.11	>25	0.22	0.21	>25	>25
TSG-EHI9	>25	>25	>25	>25	0.16	0.07	<0.01	0.05	0.03	19.55	0.16	>25	>25	>25
TSG-EHI28	>25	5.12	1.33	4.31	>25	0.65	0.04	0.03	>25	6.31	0.13	0.03	18.78	1.96
TSG-EHI32	>25	>25	0.18	>25	0.26	0.28	12.34	0.06	0.09	9.65	1.67	<0.01	>25	2.06
TSG-EHI38	<0.01	0.14	>25	0.18	1.62	0.13	0.03	>25	>25	4.45	0.08	<0.01	3.67	>25
TSG-EHI39	<0.01	0.03	5.37	24.95	2.77	0.28	0.61	1.23	3.98	>25	3.06	>25	8.11	0.35
TSG-EHI41	20.91	>25	>25	>25	2.89	0.48	1.91	1.33	>25	12.44	0.29	<0.01	>25	>25
TSG-EHI42	0.03	0.20	19.37	12.19	0.32	0.09	<0.01	0.07	0.32	>25	2.27	3.72	3.47	>25
TSG-EHI44	>25	>25	>25	>25	>25	>25	0.02	0.05	>25	12.39	0.23	0.02	25.06	>25
TSG-EHI45	1.01	>25	>25	>25	0.85	2.78	0.60	9.57	12.05	>25	4.23	>25	>25	1.91
TSG-EHIPre8	>25	>25	4.58	>25	1.73	2.41	>25	0.08	0.13	4.99	12.99	>25	>25	>25
TSG-EHI13D6	>25	0.45	>25	>25	2.35	0.10	>25	0.11	0.06	>25	1.32	6.84	>25	>25
TSG-EHI17B14	>25	>25	0.87	>25	>25	>25	0.14	1.67	>25	0.42	0.04	0.21	>25	>25
TSG-EHI8	>25	1.10	4.38	10.48	1.22	0.02	4.91	0.12	>25	>25	>25	0.11	23.87	>25
TSG-EHI14	<0.01	0.04	1.80	0.05	0.45	0.05	<0.01	0.05	1.18	0.03	0.02	<0.01	0.30	0.90
TSG-EHI40-C18	0.04	0.18	0.08	0.77	1.52	0.25	0.03	0.39	0.63	0.06	0.19	2.05	1.47	0.21
TSG-EHI11	>25	0.63	2.01	>25	1.20	0.57	0.32	0.31	0.37	1.08	0.18	0.04	>25	>25
TSG-EHI21	>25	0.11	0.17	0.19	>25	0.09	0.11	0.17	>25	0.11	0.05	<0.01	1.78	0.80
TSG-EHI26	>25	>25	18.34	>25	1.45	0.71	0.09	0.04	0.03	>25	0.44	0.90	3.56	1.05
TSG-EHI30	>25	1.22	3.65	>25	0.35	<0.01	0.44	0.33	0.14	0.31	0.15	<0.01	3.70	>25
TSG-EHI18	>25	0.97	0.09	0.14	0.56	0.17	0.05	0.18	0.05	0.17	0.07	<0.01	0.51	0.43
TSG-EHI22	>25	0.09	0.39	14.34	2.63	0.25	0.16	1.04	>25	0.92	0.02	0.02	>25	>25
TSG-EHI29	>25	>25	0.49	>25	6.78	4.11	17.11	0.39	0.59	0.26	>25	>25	4.22	>25
TSG-EHI35	>25	0.19	0.22	19.70	0.25	0.11	<0.01	0.02	>25	>25	7.52	>25	1.28	>25
TSG-EHI37	>25	>25	>25	>25	1.00	0.25	0.33	0.26	7.86	>25	0.40	<0.01	2.89	>25
TSG-EHI7	>25	>25	>25	>25	10.33	0.56	0.80	2.32	>25	15.97	0.61	1.80	>25	1.19
TSG-EHI12	>25	0.15	1.74	>25	0.15	0.05	0.03	0.13	0.05	7.50	0.05	0.02	2.20	>25
TSG-EHI20	>25	>25	>25	>25	0.39	0.11	0.49	0.07	0.07	>25	>25	>25	11.51	>25
TSG-EHI23	0.02	0.71	>25	>25	0.87	0.15	1.74	0.22	5.61	8.04	0.24	0.03	3.50	>25
TSG-EHI27	>25	>25	>25	>25	>25	>25	0.76	0.85	>25	>25	>25	0.89	2.37	0.67
TSG-EHI33	<0.01	<0.01	0.53	>25	<0.01	<0.01	0.29	<0.01	>25	4.00	0.20	0.25	>25	>25
TSG-EHI55	>25	>25	>25	>25	1.14	0.31	0.09	0.98	0.23	3.30	1.12	0.31	7.11	0.51
TSG-EHI60	>25	>25	>25	>25	0.11	0.16	0.12	0.07	0.38	>25	>25	>25	9.02	0.35
TSG-EHI61	2.68	>25	0.17	>25	>25	>25	1.16	>25	>25	0.02	0.01	<0.01	1.69	>25
TSG-EHI62	>25	>25	0.04	>25	5.02	0.52	0.13	0.02	14.05	>25	0.46	>25	<0.01	0.02
TSG-EHI63	>25	>25	8.33	>25	4.82	1.59	0.62	0.15	0.99	1.54	>25	>25	5.93	>25
TSG-EHI25	0.07	12.04	>25	>25	>25	5.40	1.62	3.25	23.29	0.33	1.18	>25	>25	>25
TSG-EHI50	>25	>25	>25	>25	9.75	3.68	1.95	1.32	0.94	>25	>25	>25	>25	>25
TSG-EHIPre15	1.73	>25	1.82	5.23	>25	3.59	6.45	4.27	>25	0.32	0.42	0.04	13.38	>25
TSG-EHI51	18.37	>25	>25	>25	0.82	0.14	0.22	0.41	1.25	>25	9.46	>25	>25	>25
TSG-EHI16	15.07	>25	1.80	>25	0.10	0.01	5.24	0.05	0.03	0.24	0.03	<0.01	7.75	0.78
TSG-EHI36	14.99	0.13	>25	11.52	1.37	0.44	>25	0.55	>25	>25	>25	>25	1.47	>25
TSG-EHI34	>25	>25	>25	>25	2.05	0.44	0.12	0.50	1.37	0.14	0.03	<0.01	>25	6.76
TSG-EHI53	<0.01	<0.01	0.22	<0.01	0.94	0.53	0.26	0.32	1.12	>25	>25	>25	0.83	>25
TSG-EHI57	>25	>25	>25	>25	>25	24.32	4.24	1.16	>25	0.07	0.06	0.01	9.85	11.03
TSG-EHI58	3.44	>25	>25	>25	2.16	0.32	0.23	0.45	0.57	>25	>25	15.22	0.80	>25

Table S2. Neutralization breadth and potency (IC50 and IC80) of pseudoviruses expressing contemporary India clade C *envs.*

Viruses	CAP256-VRC26.25	PGDM1400	PG9	PGT145	VRC01	VRC07	1-18	N6	3BNC117	PGT121	10-1074	BG-18	10E8	VRC34.01
TSG-EHI59	>25	>25		0.15	>25	>25	10.55	>25	>25	0.09	0.04	<0.01	1.38	0.30
TSG21Y02A0012	>25	>25	0.72	8.72	1.45	3.15	>25	0.28	>25	2.04	0.62	<0.01	23.24	0.82
TSG21Y02E0018-D18	7.22	4.14	>25	>25	1.24	0.39	1.59	0.08	15.31	0.21	0.36	0.51	>25	0.28
TSG21Y02E0024-D24	<0.01	0.04	0.02	>25	0.31	0.88	1.02	0.26	0.47	>25	>25	>25	1.05	>25
TSG22Y02E0035-DE35	0.09	6.86	>25	4.66	>25	>25	1.23	5.80	>25	1.78	0.17	0.61	6.93	1.55
TSG22Y02E0037-DE37	>25	>25	>25	>25	3.13	0.90	0.26	<0.01	>25	2.18	0.20	<0.01	17.29	6.14
TSG21Y02E0011-D11	>25	>25	2.45	>25	0.69	0.91	1.06	0.52	3.32	0.18	0.22	0.04	>25	0.17
TSG21Y02E0014-D14	<0.01	<0.01	0.07	0.71	0.15	0.06	0.14	0.07	0.94	0.21	0.12	<0.01	0.91	1.17
TSG21S01A0008_STMART07	2.46	23.68	>25	>25	5.35	0.29	0.29	0.41	0.78	1.26	>25	20.62	6.08	>25
TSG21S01A0013-STMART12	>25	11.21	0.43	>25	21.60	3.63	0.90	2.84	0.38	7.37	0.63	0.74	24.94	0.42
TSG21S01A0003-STMART02	>25	9.97	0.04	>25	10.63	2.37		2.39	18.34	0.71	0.77	<0.01	4.56	
TSG21SO1A0014-STM13ART	>25	>25	5.92	>25	10.46	1.34	1.26	3.53	5.55	7.45	1.87	0.47	>25	0.80
TSG21S01E0017-STM16E	0.01	9.41	15.50	19.36	4.91	1.53	0.63	0.73	6.05	2.18	0.35	0.04	9.42	>25
TSG21S01E0001-STM01E	<0.01	<0.01	>25	>25	3.06	1.61	0.06	0.34	>25	4.67	0.11	0.02	23.90	>25
TSG21S01A0002-STMART01	>25	>25	>25	>25	15.91	0.82	0.78	>25	>25	4.67	0.11	0.02	0.68	>25
TSG21S01E0018-STM17E	<0.01	>25	>25	>25	2.38	0.59	0.15	0.59	10.15	1.42	0.37	0.04	6.03	0.29
TSG21S01A0004-C4	>25	>25	>25	>25	4.50	0.28	0.90	0.38	2.01	0.35	0.83	<0.01	17.07	3.05
TSG21S01A0005-C5	<0.01	<0.01	0.56	2.40	15.81	2.01	0.54	0.70	11.13	0.04	0.14	0.03	0.13	2.07
TSG21S01E0033-C33	0.29	>25	0.02	>25	>25	>25	<0.01	0.10	>25	0.30	0.14	<0.01	1.42	>25
TSG21N01N017_C18	>25	>25	>25	>25	>25	>25	>25	>25	>25	**17.07**	2.23	**>25**	16.90	>25
TSG21N01N011-C10	0.05	0.11	0.30	1.05	2.22	0.24	0.25	0.60	0.35	0.86	0.16	0.02	0.71	>25
TSG21N01N029	<0.01	0.35	0.30	0.07	>25	3.51	18.21	0.13	3.46	0.98	0.11	3.04	0.69	0.51
TSG21N01N018	>25	>25	>25	>25	2.90	0.79	>25	0.68	1.54	0.22	0.05	<0.01	5.70	>25
TSG21N01S028	>25	>25	>25	>25	0.38	0.08	0.32	0.04	9.51	>25	1.28	<0.01	4.11	>25
TSG21N01N010	>25	0.03	0.12	0.70	9.81	1.24	0.88	1.34	>25	<0.01	0.02	<0.01	0.73	0.05
TSG21N01N014	>25	>25	>25	>25	5.61	1.43	0.42	1.91	18.57	>25	>25	>25	11.76	1.14
TSG21N01N023	>25	>25	>25	>25	0.46	0.12	0.03	0.27	1.25	>25	>25	>25	16.07	1.84
TSG21N01N031	>25	>25	>25	>25	0.84	0.78	0.04	0.12	>25	>25	>25	>25	5.61	>25
TSG21N01F003	>25	>25	>25	>25	3.25	0.73	0.73	0.58	1.26	>25	>25	>25	8.72	>25
TSG21N01N001CM	<0.01	<0.01	12.22	>25	>25	1.39	0.52	8.02	>25	>25	>25	>25	0.50	0.26
TSG21N01N013	<0.01	3.86	>25	>25	4.45	0.47	0.39	>25	>25	>25	0.15	0.04	>25	>25
TSG21N01N015	11.21	0.30	1.63	11.53	0.77	0.05	0.07	<0.01	3.02	<0.01	<0.01	<0.01	2.43	>25
TSG21N01N025	>25	<0.01	22.42	>25	>25	17.73	0.08	7.30	>25	>25	0.73	>25	0.09	0.17
TSG21N01N026	>25	<0.01	5.33	9.61	8.01	3.00	0.17	1.39	2.50	>25	1.15	0.15	0.22	>25
TSG21N01S007	>25	>25	>25	>25	5.28	0.95	12.73	3.66	>25	8.12	1.73	0.04	5.74	5.39
TSG21N01S027	0.41	0.12	>25	0.20	0.59	0.03	0.24	0.28	>25	>25	>25	>25	1.53	>25
TSG21N01S030	>25	>25	>25	>25	>25	4.58	7.98	4.26	>25	1.57	4.26	0.14	>25	0.82
TSG21N01S100	<0.01	>25	0.20	>25	2.65	0.57	1.25	0.12	3.30	0.19	0.16	0.06	1.73	0.64
TSG21N01S055	<0.01	>25	1.42	11.89	17.00	4.46	10.67	1.64	9.76	>25	>25	>25	19.23	0.36
TSG21N01S062	0.01	>25	>25	1.07	0.86	0.21	0.44	0.33	>25	4.36	0.62	0.11	8.24	10.33
TSG22Y03E0010-B10	<0.01	0.24	0.27	4.46	0.05	0.03	0.09	0.09	0.13	0.06	0.06	0.01	2.05	0.29
TSG22Y03A0015-B15	<0.01	0.02	8.92	8.85	0.10	0.22	<0.01	0.03	>25	0.08	0.05	0.01	11.02	0.35
TSG22Y03A0022-B22	<0.01	<0.01	>25	>25	0.05	0.16	0.75	0.02	2.01	0.06	0.02	0.12	4.65	0.45
TSG22Y03A0031-B31	<0.01	<0.01	0.33	6.02	>25	0.31	0.03	0.03	>25	0.21	0.12	0.02	2.17	0.09
TSG22Y03A0032-B32	0.13	0.53	4.76	3.73	0.07	0.08	0.21	0.04	0.30	0.43	0.28	0.05	10.67	0.73
TSG22Y03E0019-B19	>25	>25	>25	>25	1.46	0.45	0.70	0.07	1.50	0.03	0.53	<0.01	2.92	>25
TSG22Y03E0023-B23	0.49	1.14	>25	0.08	>25	>25	>25	>25	>25	0.50	0.43	0.04	0.45	>25

Table S2. Neutralization breadth and potency (IC50 and IC80) of pseudoviruses expressing contemporary India clade C *envs.*

Viruses	CAP256-VRC26.25	PGDM1400	PG9	PGT145	VRC01	VRC07	1-18	N6	3BNC117	PGT121	10-1074	BG-18	10E8	VRC34.01
TSG23Y07A0012-A12	0.14	6.50	>25	2.27	24.06	1.50	1.07	0.74	2.36	>25	>25	>25	16.93	>25
TSG23Y07A0013-A13	>25	0.10	3.79	0.29	0.43	0.25	0.38	0.18	0.97	0.48	0.32	0.06	1.61	>25
TSG23Y07A0015-A15	>25	0.28	16.26	11.88	>25	0.17	0.52	0.12	>25	0.60	0.23	0.04	1.19	>25
TSG23Y06A012-V12	<0.01	0.47	0.13	7.01	>25	1.76	>25	10.11	1.13	1.21	0.71	1.37	0.08	>25
TSG23Y06A015-V15	<0.01	<0.01	2.98	>25	7.76	2.59	8.91	1.29	>25		0.85	0.03	3.68	3.31
TSG23Y06E001-V1	>25	1.03	2.30	7.16	2.65	0.90	1.15	0.37	9.73	0.24	0.13	0.03	5.19	>25
TSG23Y06A003-V3	>25	<0.01	0.19	1.56	2.05	1.08	0.36	0.59	2.51	0.29	0.08	0.02	0.48	0.13
TSG23Y06A004-V4	4.03	>25	0.29	>25	>25	3.55	>25	5.79	>25	20.72	0.34	0.01	>25	>25
TSG22Y04A0011-H11	>25	>25	4.59	1.36	2.90	0.37	0.30	0.47	1.17	4.24	0.98	8.50	21.30	>25
TSG22Y04A0015-H15	0.11	19.20	0.68	0.62	4.71	1.61	1.20	1.14	1.34	4.24	0.74	6.40	5.66	0.42
TSG22Y04A0016-H16	0.58	<0.01	0.09	<0.01	>25	0.41	0.14	0.06	>25	>25	0.69	>25	>25	16.87
TSG22Y04A0020-H20	>25	<0.01	0.62	<0.01	0.07	0.02	0.03	<0.01	0.64	0.04	0.01	0.02	1.42	>25
TSG22Y04A0002-H2	0.77	1.29	3.82	5.39	>25	18.82	>25	4.84	14.44	>25	>25	19.41	0.80	>25
TSG22Y04A0003-H3	<0.01	<0.01	0.03	16.79	<0.01	<0.01	0.07	<0.01	0.11	0.46	6.24	>25	0.07	0.13
TSG22Y04A0004-H4	>25	>25	>25	2.24	>25	7.06	5.94	4.52	>25		0.12	0.12	>25	>25
TSG22Y04A0005-H5	<0.01	9.80	0.75	2.38	19.31	2.73	0.85	1.56	>25	0.41	0.21	0.03	9.33	0.83
TSG22Y05A0018-BL18	>25	5.44	>25	1.44	>25	>25	>25	>25	>25	>25	>25	>25	6.45	>25
TSG22Y05A0021-BL21	>25	>25	5.38	>25	0.95	0.42	0.18	0.32	>25	1.29	0.20	<0.01	>25	>25
TSG22Y05A0002-BL2	<0.01	>25	0.17	>25	0.98	0.20	20.10	0.20	0.92	1.26	0.48	0.11	>25	>25
TSG22Y05E0033-BL33	>25	>25	>25	>25	1.15	0.17	0.35	0.16	>25	0.27	0.14	0.14	15.64	>25
TSG22Y05E0043-BL43	<0.01	<0.01	0.14	0.20	20.86	4.73	1.37	4.42	10.39	7.54	0.77	0.05	>25	>25
TSG22Y05A0006-BL6	>25	>25	2.73	>25	>25	>25	>25	>25	>25	0.03	<0.01	1.84	1.13	>25

The values indicate neutralization titers (µg/mL) that conferred 80% pseudovirus neutralization in TZM-bl cells.
